# Development of a Universal Cloning System for Reverse Genetics of Human Enteroviruses

**DOI:** 10.1128/spectrum.03167-22

**Published:** 2023-01-18

**Authors:** Won-Suk Choi, Sol Oh, Khristine Joy C. Antigua, Ju Hwan Jeong, Beom Kyu Kim, Yu Soo Yun, Da Hyeon Kang, Seong Cheol Min, Byung-Kwan Lim, Won Seop Kim, Ji-Hyuk Lee, Eung-Gook Kim, Young Ki Choi, Yun Hee Baek, Min-Suk Song

**Affiliations:** a Department of Microbiology, Chungbuk National University College of Medicine and Medical Research Institute, Cheongju, Chungbuk, Republic of Korea; b Animal Health and Welfare Division, Bureau of Animal Industry (BAI), Department of Agriculture (DA), Quezon City, Philippines; c Department of Biomedical Science, Jungwon University, Goesan-gun, Chungbuk, Republic of Korea; d Department of Pediatrics, College of Medicine and Medical Research Institute, Chungbuk National University, Cheongju, Chungbuk, Republic of Korea; e Department of Biochemistry, Chungbuk National University College of Medicine and Medical Research Institute, Cheongju, Chungbuk, Republic of Korea; f Center for Study of Emerging and Re-emerging Viruses, Korea Virus Research Institute, Institute for Basic Science (IBS), Daejeon, Republic of Korea; g Microuni Co., Ltd., Cheongju, Chungbuk, Republic of Korea; University of Siena

**Keywords:** enterovirus, reverse genetics, infectious clone, universal cloning, universal RACE-PCR

## Abstract

Enteroviruses (EVs) have been associated with several human diseases. Due to their continuous emergence and divergence, EV species have generated more than 100 types and recombinant strains, increasing the public health threat caused by them. Hence, an efficient and universal cloning system for reverse genetics (RG) of highly divergent viruses is needed to understand the molecular mechanisms of viral pathology and evolution. In this study, we generated a versatile human EV whole-genome cDNA template by enhancing the template-switching method and designing universal primers capable of simultaneous cloning and rapid amplification of cDNA ends (RACE)-PCR of EVs. Moreover, by devising strategies to overcome limitations of previous cloning methods, we simplified significant cloning steps to be completed within a day. Of note, we successfully verified our efficient universal cloning system enabling RG of a broad range of human EVs, including EV-A (EV-A71), EV-B (CV-B5, ECHO6, and ECHO30), EV-C (CV-A24), and EV-D (EV-D68), with viral titers and phenotypes comparable to those of their wild types. This rapid and straightforward universal EV cloning strategy will help us elucidate molecular characteristics, pathogenesis, and applications of a broad range of EV serotypes for further development of genetic vaccines and delivery tools using various replication systems.

**IMPORTANCE** Due to the broad spread, incidence, and genetic divergence of enteroviruses (EVs), it has been challenging to deal with this virus that causes severe human diseases, including aseptic meningitis, myocarditis, encephalitis, and poliomyelitis. Therefore, an efficient and universal cloning system for the reverse genetics of highly divergent EVs contributes to an understanding of the viral pathology and molecular mechanisms of evolution. We have simplified the important cloning steps, hereby enhancing the template-switching method and designing universal primers, which enable the important cloning steps to be completed in a day. We have also successfully demonstrated recovery of a broad range of human EVs, including EV-A to -D types, using this advanced universal cloning system. This rapid and robust universal EV cloning strategy will aid in elucidating the molecular characteristics, pathogenesis, and applications of a wide range of EVs for further development of genetic vaccines and antiviral screening using various replication systems.

## INTRODUCTION

The family *Picornaviridae* contains viruses with a nonenveloped single-stranded RNA genome of positive polarity. Within this family, the genus *Enterovirus* (EV) consists of 15 species and more than 100 types that can cause a wide range of clinical diseases, such as common cold, hand, foot, and mouth disease (HFMD), wet hemorrhagic conjunctivitis, aseptic meningitis, myocarditis, encephalitis, poliomyelitis, and others (1, 2). Recently, there have been a large number of reports of nonpolio enteroviruses causing neurological and respiratory diseases in humans (3, 4). However, with a limited understanding of the evolutionary dynamics of EVs, control strategies and vaccine development remain inadequate (5). Structurally, the RNA genome of EVs is approximately 7,400 to 7,500 nucleotides in length, including untranslated regions (UTR) such as the 5′ UTR and 3′ UTR. During viral gene expression, the viral RNA is encoded in a single large open reading frame (ORF) to form polyproteins, which are then cleaved during translation. The EV polyprotein is divided into three separate parts: polyprotein 1 (P1), including structural proteins (VP1 to VP4), and P2 and P3, including nonstructural proteins (2A to 2C and 3A to 3D) that are important in viral protein processing and replication processes (6).

Over time, recombinant DNA techniques have enabled detailed approaches to elucidate molecular features of viral infection and pathogenesis. Research activities related to viral etiology, genomic functions, viral infection, replication, and vaccine development are generally impossible without reverse genetics (RG) tools capable of manipulating the viral genome (7). In 1981, Racaniello and Baltimore developed the first RG tool for picornaviruses using a cDNA infectious clone of the poliovirus genome in the pBR322 vector (8). Since then, a wide range of studies have established RG systems for hepatitis A virus (9), poliovirus (PV) (10), enterovirus A71 (EV-A71) (11), coxsackievirus A16 (CV-A16) (12), CV-A6 (13), CV-B3 (14), CV-B6 (15), echovirus 30 (ECHO30) (16), and ECHO25 (17). However, considering the presence of more than 100 types of human EVs, these RG methods remain limited to applications that are possible only for the same serotype or after complete sequence analysis.

In the past, most poliovirus infectious cDNA clones were cloned downstream of a bacterial phage promoter such as the SP6 or T7 promoter. This approach involves *in vitro* RNA transfection into cells or electroporation after RNA transcription (18–20). Recently, Tan et al. in their study improved the cloning method for generating EV-A71 infectious clone constructs using eukaryotic (cytomegalovirus [CMV]) and bacteriophage (T7) promoters with unique self-cleavage ribozyme sequences (21). However, this approach remains limited to EV-A71. Nonetheless, in all dual-promoter systems used for EV cloning, the efficiency of the T7 promoter in generating RNA-based RG viruses remains low (21). Hence, in this study, we developed a recombinant DNA technology method that could be applied to a broad range of EVs.

Generally, the development of the RG method involves tedious cloning steps. Other commonly encountered problems in performing traditional cloning methods include the need for accurate sequencing of the viral genome before the generation of the infectious clone. In addition, due to the poor efficiency of reverse transcriptase, the difficulty of amplifying the whole genome, especially long RNA viruses, is often experienced (22–24). Moreover, in the absence of primers that universally amplify the full-length EV genome, the correct sequence information for the 5′ and 3′ ends of the viral genome should be analyzed through rapid amplification of cDNA ends (RACE)-PCR. In this process, additional genome-specific primers are required. RACE-PCR can extend the tag to the 5′ and 3′ ends through reverse transcription and template switching (TS) to the CCC residues generated after reverse transcription of the poly(A) portion of viral mRNA and Moloney murine leukemia virus (MMLV)-derived reverse transcriptase. cDNA with tags extended at the 5’ and 3’ ends is then amplified by PCR and viral genome-specific primers while preserving the 5′ end and the 3′ end of the viral genome. However, since template switching is performed not only on the EV genome but also on off-target mRNAs, it can lead to many nonspecific amplicons. Overall, the complexity of steps, time-consuming nature, and lack of cost efficiency create significant disadvantages.

To overcome the above-described limitations of performing sequence-dependent cloning, we devised several strategies for rapid and easy universal cloning of most human EVs (EV-A, -B, -C, and -D). We reconstructed a pUC-CMV/T7 vector comprising a dual promoter to generate a recombinant RNA virus using an infectious clone system derived from both plasmid DNA and *in vitro* RNA transcripts. We also simplified the process of amplification of the whole genome of human EVs within a single reverse transcription (RT)-PCR by designing universal primers and using a sequence-independent cloning method. Thus, prior genomic sequencing is unnecessary. In addition, through reverse transcription with the improved TS method for cDNA construction, the end product synthesized could be utilized simultaneously for full-genome PCR and RACE-PCR. Using these strategies, we constructed a universal cloning method for reverse genetics of human EV species. It allows for successful generation of EV infectious clones within a shorter period (it takes 1 day before the plasmid transformation step) as well as recombinant viruses using infectious clones derived from both DNA and RNA. This developed EV universal cloning strategy will significantly contribute to molecular studies of a broad range of serotypes of EV.

## RESULTS

### Construction of a universal cloning vector (pUC19-CMV/T7) for enterovirus reverse genetics.

**FIG 1:**
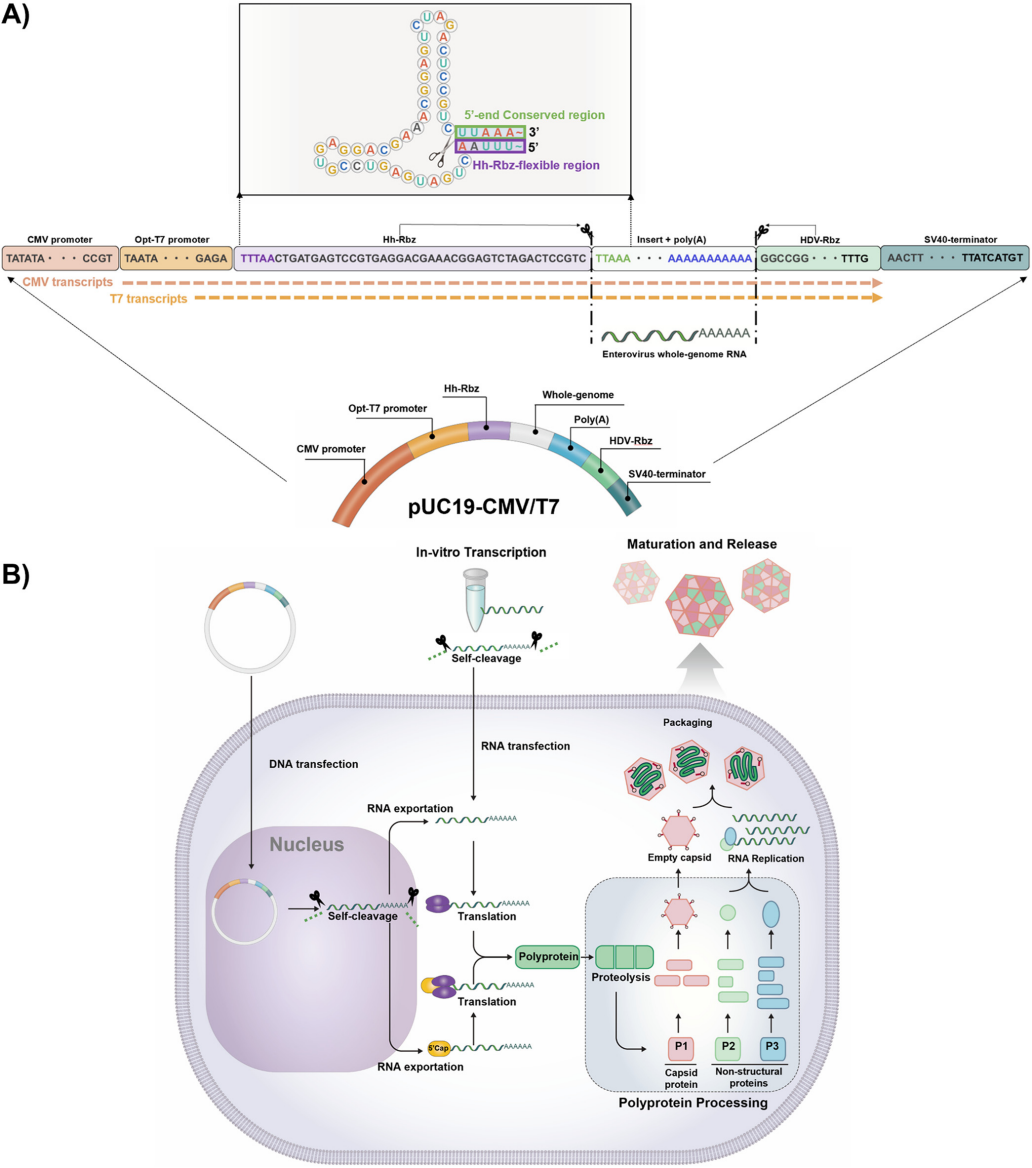
Construction of the pUC19-CMV/T7 dual-promoter vector for universal enterovirus (EV) cloning. (A) Configuration of the dual-promoter system. The optimized T7 promoter sequence was extended after the cytomegalovirus (CMV) promoter sequence. Two dotted arrows indicate the transcription initiation site of the CMV and T7 promoters. RNAs transcribed by the promoters are cleaved to correct ends of the EV RNA genome by hammerhead ribozyme (Hh-Rbz) and hepatitis delta virus ribozyme (HDV-Rbz) as indicated by thin arrows and scissors, with estimated secondary structure of Hh-Rbz universally available for EV in the box. (B) Schematic of the mechanism of EV infectious clones.

To establish a universal cloning method for EV infectious clones, we initially designed a vector to improve versatility while avoiding the need to regenerate cloning vectors for each strain. To drive gene expression with dual functionality, we incorporated ubiquitous promoters, eukaryotic CMV and bacteriophage T7 (for *in vitro* transcription), into one expression vector, enabling virus recovery from both RNA and DNA constructs (Fig. 1). Although promoters have been widely used in RG because of their advantages in the transcription process, extra nucleotides generated and incorporated at the 5′ and 3′ ends of the transcripts might interfere with recognizing the terminus by viral RNA-dependent RNA polymerase (RdRp) complex for replication. To generate congenital viral RNA without additional nucleotide sequences, the construct was equipped with two self-cleaving ribozymes (Rbz): hammerhead (Hh)-Rbz and hepatitis delta virus (HDV)-Rbz (Fig. 1A). Autocatalytic Hh-Rbz was introduced between the optimal T7 promoter and the start of the antigenome to induce self-cleavage immediately before the start of the viral genomic transcription. The inclusion of Hh-Rbz at the 5′ end of the antigenome increased the efficiency of cleaving the exact viral genomic terminus with robust vector utility (Fig. 1A). Using a reverse transcription-quantitative PCR-based assay (25, 26) to determine the cleavage efficiency of Hh-Rbz against RNA isolated from transfected cells, the cleavage efficiency was determined to be within a range of 93.93 to 96.8% (see Fig. S1 in the supplemental material). Hh-Rbz was designed to recognize five nucleotides, UUAAA, at the 5′ end of the EV genome and cleave off unnecessary nucleotides hanging at the 5′ end. *In silico* analysis of available sequences in the VIPBR database (*n* = 2,349) revealed that all types of human EVs shared UUAAA nucleotides at the 5′ end, with a high conservation rate (99.7%). Therefore, Hh-Rbz recognizing sequences designed in the vector does not require additional changes. It can be used universally for EVs (Fig. 1A). We used another autocatalytic cleavage site in the HDV-Rbz sequence downstream of the antigenome located between the poly(A) and simian virus 40 (SV40) terminators to ensure the generation of the exact 3′ end of the antigenome. Therefore, our dual-promoter vector system can transcribe the viral RNA genome in the cell transfected with DNA plasmid and in a tube. Transcribed RNAs are then autocatalytically trimmed in the cell into its intrinsic form of the viral genome, enabling recombinant virus generation (Fig. 1B).

### Construction of enterovirus whole-genome cDNA for universal cloning.

**FIG 2:**
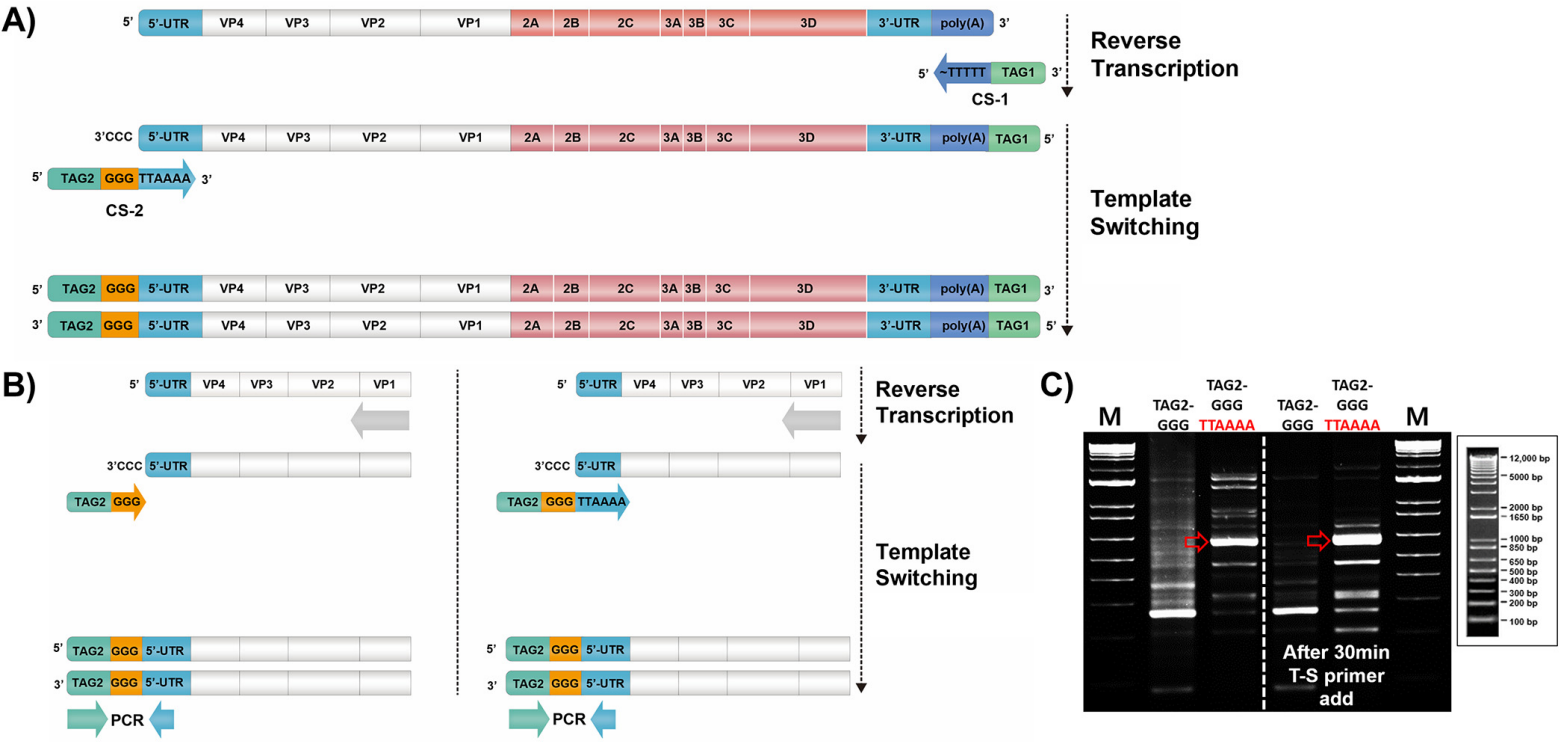
Generation of enterovirus whole-genome cDNA for universal cloning and RACE-PCR. (A) Reverse transcription was performed using a designed primer with a dT-rich-extended sequence (CS-1) coupled with a template-switching (CS-2) primer used to generate EV whole-genome cDNA with 5′- and 3′-end tags. (B) Schematic diagram of the design principle for comparison of EV-specific template-switching and typical template-switching efficiencies. The Tag2-GGG primer is generally a template-switching primer. The Tag2-GGG-TTAAA primer is an EV-specific template switching primer for improved EV specificity. The template switching induction period was controlled either simultaneously with reverse transcription or 30 min later. (C) Gel electrophoresis analysis of 5′ UTR RACE-PCR (CV-B5) to compare the two strategies. The arrow shows the impact of the late addition of TS primer in minimizing the nonspecific band amplification during cDNA synthesis. Lane M, 1-kb DNA size ladder.

Generation of a whole-genome cDNA of EVs with 5′- and 3′-end tags was initially achieved through the designed method of universal EV reverse transcription with improved template switching (TS). This study used TS at the 5′ end of the RNA transcript to construct an EV full-length genomic cDNA library with 5′- and 3′-end tags (Fig. 2A). Two tagging primers, CS-1 and CS-2, were designed for RT and TS to generate whole-genome cDNA (Fig. 2A and Table 1), which was later used for EV full-genome insert amplification and 5′- and 3′-end RACE-PCR of the EV genome. The lock docking primer (CS-1), which has an extended tag sequence at the end of dT-rich bases (50 nucleotides), was the primer used for the reverse transcription. Tag1 of CS-1 primer contained a 15-bp sequence of the 5′ end of HDV-Rbz of the universal vector for infusion cloning. In addition, to increase the TS efficacy, the six nucleotides TTAAAA at the 5′ end of EV genome (more than 99.7% homology among EV types A to D) were further extended after the GGG sequence of the traditional TS primer to minimize the off-target binding and make it more specific for EVs (Fig. 2B and Table 1). The TS efficacy was improved with the TTAAAA-extended TS primer (CS-2) compared to when the traditional TS primer was used (Fig. 2C, left). TS efficacy was also enhanced when the TS primer was added 30 min after the RT reaction was initiated (Fig. 2C, right). The TS primer (CS-2), which contained the GGG and TTAAAA nucleotide sequences, enabled template switching and improved primer binding specificity for the EV genome.

**TABLE 1:** List of primers of the universal cloning system for reverse genetics of enterovirus^*a*^

Primer name	Primer code	Sequence (5′–3′)	Size (bp)	Comment
Oligo dT+Tag1	CS-1	GCTGGGACCATGCCGGCCTTTTTTTTTTTTTTTTTTTTTTTTTTTTTTTTTTTTTTTTTTTTTTTTTT	68	Reverse primer for reverse transcription
Universal *Enterovirus* TS oligo+Tag2	CS-2	CACCATCGATGTCGACACGCGTCGGGttaaaa	32	Enterovirus-specific template-switching primer
TS oligo	CS-3	CACCATCGATGTCGACACGCGTCGGG	26	Commonly used template-switching primer
pUC19-CMV/T7-Vec-F	CV-1	TAGAGGATCCCCGGGTACC	19	Primer set for pUC19-CMV/T7 cloning linearized vector
pUC19-CMV/T7-Vec-R	CV-2	GAGTCGACCTGCAGGCAT	18	
pUC19-CMV/T7-ins-F	CV-3	CCTGCAGGTCGACTCCGTTACATAACTTACGGTAAATG	38	Primer set for pUC19-CMV/T7 cloning insert
pUC19-CMV/T7-ins-R	CV-4	CCCGGGGATCCTCTAACATGATAAGATACATTGATGAG	38	
M13-F (-20)	M13-F	GTAAAACGACGGCCAG	16	Primer set for vector clone selection by colony PCR
M13-R (-40)	M13-R	CAGGAAACAGCTATGAC	17	
Universal *Vector for Enterovirus*-F	UC-1	GGCCGGCATGGTCCCAGCCTCCTCGCTGGCGCCGGCTGGGCAACATT	47	Primer set for universal enterovirus cloning linearized vector
Universal Vector for *Enterovirus*-R	UC-2	GACGGAGTCTAGACTCCGTT	20	
Universal *Enterovirus* Insert F	UC-3	AGTCTAGACTCCGTC*TTAAAACAGCCTGTGGGTT* (flexible region see Supplementary Table 2)	34	Forward primer for universal enterovirus cloning insert
Universal *Enterovirus* Insert R	UC-4	GCTGGGACCATGCCGGCCTTTTT	18	Reverse primer for universal enterovirus cloning insert
Universal *Enterovirus* 5′ UTR RACE-PCR-R	RP-1	ATTGTCACCATAAGCAGCCAATA	23	Primer set for enterovirus 5′ UTR RACE-PCR
Tag2-F	RP-2	CATCGATGTCGACACGCGT	19	
Universal *Enterovirus* 3′ UTR RACE-PCR-F	RP-3	RTVCARTTCAAGWSCAAA	18	Primer set for enterovirus 3′ UTR RACE-PCR
Tag1-R	RP-4	GCTGGGACCATGCCGGCCTTTTT	18	
*Enterovirus* A to D 5′ UTR RACE- Sanger Primer-R	RP-5	CCCAAAGTAGTCGGTTCC	18	Primer for universal enterovirus 5′ UTR Sanger sequencing
*Enterovirus* A 3′ UTR RACE-PCR Sanger Primer-F	RP-6	GTVCTTGGHGGRATGCC	17	Primer for universal enterovirus A species 3′ UTR Sanger sequencing
*Enterovirus* B 3′ UTR RACE-PCR Sanger Primer-F	RP-7	TRATYATGACWCCAGC	16	Primer for universal enterovirus B species 3′ UTR Sanger sequencing
*Enterovirus* C 3′ UTR RACE-PCR Sanger Primer-F	RP-8	CCMRADCAGGDAAAGAB	17	Primer for universal enterovirus C species 3′ UTR Sanger sequencing
*Enterovirus* D 3′ UTR RACE-PCR Sanger Primer-F	RP-9	CYMTGACMCCWGCDGA	16	Primer for universal enterovirus D species 3′ UTR Sanger sequencing

a The lowercase indicates six conserved nucleotides at the 5’ end of enterovirus genome, and the italicized and underline indicates the flexible region for universally amplify the enterovirus genome. The flexible regions for each enterovirus type are listed in Supplementary Table 2.

### Establishment of the simplified and universal cloning method for enterovirus infectious clone.

**FIG 3:**
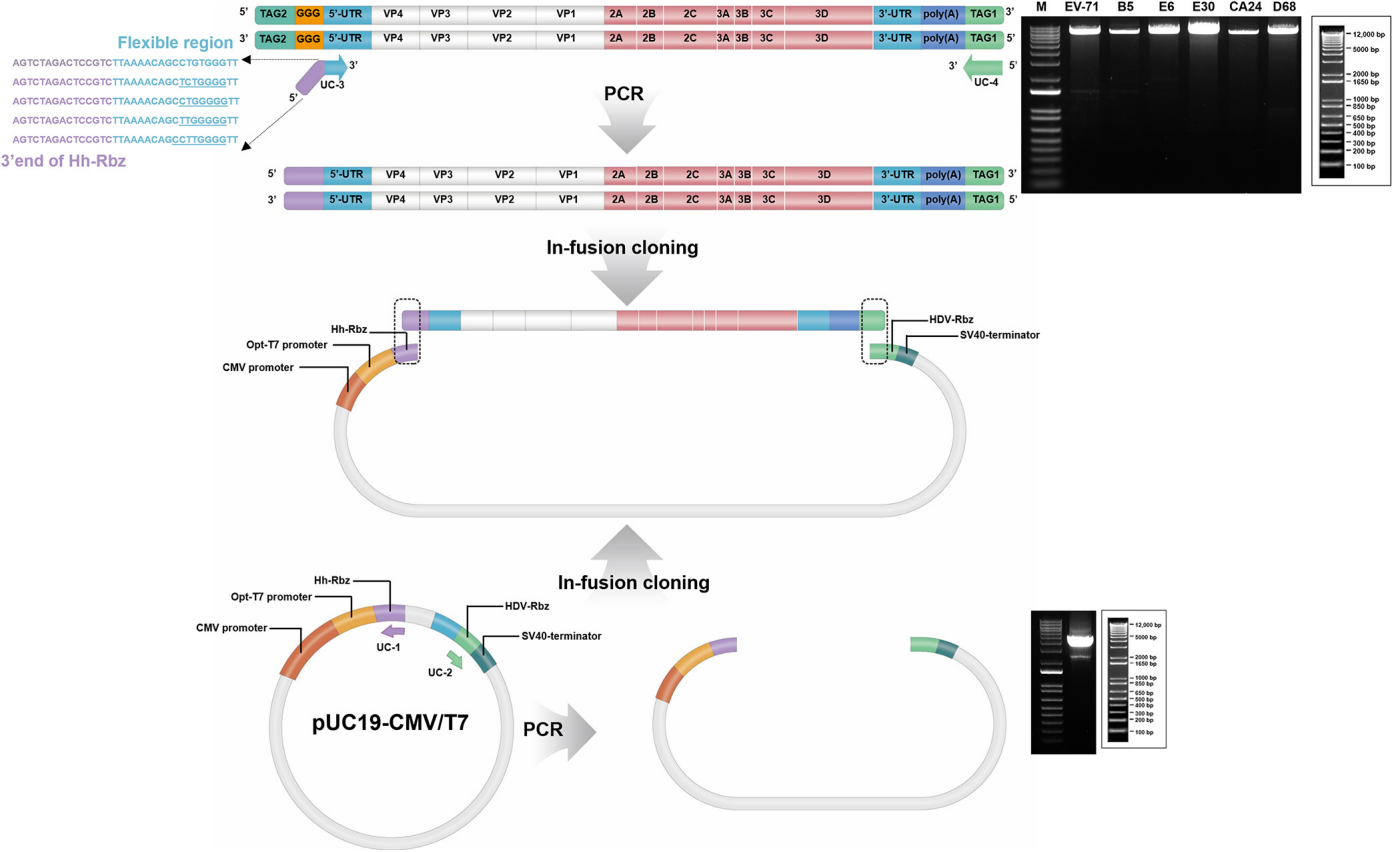
Primer design and preparation of Universal enterovirus vector and insert. Primers were designed to universally amplify EV whole-genome inserts. A total of 2,349 whole-genome sequences from the 5′ UTR to the 3′ UTR of EV-A (*n* = 737), EV-B (*n* = 260), EV-C (*n* = 804), and EV-D (*n* = 548) were downloaded from the Virus Pathogen Database and Analysis Resource (ViPR) research database site (https://www.viprbrc.org) and aligned to verify the conservation of EV 5′ and 3′ ends. Identified regions with more than 88% conservation were used for designing the universal EV insert primers (UC-3 containing the flexible region in bold italic and UC4). The 5′-end insert primer is extended to the Hh-Rbz (light purple) and the 3′-end insert primer is extended to HDV-Rbz (Tag1). Both the 5′ and 3′ ends of insert primer regions are overlapped with the 3′ end (sequence in light purple located in Hh-Rbz) and 5′ end (sequence in blue sequences located at the ends of the universal EV vector). The designed cloning primers UC-1 and UC-2 were utilized to prepare the linearized pUC19-CMV/T7 vector for universal EV cloning. The cloning vector construct had a dual-promoter system equipped with two autocleaving ribozymes placed at both ends of the antigenome, Hh-Rbz and HDV-Rbz, and an SV40 terminator. Validation analysis of EV whole-genome amplicons (EV-A71, CV-B5, ECHO6, ECHO30, CV-A24, and EV-D68) (upper right) and linearized universal EV cloning vector (lower right) are shown. All reaction mixtures were separated on a 0.9% agarose gel and visualized using gel red staining. Lane M, 1-kb DNA size ladder. All vector-insert cloning (CV) primers mentioned in the schematic diagram can be found in Table 1.

Linearized plasmid was generated for efficient PCR amplification of the universal cloning vector by restriction digestion at the AgeI restriction site downstream from the CMV/T7-HhRbz construct while avoiding back transformation of the pUC19-CMV/T7 vector, which was used as the PCR template (Fig. S2). The linearized universal cloning vector was then amplified using UC-1 and UC-2 primer sets (Fig. 3). The 5′ and 3′ ends of the PCR fragment included Hh-Rbz and HDV-Rbz, respectively (Fig. 3).

To universally amplify the whole genomes of EV-A to -D, primers (UC-3 and UC-4) were designed by targeting conserved portions of the 5′ and 3′ ends of the EV whole-genome segment. The flexible regions of the UC-3 primer and the corresponding strains for which the sequence is applicable are shown in Table S1. *In silico* analysis of the EV whole-genome sequences (*n* = 2,349 [Table S2]) revealed that > 90% of the analyzed sequences had conserved nucleotide sequences (18 bp), particularly at the 5′ ends of EV-A to -D (Fig. 3). Species-specific conserved nucleotide sequences were found at the 5′ end for EV-A (>91%; *n* = 737), EV-B (>88%; *n* = 260), EV-C (>92%; *n* = 804), and EV-D (>99%; *n* = 548). Hence, primers were designed by targeting the conserved region of the 5′ end specifically for each species. These primers were used for infusion cloning, which targeted the Hh-Rbz sequence before the 5′ UTR and the Tag1 (15-bp sequence of the 5′ end of HDV-Rbz) sequence behind the poly(A) (Fig. 3).

An in-fusion cloning method was used to clone the EV whole genome. A primer with a 15-bp extension of the 5′- or 3′-end sequence of the linearized universal vector was designed to target the universal region at the EV’s 5′ or 3′ end. The PCR product (insert fragment) overlapped the vector by 15 bp (Fig. 3 and Table 1). Results from using the generated tagged EV whole-genome cDNA confirmed that the designed universal EV whole-genome PCR primers could amplify the whole-genome segment of EV- A to -D (EV-A71, CV-B5, ECHO6, ECHO30, CV-A24, and EV-D68 in the current study) with a single PCR (Fig. 3). To evaluate the stability of the pUC19-CMV/T7 plasmid including the whole enterovirus genome, a total of 10 serial passages were performed and the plasmids extracted from each passage were fully sequenced. Sequencing analysis revealed identical sequences of all plasmids, supporting the stability of the universal cloning vector carrying the whole enteroviral genome (data not shown).

### Universal RACE-PCR.

**FIG 4:**
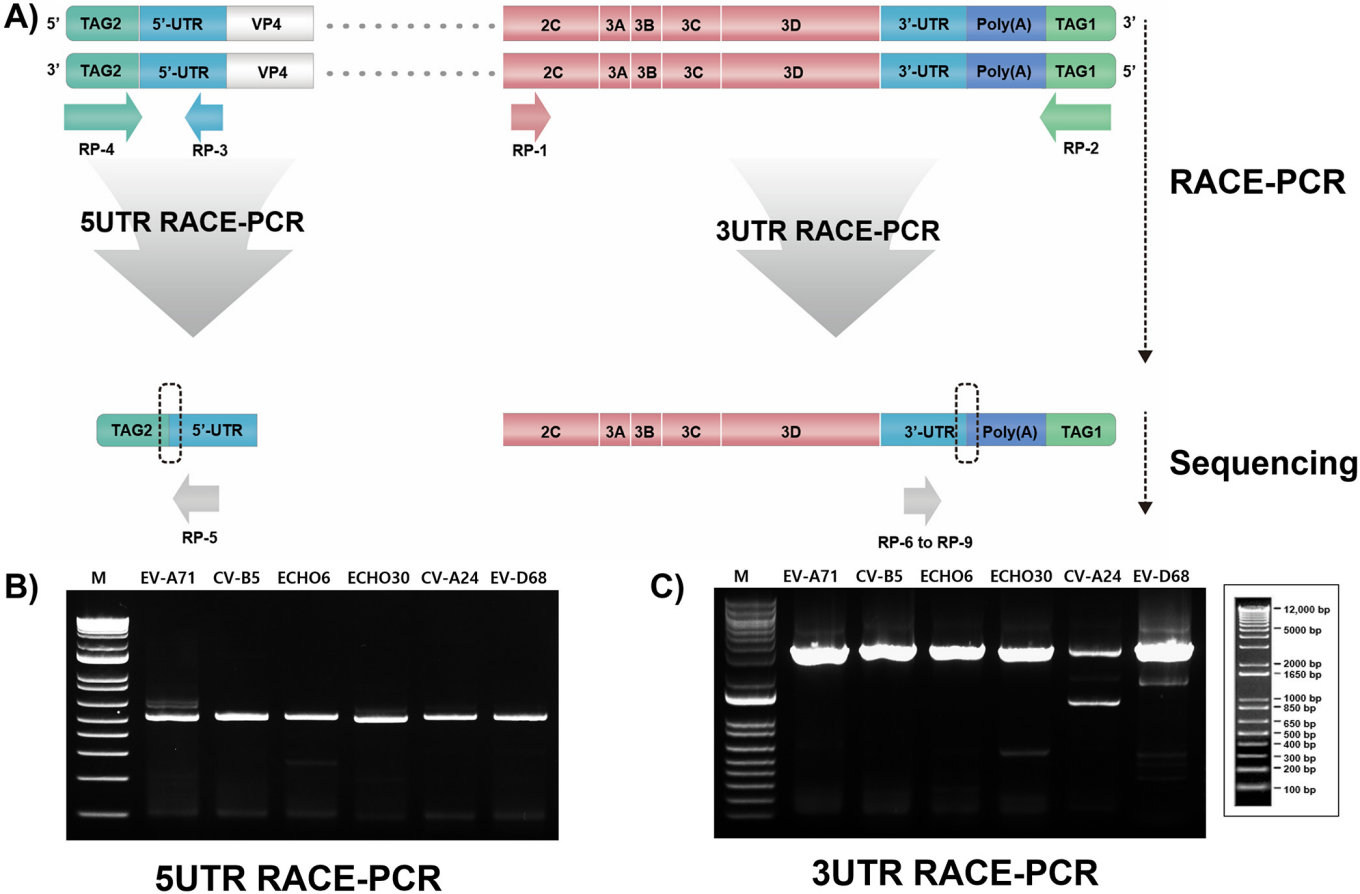
Schematic illustration and amplicon validation of the universal enterovirus RACE-PCR strategy. (A) EV universal RACE-PCR method and universal primer design principle schematic diagram. The dotted box indicates the primers’ targeted region in the 5′ UTR and 3′ UTR of the EV whole-genome cDNA. (B and C) Gel electrophoresis analysis of EV universal 5′ UTR RACE-PCR and 3′ UTR applied to EV-A (EV-A71), EV-B (CV-B5, ECHO6, and ECHO30), EV-C (CV-A24), and EV-D (EV-D68). All reaction mixtures were separated on 0.9% agarose gels and visualized using gel red staining. Lane M, 1-kb DNA size ladder.

Universal RACE-PCR primers were designed so that RACE-PCR could be universally applied to broad human EVs using the synthesized full-length genomic cDNA. To verify the 5′- and 3′-end sequences of the viral genomic RNA of the wild-type (WT) virus, the same tagged EV whole-genome cDNA was used for RACE-PCR. Initially, RACE-PCR primers (RP-1 with RP-2 and RP-3 with RP-4) were designed for broad human EVs (A, B, C, and D), targeting the highly conserved 5′ UTR and 3C region of the EV genome (Table 1). We also designed a more specific EV RACE-PCR Sanger primer with enhanced binding stability for Sanger sequencing (Fig. 4A). Furthermore, the desired PCR band was obtained through the 5′ UTR RACE-PCR strategy using tagged EV whole-genome cDNA (EV-A71, CV-B5, ECHO6, ECHO30, CV-A24, and EV-D68) (Fig. 4B). The 3′ UTR RACE-PCR also proceeded as expected with the designed strategy. We obtained the desired PCR bands as shown in Fig. 4C. The RACE-PCR products were processed for Sanger sequencing using the designed sequencing primers (RP-5 and RP-6 to -9). We confirmed that all sequences of infectious clones used in this study were the same as the 5′ UTR/3′ UTR sequences of WT virus RNA.

### Virus recovery from enterovirus infectious clones.

To test the cloning efficiency of the designed universal EV cloning method, an infused mixture of the universal cloning vector and EV insert was transformed into competent cells. From 10 randomly picked colonies (*n* = 10), each virus was confirmed to have positive cloning efficiency: EV-A71 (*n* = 8/10), CV-B5 (*n* = 6/10), ECHO6 (*n* = 7/10), ECHO30 (*n* = 8/10), CV-A24 (*n* = 5/10), and EV-D68 (*n* = 8/10) (Table 2). Each positive clone underwent deep sequencing using the whole-genomic segment. The sequencing results showed that each clone was 100% homologous to the parental strain, meaning that the clone was mutation free (Table 2).

**TABLE 2:** Confirmation of the designed universal EV cloning method efficiency in generating infectious clones

Virus name	Length (nucleotides)	EV species	Colonies picked/total observed colonies	Positive colonies confirmed by colony PCR/total picked colonies^*a*^ (%)^*b*^	NGS analysis^*c*^		Rescue confirmation (DNA/RNA)
					No. of determined reads	No. of determined reads mapping against the wild type (%)	
EV-A71	7,400	A	10/41	8/10 (80)	135,842	135,842 (100)	+/+
CV-B5	7,402	B	10/45	8/10 (60)	186,241	186,241 (100)	+/+
ECHO6	7,418	B	10/38	6/10 (70)	125,654	125,654 (100)	+/+
ECHO30	7,428	B	10/36	7/10 (70)	92,165	92,165 (100)	+/+
CV-A24	7,458	C	10/64	5/10 (50)	112,267	112,267 (100)	+/+
EV-D68	7,333	D	10/45	8/10 (80)	86,253	86,253 (100)	+/+

a From the picked positive colonies, one sample of each EV serotype was subjected to further analysis using deep sequencing and rescue confirmation.

b Cloning efficiency was calculated based on the method described by Beaty et al. (26).

c Deep sequencing was performed using Illumina’s Nextera XT DNA library preparation kit. Determined reads were demultiplexed, quality trimmed, and filtered using CLC Genomics Workbench 7.0 at a minimum variant read frequency of 2%.

For efficient recovery of plasmid DNA- and RNA-derived recombinant viruses, a strategy was designed to transfect DNA (plasmid) and RNA into cocultures of HEK293T and virus culturable (Vero, Vero-E6, and RD-A) cells. This strategy enhances the efficiency of recombinant virus production and growth in culturable cells. To validate the utility of this method, we proceeded with virus recovery under three culture conditions (293T plus Vero cells [HEK293T+Vero cells], Vero cells only, and HEK293T cells only) from DNA (plasmid) and RNA using the CV- B5 infectious clone and evaluated the titers of recombinant viruses produced. No virus was produced when only Vero cells were used, and very little virus detected with only HEK293T cells. Notably, however, recombinant viruses were successfully generated based on DNA/RNA transfection using HEK293T+Vero cell cocultures. Accordingly, recombinant viruses of all infectious clones were subsequently recovered using the coculture strategy (Fig. S5).

**FIG 5:**
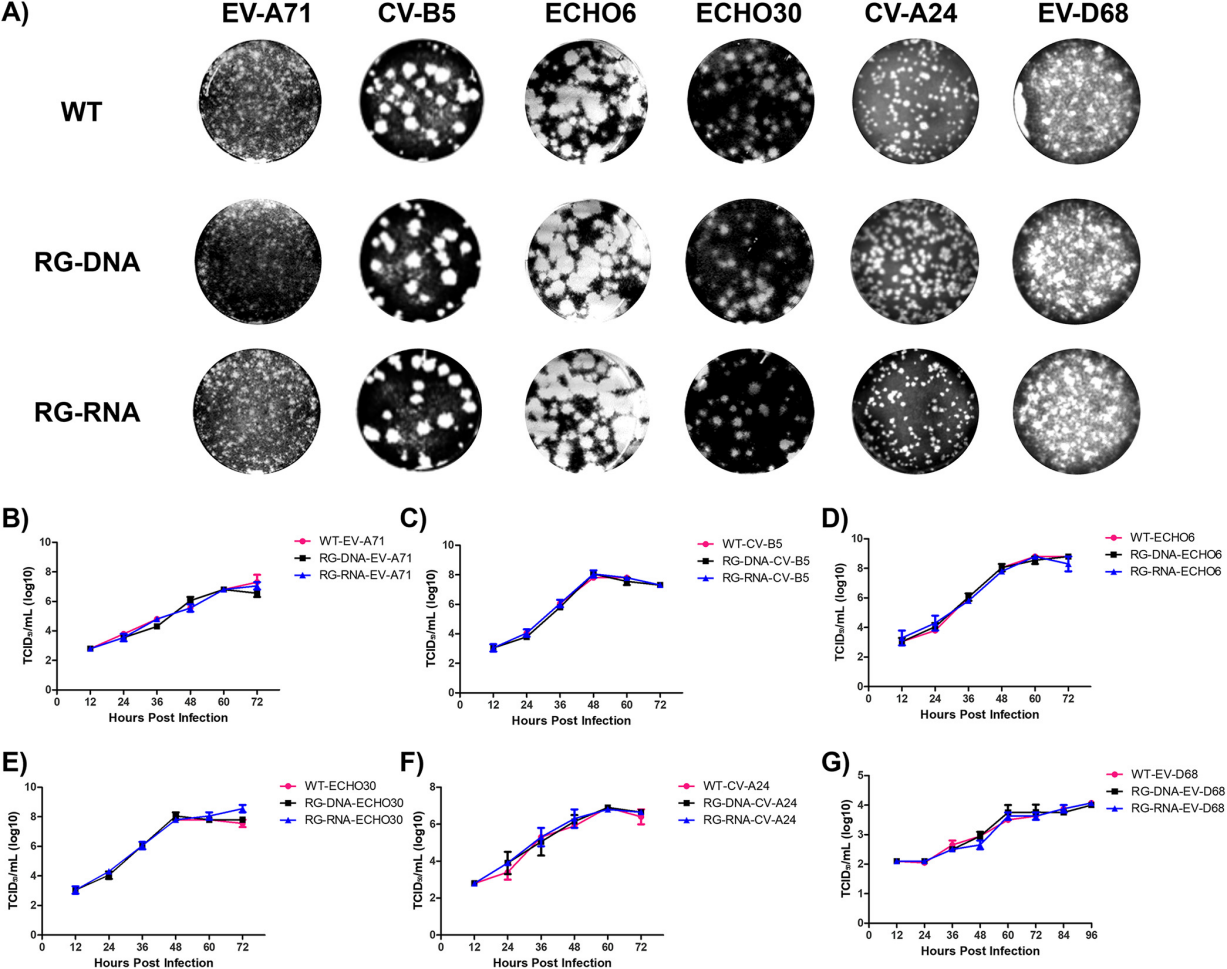
Characterization of the recovered viruses from enterovirus A to D infectious clones. (A) Plaque morphologies of recovered EV-A (EV-A71), EV-B (CV-B5, ECHO6, and ECHO30), EV-C (CV-A24), and EV-D (EV-D68) were compared to their respective parental strains (WT) 72 h postinfection. (B to G) Multistep growth curves of WT viruses and generated recombinant viruses grown in cells. Error bars represent standard deviations of the means from three independent experiments.

To verify the replication competency of infectious clones, plasmid DNA- and RNA-derived recombinant viruses were recovered. Their replicative properties were characterized and compared to those of the wild-type viruses. Clones of each serotype were transfected into appropriate cells with RNA (T7 *in vitro* transcripts) or DNA (plasmid). EV recombinant viruses EV-A71, CV-B5, ECHO6, ECHO30, CV-A24, and EV-D68 were successfully recovered from cells transfected with both RNA and DNA forms (Fig. 5 and Table 3). Furthermore, plaque assays were performed to confirm differences in viral characteristics between the WT virus and the recombinant virus generated from viral DNA/RNA forms for each EV serotype. *In vitro* replication kinetics were then assessed. All recombinant viruses showed plaque morphologies comparable to those of their respective WT viruses (Fig. 5A). Stock viral titers of WTs and recombinant EV-A71 (from 2.9 ×10^6^ to 3.7 × 10^6^ PFU/mL), CV-B5 (from 2.2 × 10^7^ to 3.2 × 10^7^ PFU/mL), ECHO6 (from 9.7 × 10^7^ to 1.1 × 10^8^ PFU/mL), ECHO30 (from 4.3 × 10^6^ to 4.8 × 10^6^ PFU/mL), CV-A24 (from 2.9 × 10^6^ to 3.7 × 10^6^ PFU/mL), and EV-D68 (from 6.8 × 10^3^ to 7.3 × 10^3^ PFU/mL) were comparable with each other (Table 3). To further determine the replication kinetics of all clone-derived EV viruses compared to the WT, cells were infected with recombinant EVs at a multiplicity of infection (MOI) of 0.001 and harvested every 12 h postinfection (hpi) for viral titration. All recombinant EVs, regardless of whether they were RNA or plasmid DNA derived, exhibited replication kinetics similar to those of their respective WTs. The WT and clone-derived EV-A71 showed peak viral titers of 7.3 log_10_ 50% tissue culture infective doses (TCID_50_)/mL and 6.8 log_10_ TCID_50_/mL for plasmid DNA-derived EV and 7.05 log_10_ TCID_50_/mL for RNA-derived EV, respectively (Fig. 5B). Furthermore, replications of recombinant CV-B5 clones and their WT were similar, with peak viral titers of 8.05 log_10_ TCID_50_/mL and 7.8 log_10_ TCID_50_/mL at 48 hpi, respectively (Fig. 5C). Plasmid DNA- and RNA-derived ECHO6 clones achieved their highest peaks of 8.8 log_10_ TCID_50_/mL at 60 and 72 hpi, respectively, compared to their WT (Fig. 5D). ECHO30 clones exhibited slightly higher peak viral titers (plasmid DNA clone, 8.05 log_10_ TCID_50_/mL; RNA clone, 8.55 log_10_ TCID_50_/mL) than their WT (7.8 log_10_ TCID_50_/mL) at 48 to 72 hpi (Fig. 5E). CV-A24 (plasmid DNA clone, 7.00 log_10_ TCID_50_/mL; RNA clone, 6.80 log_10_ TCID_50_/mL; WT virus, 7.00 log_10_ TCID_50_/mL) and EV-D68 (plasmid DNA clone, 4.15 log_10_TCID_50_/mL; RNA clone, 4.00 log_10_TCID_50_/mL; WT virus, 4.15 log_10_TCID_50_/mL) reached their peak viral titers at 60 and 96 hpi, respectively (Fig. 5F and G). Overall, the replication kinetics of both plasmid DNA- and RNA-derived recombinant viruses showed no significant difference from those of the WT at each time point. These results suggest that these recombinant viruses generated via RNA or plasmid DNA from the infectious clone do not significantly differ in phenotypic traits from their WT viruses. To evaluate the stability of the rescued virus, recovered CV-B5 was serially passaged 7 times in Vero cells and whole-genome sequencing was performed. No mutations were detected during 6 passages, while an R700K substitution was identified in VP1 at passage 7 (data not shown). R700K is potentially an adaptive mutation generated by viral cell adaptation, and the stability of the RG virus is considered high.

**TABLE 3:** Phenotypic characterization of EV infectious clones

Type	Infectivity, mean ± SD (PFU/mL)^*a*^					
	EV-A71 (×10^6^)	CV-B5 (×10^7^)	ECHO6 (×10^8^)	ECHO30 (×10^6^)	CV-A24 (×10^6^)	EV-D68 (×10^3^)
WT	3.498 ± 0.1	2.243 ± 0.3	1.119 ± 0.2	4.854 ± 0.6	2.123 ± 0.2	7.358 ± 0.1
RG-RNA	3.774 ± 0.6	2.416 ± 0.2	1.087 ± 0.1	4.496 ± 0.2	2.012 ± 0.6	7.123 ± 0.3
RG-DNA	2.925 ± 0.7	3.237 ± 0.1	0.989 ± 0.3	4.561 ± 0.5	2.328 ± 0.3	6.893 ± 0.5

a Data are presented as mean values of three biological replicates.

## DISCUSSION

Many EV infectious clones have been successfully developed in previous studies using various methods. However, the method in most cases is limited in that it is specific to each EV serotype. Furthermore, one of the problems identified, especially for emerging viruses without complete sequences, is the difficulty of applying the cloning method. We have described the design, construction, and application of our universal cloning method for the RG system of most human EVs (EV-A, -B, -C, and -D) as rapid, simple, time efficient, and cost-effective.

The construction of a full-length cDNA library is an indispensable step in RG. Usually, reverse transcription steps of RT-PCR and RACE-PCR for cloning are carried out separately, requiring two separate RT processes. However, this study devised a strategy that could allow cloning and RACE-PCR to proceed simultaneously using a single RT process, thus simplifying the cloning steps. We further described the application of reverse transcription with the TS method for cDNA construction with an end product that could be utilized simultaneously for RT-PCR and RACE-PCR. Because of its ability to bind to any part of RNA and CCC at the 5′ end, the specifically designed TS primer minimizes the nonspecific and rich background amplification generated during the PCR process. The redesigned TS method simplifies the cloning process without requiring additional enzyme except for the purification step. We thus applied and verified the devised strategy to generate whole-genome cDNAs of EV-A71, CV-B5, ECHO6, ECHO30, CV-A24, and EV-D68. Using the RT-TS products, we generated PCR products: viral whole-genome and 5’ UTR/3’ UTR RACE PCR segments. 3′ UTR RACE-PCR efficiency of CV-A24 appears to be lower than that of other viruses (Fig. 4B). The RP-3 primer (universal enterovirus 3′ UTR RACE-PCR-F) contains wobble nucleotides and is designed to target the conserved 2C region of EV-A to -D types. CV-A24 used in this study had four different nucleotides from the designed primer (Fig. S4). Analysis of sequence variations of type C EVs (*n* = 804) relative to the primer binding site revealed a total of 5 (1%) viruses with 2 to 4 nucleotide differences, with the majority containing the same sequence or a single nucleotide difference. Although the CV-A24 strain used in this study displayed the maximum number of four differences at the primer binding site, PCR and Sanger sequencing were possible. PCR amplification efficiency may therefore be enhanced using EV RNA with greater sequence homogeneity to the target site.

Several studies have focused on amplifying the full-length EV genome. For example, Lindberg et al. (27) amplified the full genomes of coxsackieviruses (B2, B5, and B6) and echoviruses (E1, E2, E3, E5, E7, E9, E15, E18, E21, E25, and E30) and generated infectious clones using sequence-dependent cloning methods. Tan et al. (28) amplified three fragments of the genome of EV71 for deep sequencing and analysis of the full viral genome. However, the primers designed for use in both studies showed limitations in terms of being applicable only to specific strains. The universal and versatile primers designed in the current study not only facilitated amplification of the full genomes of most human-infecting EVs but also could be used to generate infectious clones. In another earlier study, Isaacs et al. (29) designed a universal primer for amplifying the near-full genomes of EVs, including whole-genome analysis of clinical samples via the deep sequencing method. However, the amplicon did not include the EV 5′ and 3′ ends, precluding genuine full-genome sequencing and infectious clone generation.

Generally, the EV replication mechanism utilizes mRNA complexes for cap-independent translation of the viral RNA. The 7-methylguanosine (m^7^G)-capped region plays a role in nuclear RNA export for virus production, especially among positive-stranded RNA genomes that lack terminal cap structures (21, 30). Once exported to the cytosol, viral RNA is processed for internal ribosome entry site (IRES)-dependent translation (21). The designed construct enhanced EV genomic RNA transcription by cellular RNA polymerase II. Although CMV promoters can yield genomic RNA with an m^7^G-capped 5′ end of the transcript, *in vitro*-transcribed RNA is directly transfected into the cytosol for IRES-dependent translation. Furthermore, the autocatalytic ribozymes Hh-Rbz and HDV-Rbz were added to generate precise 5′ and 3′ ends of the transcribed viral RNA. Hh-Rbz removes unnecessary residues left after T7 transcription at the 5′ end of the viral RNA, while HDV-Rbz cleaves the SV40 termination signal at the 3′ end of the viral RNA. Nonetheless, both ribozymes can improve the cleavage of nonviral nucleotides to enhance the infectivity of infectious clones (21, 30, 31).

We thus reconstructed an expression vector with dual transcriptional control of CMV and T7 immediate early promoters and the SV40 termination signal for generation of infectious clones of various human EV species and further use in research on genetic vaccines and gene therapy. Using the infectious clone of RNA or DNA, we produced recombinant viruses with phenotypic characteristics similar to those of their parental strains. Virus yield and plaque morphology of the two forms were comparable. Moreover, the successful transfection of infectious clones supports the capability of the dual-promoter system to export to the cytosol and transcribe m^7^G-capped and uncapped forms for IRES-dependent translation and complex formation (21). The dual construct will contribute to studies requiring DNA- or RNA-based infectious genomes without needing additional modification of the RG system.

**FIG 6:**
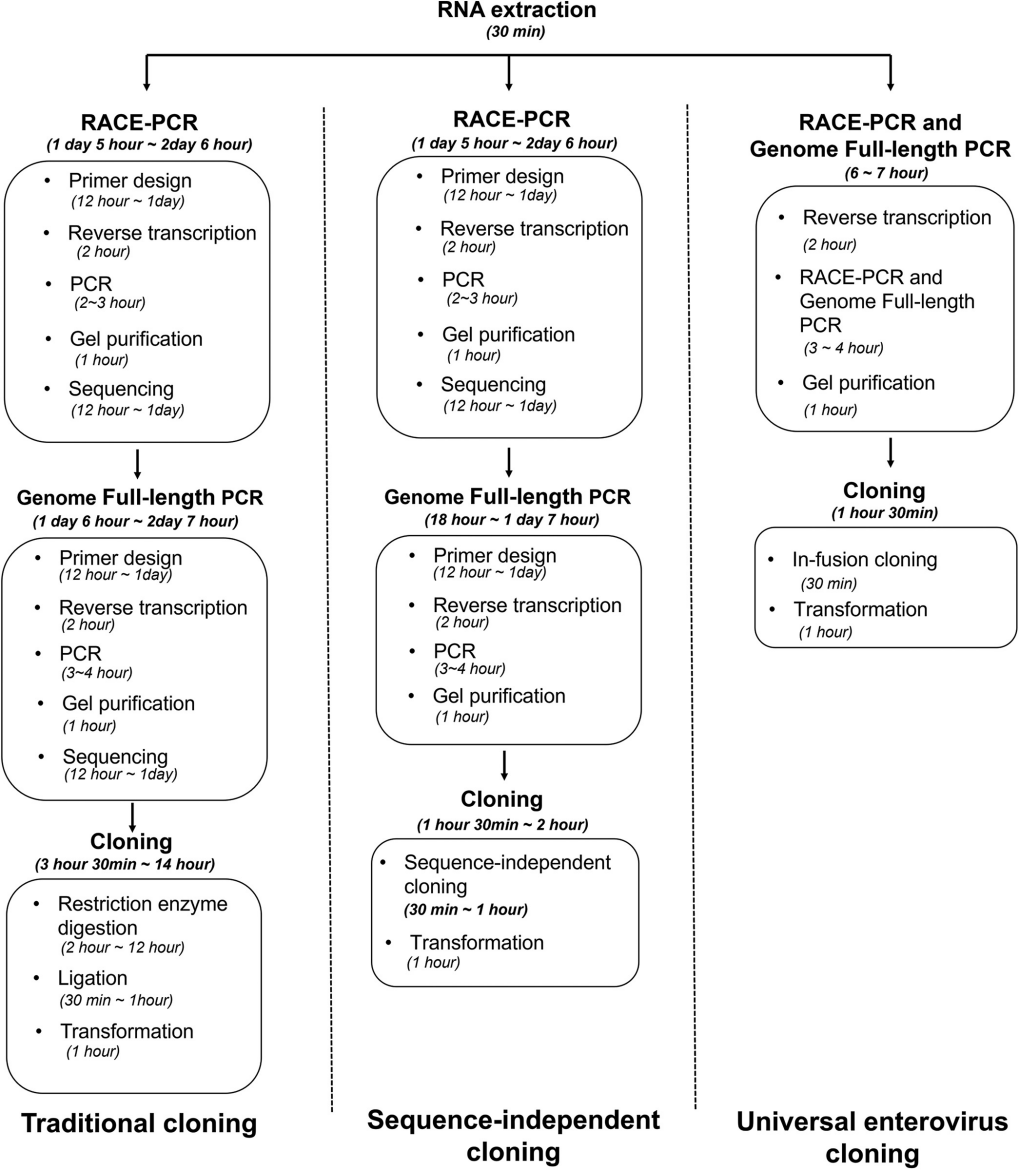
Schematic diagram of universal enterovirus cloning and comparison with the other cloning methods. The diagram shows the simplification and convenience of the universal enterovirus cloning when the devised strategies are applied compared to other cloning methods.

Moreover, the simplification and convenience of the cloning step can significantly impact future research on human EVs and other emerging EVs. We generated a sequence-independent system and compared it with other cloning strategies devised for other EV serotypes (Fig. 6). After detection and identification of the EV, clones can be generated within a day without prior whole-genome sequencing through in-fusion cloning of the produced universal cloning vector or EV whole-genome RT-PCR method. In comparison, traditional or conventional cloning for RNA virus infectious clones needs steps such as amplification of cDNA ends (RACE-PCR), sequencing, and RT-PCR with designed primers to amplify the whole genome of the virus (32). The amplified whole genome is then fully sequenced with selection of appropriate enzymes based on multiple-cloning sites of the vector. The primers may need to be redesigned to include enzyme site sequences. The amplified inserts are cloned into the vector using restriction enzyme digestion and ligation. If the vector is designed with a limited enzyme site, it could affect the cloning efficiency, mainly when amplified inserts are digested into several pieces (30, 32). In addition, cloning efficiency can vary depending on the temperature and time at which the insert is subjected to restriction and ligation (25). The enzyme digestion and ligation process scope are limited as enzymes vary depending on the cloned genomic sequence (23).

Our universal cloning strategy for human EVs has potential in direct amplification of the full-length genome from clinical isolates. Estimation of the minimum amount of viral RNA for amplification of the full-length genome using our method revealed the requirement for at least 4.8 log_10_ viral RNA copies or 2.8 log_10_ TCID_50_/mL for effective use as insert DNA (Fig. S3). Previous studies have reported the presence of viral particles in the range of 3.0 to 7.0 log_10_ RNA copies/mL or 1.8 to 3.5 log_10_ TCID_50_/mL in EV clinical samples (cerebrospinal fluid [CSF], stool and blood) (33–35). While further research on this issue is warranted, direct cloning of clinical samples may be successfully achieved in cases where sufficient viral RNA is available.

Infectious clones of enterovirus produced using our strategy can be applied in various research fields. A recombinant virus containing a reporter gene (expressing enhanced green fluorescent protein [eGFP]) was successfully generated using the CV-B5 infectious clone in this study (Fig. S6). The recombinant eGFP-expressing virus will be useful for drug screening and viral characterization analyses, supporting the theory that the availability of a rapid and simple enterovirus cloning system should accelerate discoveries in enterovirus research.

Overall, our universal EV cloning methodology simplifies these tedious processes of conventional cloning and minimizes the cost of production without to compromising its efficiency in generating recombinant clones. With the development of infectious clone technology, manipulating RNA viruses at the molecular level will allow us to understand RNA viral genomic structure and functions. With continuous emergence of new EVs, this cloning technology for infectious EV clones would significantly contribute to the rapid characterization of emerging EV species. Finally, this methodology can also contribute to future development of genetic/viral vector vaccine platforms.

## MATERIALS AND METHODS

### Reagents and biological components.

Cell lines used in this study included rhabdomyosarcoma (RD) cells, human embryonic kidney 293T (HEK293T) cells, Vero cells from the Korean Cell Line Bank (KCLB; Seoul, Republic of Korea), and Vero E6 cells (CRL1586; ATCC) cells. All cells were maintained with Dulbecco’s modified Eagle’s medium (DMEM; Gibco-Invitrogen, Carlsbad, CA, USA) supplemented with 10% (vol/vol) heat-inactivated fetal bovine serum (FBS) and 1% antibiotics (Gibco-Invitrogen) at 37°C with 5% CO_2_. EV-A71 isolate 2018/CSF/KOR, CV-B5 isolate 2018/CSF/KOR, ECHO6 isolate 2018/CSF/KOR, and ECHO30 isolate 2018/CSF/KOR were obtained from cerebrospinal fluid (CSF) samples collected at the Chungbuk National University Hospital (institutional review board [IRB] no. CBNU-201809-BR-720-01). CV-A24 (KBPV-VR-12) was acquired from the Korean Bank for Pathogenic Viruses (Republic of Korea), and EV-D68 (ATCC VR-1823) was purchased from the ATCC. EV-A71 was propagated in Vero E6 cells. CV-B5 was propagated in Vero cells. ECHO6, ECHO30, CV-A24, and EV-D68 (33°C, 5% CO_2_) were grown in RD cells. After 48 to 72 h, cell suspensions were harvested and processed for three consecutive freeze-thaw cycles prior to storage at –80°C or they were used immediately. All cell culture reagents were purchased from Gibco (Grand Island, NY, USA). The transfection reagent (JetMESSENGER kit and jetPEI) was purchased from Polyplus Transfection (New York, NY, USA). Restriction enzymes (DpnI and AgeI) were purchased from New England BioLabs (NEB; Ipswich, MA, USA). Other products, such as deoxynucleoside triphosphates (dNTPs) and DH5α chemically competent cells, were purchased from Enzynomics, Inc. (Daejeon, Republic of Korea). Oligonucleotides such as primers and nucleotide fragments were obtained from Bionics, Inc. (Daejeon, Republic of Korea).

### Primer design.

For designing primers, 2,349 whole-genome sequences from the 5′ UTR to the 3′ UTR of EV-A (*n* = 737), EV-B (*n* = 260), EV-C (*n* = 804), and EV-D (*n* = 548) were downloaded from the Virus Pathogen Database and Analysis Resource (ViPR) research database site (https://www.viprbrc.org). These sequences were aligned using CLC Genomics Workbench 7.0 (Qiagen, Carlsbad, CA, USA). Conserved regions in the targets, 5′ UTR, 3′ UTR, and 2C genes, were used as templates for the design of primer sets (Fig. 3 and 4 and Table 1).

### cDNA synthesis.

EV genomic RNA was extracted using a QIAmp viral RNA minikit (Qiagen, Valencia, CA, USA) following the manufacturer’s instructions to generate cDNA for whole-genome PCR. Extracted viral RNA was synthesized using 50 pmol of the reverse transcription primer (CS-1) at 65°C for 5 min, followed by 2 min of incubation on ice prior to the addition of Superscript II reverse transcriptase (Invitrogen, Carlsbad, CA, USA) (Table 1). The mixture was then incubated at 42°C for 30 min, followed by the addition of 30 pmol of template-switching (TS) primer (CS-2) designed with TTAAAA nucleotide extensions for EV RNA specificity (Table 1). The reaction was inactivated at 72°C for 15 min.

### Dual-promoter vector design for RG system.

To construct a vector plasmid enabling infectious virus recovery (empty vector, named pUC19-CMV/T7), a CMV/T7 promoter and the simian virus 40 (SV40) polyadenylation signal were located upstream of the EV 5′ UTR and downstream of the EV poly(A) tail, respectively. Synthesized nucleotide fragments of the self-cleaving ribozyme sequences such as hammerhead (Hh) and hepatitis delta virus (HDV) were inserted between the CMV/T7 promoter system and at the poly(A)_50_ tail ends and SV40, respectively (Fig. 1A). Synthesized and amplified promoter constructs were inserted into the pUC19 plasmid using primers CV-1 and CV-2 (Table 1). Vector expression was performed using primers CV-3 and CV-4 (Table 1). The sequence was amplified with 2 units of Phusion high-fidelity DNA polymerase (NEB, Ipswich, MA, USA) following the manufacturer’s protocol. Amplification conditions were as follows: an initial denaturation step at 98°C for 30 s and then 35 cycles of 98°C for 10 s, 60°C for 30 s, and 72°C for 2 min, followed by a final elongation step at 72°C for 7 min. Purified products were then treated with DpnI (NEB) to digest the pUC19 plasmid template. The insert and linearized vector were infused using the TaKaRa In-Fusion cloning kit (TaKaRa Bio Inc., Kusatsu, Shiga, Japan) according to the manufacturer’s instructions. After transformation into competent cells, positive clones of 1 kb were selected by colony PCR using primers M13-F and M13-R (Table 1). Selected clones were subjected to sequencing for confirmation. The constructed expression vector, pUC19-CMV/T7, was treated with AgeI enzyme (NEB, Ipswich, MA, USA) to prevent back transformation during the cloning process.

### Enterovirus cloning into the pUC19-CMV/T7 vector.

The linearized universal cloning vector was prepared by PCR amplification of the pUC19-CMV/T7 plasmid template using the universal cloning vector primers UC-1 and UC-2 extended to the EV 5′ and 3′ ends, respectively (Table 1). The PCR was performed using a reaction mixture of 2 μL of Phusion high-fidelity DNA polymerase (NEB, Ipswich, MA, USA), 20 μL of 5× HF buffer, 10 μL of 2 mM dNTP, 2 μL of 50 mM MgCl_2_, 10 μL of 5-pmol primers, and 100 ng of pUC19-CMV/T7 plasmid, with distilled water (DW) added to achieve a total reaction volume of 100 μL. The reaction was performed with an initial denaturation step at 98°C for 30 s and then 35 cycles of 98°C for 10 s, 65°C for 30 s, and 72°C for 2 min, followed by a final elongation step at 72°C for 7 min. The PCR product was treated with 2 μL of DpnI (Enzynomics, Republic of Korea) for 2 to 12 h to remove the template plasmid used for PCR. DpnI-digested PCR amplicons were purified using a QIAquick gel extraction kit (Qiagen, Valencia, CA, USA) following the manufacturer’s instructions (concentrations above 25 ng/μL). To prepare EV whole-genome cDNA as an insert for cloning, six serotypes of synthesized EV cDNA were amplified using universal insert primers UC-3 and UC-4. Primer sequences of the flexible region for each serotype (listed in Table S1) possessed conserved regions identified for each EV species (Table 1). PCRs were performed using a reaction mixture of Phusion high-fidelity DNA polymerase (NEB), 20 μL of 5× HF buffer, 10 μL of 2 mM dNTP, 2 μL of 50 mM MgCl_2_, 10 μL of a 5-pmol concentration of each cloning insert primer mentioned, and 5 μL of cDNA, with DW added to achieve a total reaction volume of 100 μL. The mixture was then processed at 98°C for 30 s for initial denaturation, followed by 35 cycles of 98°C for 10 s, 68°C for 30 s, and 72°C for 3.5 min and then a final elongation step of 72°C for 7 min. Insert PCR products were also purified using a QIAquick gel extraction kit (Qiagen) according to the manufacturer’s instructions (concentrations above 25 ng/μL).

To infuse the prepared vector and insert, a mixture of each 100-ng insert (EV whole-genome segment) and linearized vector, 2 μL of In-Fusion cloning enzyme (TaKaRa Bio Inc., Kusatsu, Shiga, Japan), and DW adjusted to a total volume of 10 μL was prepared and incubated at 50°C for 15 min. The reaction was then chilled for 10 min on ice and processed for transformation using competent cells (Enzynomics, Inc., Daejeon, Republic of Korea). Several random independent colonies were picked and collected for single colony PCR using the forward primer of universal enterovirus 3′ UTR RACE-PCR (RP-1) and reverse primer of the pUC19-CMV/T7 insert (CV-4) (Table 1). Clones showing bands of the expected size were seeded in LB broth containing ampicillin (50 μg/mL). Plasmids were harvested and purified using a plasmid DNA purification kit (Bionics Inc., Daejeon, Republic of Korea) according to the manufacturer’s protocol. Plasmid sequencing was carried out using next-generation sequencing (NGS).

### RACE-PCR.

To perform rapid amplification of cDNA ends (RACE)-PCR, 5 μL of the primer sets of 5′ UTR (RP-1 and RP-2) or 3′ UTR (RP-3 and RP-4) (Table 1) was mixed with Phusion high-fidelity DNA polymerase (NEB, Ipswich, MA, USA). The same reaction also included a mixture of 10 μL of 5× GC buffer, 5 μL of 2 mM dNTP, 1 μL of 50 mM MgCl_2_, 3 μL of cDNA, 1.5 μL of dimethyl sulfoxide (DMSO), and DW, with the total reaction volume being 50 μL. The reaction was then carried out with an initial denaturation step at 98°C for 30 s and then 35 cycles of 98°C for 10 s, 62°C for 30 s, and 72°C for 20 s, followed by a final elongation step at 72°C for 7 min. PCR products were purified using a QIAquick gel extraction kit (Qiagen, Valencia, CA, USA) according to the manufacturer’s instructions. Each PCR product was then subjected to Sanger sequencing (Bionics, Republic of Korea) using specifically designed primers: enterovirus A to D 5′ UTR RACE-PCR Sanger primer (RP-5) with enterovirus A 3′ UTR RACE-PCR Sanger primer (RP-6 to -9).

### Deep sequencing of enterovirus infectious clones.

For deep sequencing of each EV infectious clone, a library was prepared using the Nextera XT DNA library preparation kit (Illumina, San Diego, CA, USA). Libraries were sequenced using an Illumina MiSeq system. Determined reads were then demultiplexed, quality trimmed, and filtered using CLC Genomics Workbench 7.0 (Qiagen, USA). Reads were aligned and mapped to wild-type virus references and pUC19 vector sequences. Results were derived using a quality-based variant detection pipeline. The minimum variant read frequency was set at 2%, with the criterion of variant supported with a minimum of 10 reads.

### Enterovirus recovery from infectious clones.

To verify that the recombinant virus of an infectious clone of RNA form could be recovered, RNA was produced using a T7 MegaScript kit (Invitrogen, Carlsbad, CA, USA) according to the manufacturer’s protocol. The concentration of *in vitro*-transcribed RNA was determined using a Qubit RNA High Sensitivity assay kit (Invitrogen). Using a JetMESSENGER kit (Polyplus Transfection, New York, NY, USA), 5 μg of the prepared RNA was transfected into cells placed into 6-well plates following the manufacturer’s protocol. To verify the possibility of directly recovering the recombinant virus from DNA plasmid-generated infectious clones, 3 μg of the DNA plasmid-based infectious clone was transfected into cells in a 6-well plate using the jetPEI transfection reagent (Polyplus Transfection). Each plate was prepared as follows: HEK293T+Vero E6 cells for EV-A71 clones, HEK293T+Vero cells for CV-B5 clones, and HEK293T+RD cells for ECHO6, ECHO30, CV-A24, and EV-D68. At 18 h posttransfection, each transfection supernatant was replaced with 2 mL of 2% DMEM and the cytopathic effect was monitored for the next 96 h. All recombinant viruses produced were titrated using TCID_50_ and plaque assays.

### Plaque assay.

To determine plaque formation and stock viral concentrations of recombinant viruses compared to those of their parental viruses, Vero, Vero E6, and RD cells were seeded into 6-well plates and 1 mL of 10-fold serially diluted recombinant/parental viruses was added to cells. These plates were incubated in a 37°C CO_2_ incubator for 2 h to allow the virus to attach to cells. A 0.7% DMEM-agarose overlay medium was prepared by mixing 1.4% agarose and 2× DMEM. Finally, the viral inoculum was removed and replaced with 3 mL of overlay medium for each well. The plate with the overlay was then cooled at 4°C for 10 min before incubation in a 37°C 5% CO_2_ incubator. Cells were fixed with 10% formaldehyde and stained with 1% (wt/vol) crystal violet at 96 h postinfection. Wells containing 20 to 100 plaques were selected and analyzed using ImageJ software (13) (National Institutes of Health, Bethesda, MD, USA). PFU were then calculated.

### *In vitro* growth kinetics.

For *in vitro* viral growth kinetics, the wild-type and recombinant EVs were diluted to a multiplicity of infection (MOI) of 0.001 and then used to infect Vero E6 (EV-A71), Vero (CV-B5), and RD (ECHO6, ECHO30, CV-A24, and EV-D68) cells. Plates were incubated in a 37°C 5% CO_2_ incubator for 2 h. After the viral adsorption period, the medium was replaced with DMEM containing 2% (vol/vol) FBS. Cell culture supernatants were harvested at 12, 24, 36, 48, 60, and 72 h postinfection. Viral titers at each time point were measured using the TCID_50_.

### Data availability.

Genome sequences of enteroviruses deposited in GenBank (accession numbers KP289394 to MW384881; *n* = 2,349) were used for primer design in this study (see Table S2 for details). Sequence information of enteroviruses, including EV-A71, CV-B5, ECHO6, and ECHO30, used in this study for the generation of infectious clones are deposited at NCBI with accession numbers OQ117372, OQ117373, OQ117371, and OQ117370, respectively. Genome alignment and analysis were conducted using CLC Genomics Workbench (CLC bio/Qiagen, Denmark).
