# Supplementary material for: Development of a Universal Cloning System for Reverse Genetics of Human Enteroviruses
Source: Microbiol Spectr. 2023 Jan 18;11(1):e03167-22. doi: 10.1128/spectrum.03167-22 (PMC9927166; doi:10.1128/spectrum.03167-22)
Supplement: Supplemental file 1 — Supplemental material. Download spectrum.03167-22-s0001.pdf, PDF file, 4.7 MB [file spectrum.03167-22-s0001.pdf]

Supplementary Figure 1.

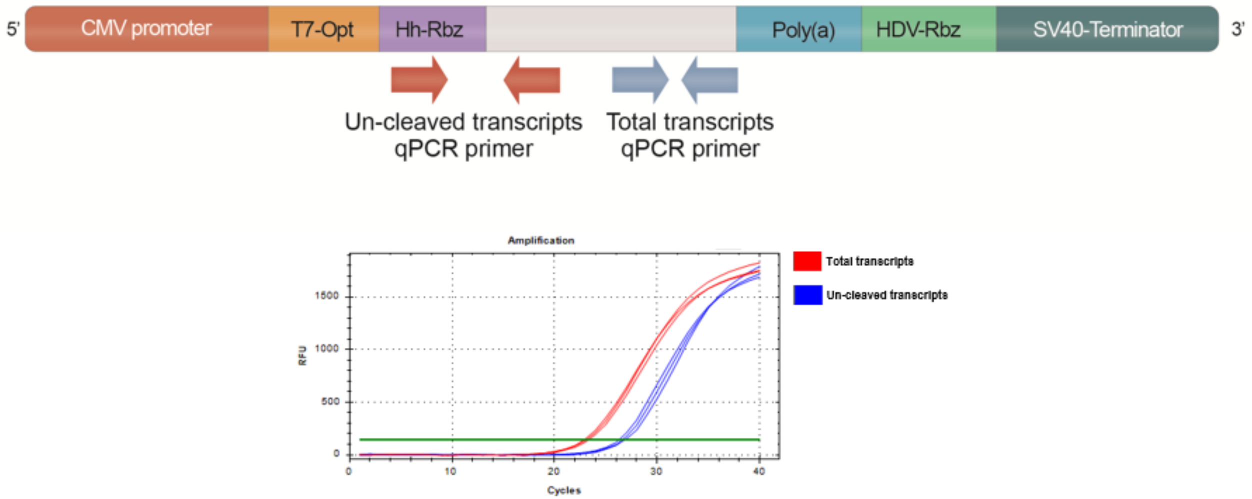

Number	Total transcripts		Un-cleaved transcript		Cleavage efficiency(%)
	Ct-value	RNA concentration	Ct-value	RNA concentration	
1	23.37	3.11×10^8	26.92	1.88×10^5	93.93
2	22.93	4.38×10^8	26.65	2.34×10^5	94.65
3	23.15	3.70×10^8	26.33	1.12×10^5	96.8

Supplementary Figure 1. Optimization of the Hammerhead ribozyme (Hh-RBz) for enhancing self-cleaving efficiency. A) Schematic illustration of the evaluation of HH-Rbz for its auto-cleaving efficiency and primers used for the evaluation. Arrows indicate targets of primers designed to confirm the cleavage of the RNA isolated from the transfected cells. B) Cleavage efficiency was measured and tested using a reverse quantitative PCR (qRT-PCR) assay. Vero cells were transfected with 5 μg of prepared RNA using a JetMESSENGER kit according to the manufacturer's instructions**.** At 18 h after transfection, cells were collected and lysed and one step RT-qPCR was performed**.** The method detected transcriptional gradient products: total transcript (red), which is lost upon cleavage due to lack of primer binding, and uncleaved transcript (blue) products, which are always detected regardless of Hh-Rbz cleavage. The percent cleavage efficiency was determined using the following formula: ((total transcripts – uncleaved transcripts) / total transcripts) X 100).

Supplementary Figure 2.

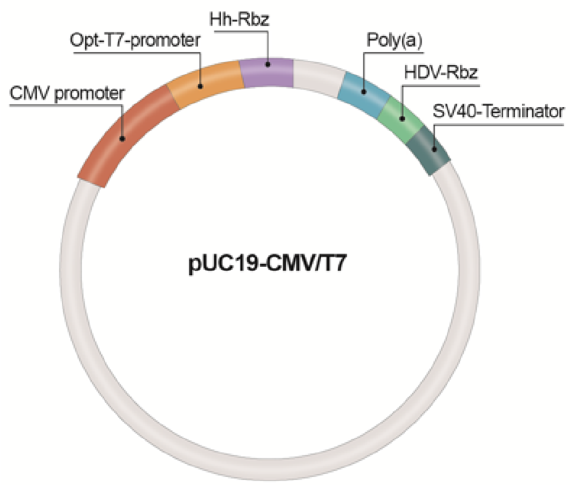

pUC19-CMV/T7 construct component sequence

Construct Component*	Sequence (5' to 3')	Bp
pcDNA 3.1 vector CMV promoter Sequence	CGTTACATAACTTACGGTAAATGGCCCGCCTGGCTGACCGCCCAACG ACCCCCGCCCATTGACGTCAATAATGACGTATGTTCCCATAGTAACG CCAATAGGGACTTTCCATTGACGTCAATGGGTGGAGTATTTACGGTAA ACTGCCCACTTGGCAGTACATCAAGTGTATCATATGCCAAGTACGCC CCCTATTGACGTCAATGACGGTAAATGGCCCGCCTGGCATTATGCCC AGTACATGACCTTATGGGACTTTCCTACTTGGCAGTACATCTACGTAT TAGTCATCGCTATTACCATGGTGATGCGGTTTTGGCAGTACATCAATG GGCGTGGATAGCGGTTTGACTCACGGGGATTTCCAAGTCTCCACCCC ATTGACGTCAATGGGAGTTTGTTTTGGCACCAAAATCAACGGGACTTT CCAAAATGTCGTAACAACTCCGCCCCATTGACGCAAATGGGCGGTAG GCGTGTACGGTGGGAGGTCTATATAAGCAGAGCTCGTTTAGTGAACC GT	523
T7 Promoter Optimization Sequence	TAATACGACTCACTATAGGGAGA	22
Entero 5'UTR Hh-Rbz Sequence	TTTAACTGATGAGTCCGTGAGGACGAAACGGAGTCTAGACTCCGTC	46
Insert+ Poly(A) Tail[A]	GCTACCATCTA**ACCGG**TTCATTCTCA*AAAAAAAAAAAAAAAAAAAAAAA AAAAAAAAAAAAAAAAAAAAAAAAAAA*	76
HDV-Rbz Sequence	GGCCGGCATGGTCCCAGCCTCCTCGCTGGCGCCGGCTGGGCAACAT TCCGAGGGGACCGTCCCCTCGGTAATGGCGAATGGGACCTAGCATA ACCCCTTGGGGCCTCTAAACGGGTCTTGAGGGGTTTTTTG	132
pcDNA 3.1 vector SV40 terminator Sequence	AACTTGTTTATTGCAGCTTATAATGGTTACAAATAAAGCAATAGCATCA CAAATTTCACAAATAAAGCATTTTTTTCACTGCATTCTAGTTGTGGTTT GTCCAAACTCATCAATGTATCTTATCATGT	128

[A]The component sequence includes the AgeI restriction site (bold) followed by the poly$(A)_{50}$ tail of the antigenome.
*Abbreviations: CMV: Cytomegalovirus; Hh-RBz: Hammerhead ribozyme; HDV-RBz: Hepaptitis delta virus; SV40: Simi an Virus 40 terminator;

Supplementary Figure 2. Construct and sequence of cloning vector (pUC19-CMV/T7) for *Enterovirus* infectious clone generation. Scheme of the component construct and sequences added to the pUC19 vector for making *Enterovirus* (EV) cloning vector. The vector has two types of promoters (CMV and T7) for viral RNA transcription and two types of ribozymes (Hh-Rbz and HDV-Rbz) to produce the desired viral RNA. Downstream of HDV-Rbz sequence has SV40-terminator to stop the transcription. Component sequences according to the vector construct are listed in the table.

Supplementary Figure 3.

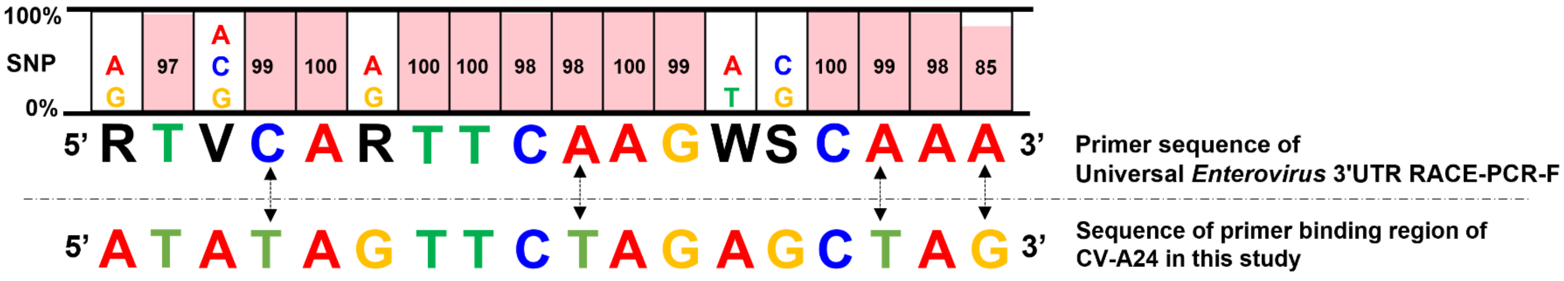

No. of nucleotide differences in the primer binding region	Number of corresponding sequences / Total number of type C EV sequences (%)
0	649/804 (80.7%)
1	147/804 (18.3%)
2	2/804 (0.5%)
3	1/804 (0.1%)
4	3/804 (0.4%)

Supplementary Figure 3. Sequence homology and heterogeneity distribution between Universal Enterovirus 3'UTR RACE-PCR-F (RP-3) primer and type C Enterovirus. Universal Enterovirus 3'UTR RACE-PCR-F Primer designed with Wobble + conserved nucleotide sequences. The consensus sequences of the primer and its binding region among type C EVs (N=804), as determined via single nucleotide polymorphism (SNP) analysis, are shown above the dotted line. The single nucleotide differences between the primer and primer binding region of CV-A24 used in this study are shown below the dotted line. The table presents the number and distribution rate of type C EVs by the number of sequence differences compared to the primer.

Supplementary Figure 4.

A)

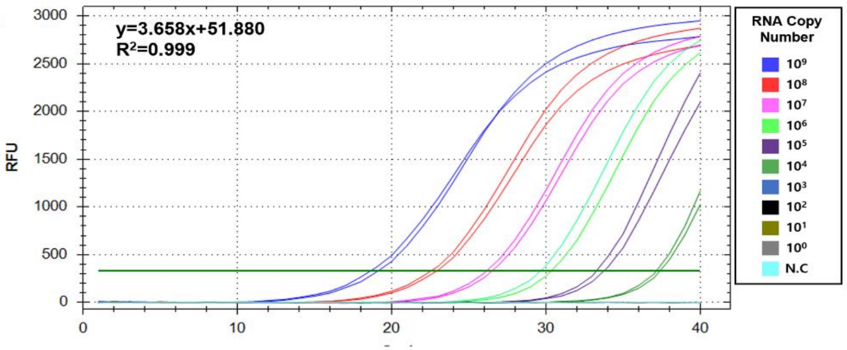

	RNA copy number							
	1×10^9	1×10^8	1×10^7	1×10^6	1×10^5	1×10^4	$1\times10^3 \sim 1\times10^0$	N.C
Mean CT value ± (SD)	18.86±0.33	22.725±0.27	26.315±0.24	29.975±0.48	33.5±0.33	37.265±0.16	-	-

In-vitro-transcribed RNA for standard curve

B)

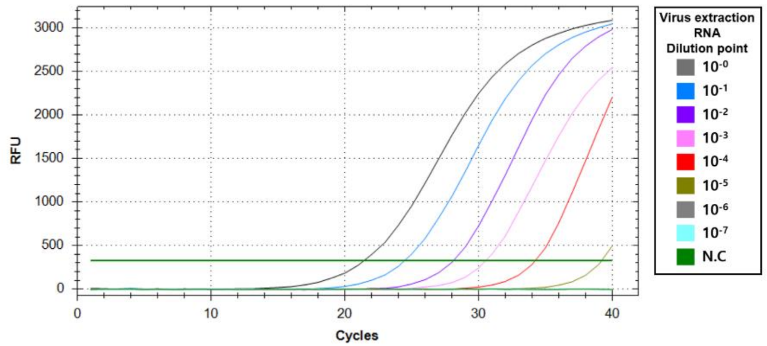

	Virus dilution point							
	$1x10^{-0}$	$1x10^{-1}$	$1x10^{-2}$	$1x10^{-3}$	$1x10^{-4}$	$1x10^{-5}$	$1x10^{-6}$	$1x10^{-7}$
CT value	21.44	24.47	28.13	30.54	34.24	39.21	-	-
Log_{10} RNA Copy number	8.3	7.4	6.4	5.8	4.8	3.4	-	-
Log_{10} $TCID_{50}$/mL	6.8	5.8	4.8	3.8	2.8	1.8	0.8	-

Virus extraction RNA

C)

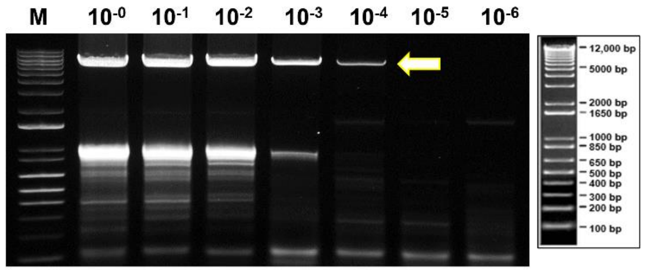

Full-length genome amplified by RT-PCR

D)

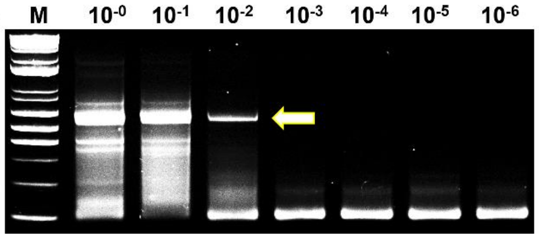

5'UTR RACE-PCR

E)

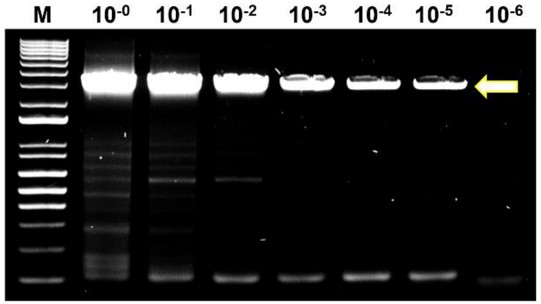

3'UTR RACE-PCR

Supplementary Figure 4. Determination of the minimum viral RNA copy number required for amplification of the full-length genome of enterovirus for universal cloning. A) Ten-fold serial dilutions (1×10^0 to 1×10^9) of *in vitro* RNA transcripts from Coxsackievirus B5 Infectious clone were prepared in duplicate for RT-qPCR standard curve (CV-B5 RNA) generation using custom-designed primers for the CV-B5 VP1 gene. Each dilution was labeled with a different color for identification. RT-qPCR-positive amplification was determined from the mean cycle threshold value for each RNA dilution point, as shown in the table. B) The RNA extract from cell-propagated CV-B5 was used to determine the number of viral RNA copies based on the standard curve. The viral RNA extract was ten-fold serially diluted (10^{-0} to 10^{-7}) and subjected to RT-qPCR to measure viral RNA copy number using the custom-designed CV-B5 VP1 detection primer. Different colors were used to label each dilution. The viral RNA copy number per dilution point was calculated based on the standard curve generated with RNA transcripts (shown in the table below). N.C: negative control; Ct: cycle threshold; SD: standard deviation; "-": not determined. Ten-fold serially diluted viral RNA (10^{-0} to 10^{-6}) extracted from CV-B5 was synthesized to generate whole genome CV-B5 cDNA. C) Gel electrophoresis analysis of the full-length genome amplified from each diluted cDNA. D) Gel electrophoresis analysis of the 5'UTR RACE-PCR product amplified from each diluted cDNA. E) Gel electrophoresis analysis of the 3'UTR RACE-PCR product amplified from each diluted cDNA. Arrows indicate target PCR products. M: 1 kb, DNA size ladder.

Supplementary Figure 5.

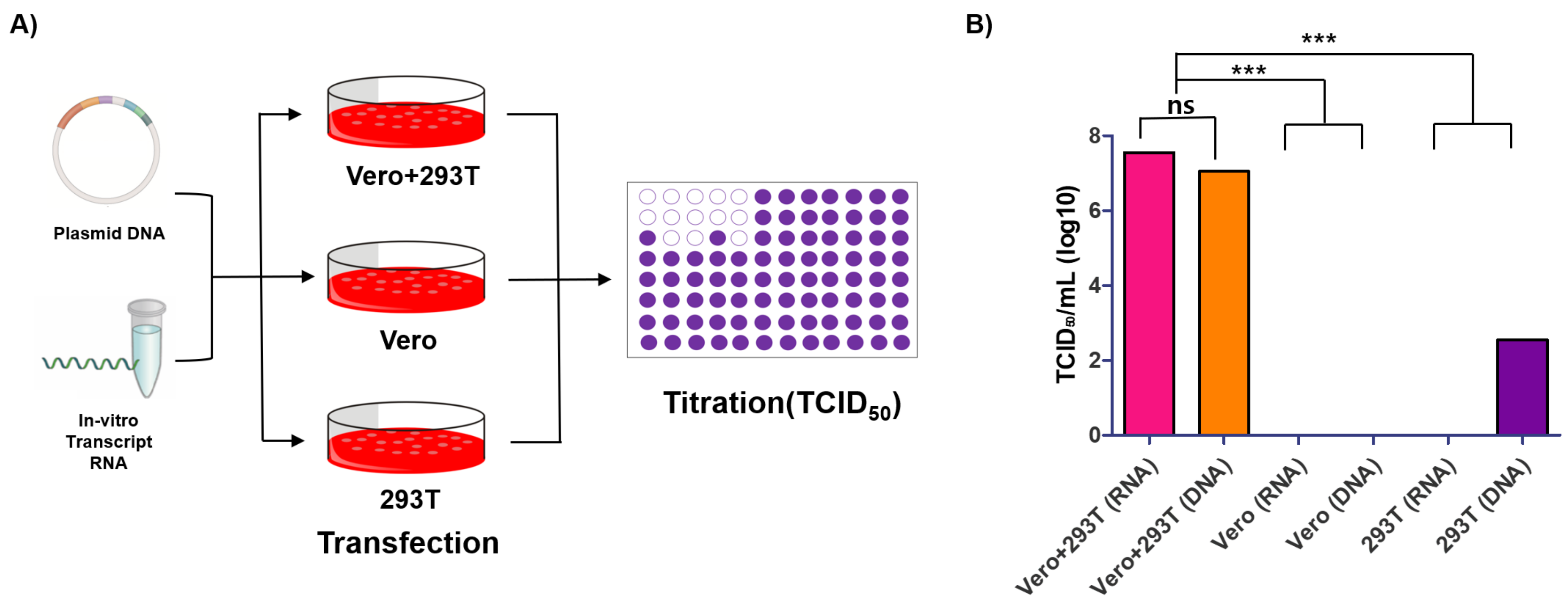

Supplementary Figure 5. Validation of co-culture-based transfection strategy for recombinant virus generation. A) The schematic diagram depicting the strategy validation of the virus generation. B) The viral recovery efficiency of the DNA- and RNA-derived recombinant virus was evaluated in three conditions (Vero+293T, Vero only, and 293T only) after DNA and RNA transfection. Asterisks indicate the p-values, ***P<0.0001 representing significantly different, and ns representing not significantly different among the compared groups.

Supplementary Figure 6.

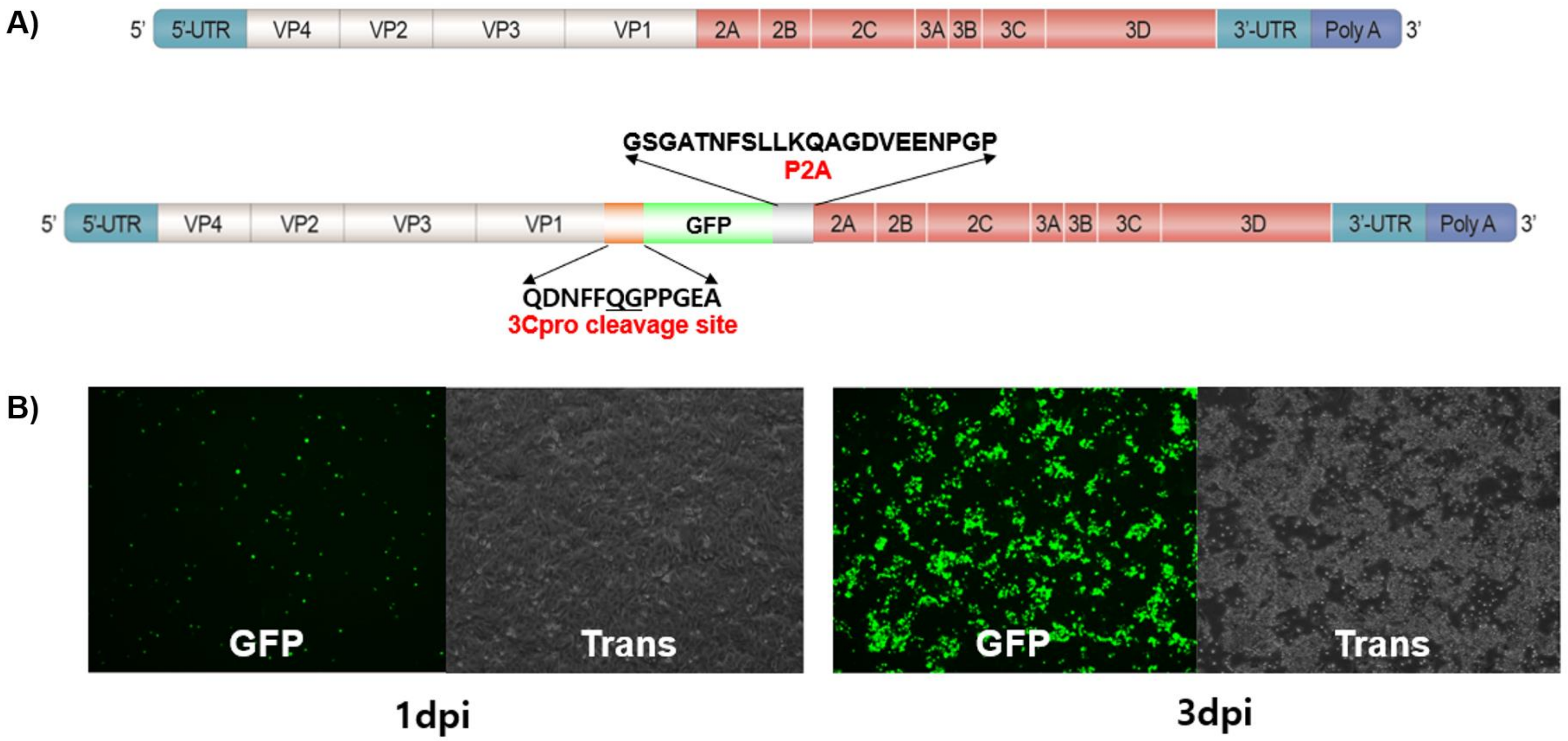

Supplementary Figure 6. Construction of eGFP expressing RG-CV-B5 virus and confirmation of eGFP expression. A) Schematic diagram illustrating the eGFP expression of the constructed RG-CV-B5 virus. The eGFP ORF gene was incorporated between VP1 and P2A of CV-B5, and 3C protease cleavage site and P2A amino acid were added to both sides for cleavage. B) Recovering the eGFP-expressing of the RG-CV-B5 virus and confirming the GFP expression on days 1 and 3 after infection in the Vero cell.

Supplementary Table 1: Flexible region arrangement of the UC-3 Primer

Flexible region Sequence 5' to 3'	Applicable Strain
TTAAAACAGCCTGTGGGTT	Coxsackievirus: A2, A3, A4, A5, A6, A7, A8, A9, A10, A12, A14, A16, A17, A20, A21 B1, B2, B3, B4, B5, B6<br>Enterovirus : A71, A76, A89, A90, A91, A92, A120, B69, B73, B74, B75, B77, B78, B82, B84, B85, B86, B87, B88, B93, B97, B98, B101, B106, C99, C105, C116,<br>Echovirus : E2, E3, E4, E5, E6, E7, E9, E11, E12, E13, E14, E15, E16, E17, E18, E19, E20, E21, E24, E25, E26, E27, E29, E30, E31, E32, E33,<br>HumanEnterovirus : 79, 107
TTAAAACAGCTCTGGGGTT	Coxsackievirus : A1, A11, A13, A15, A18, A19, A22, A24, C96<br>Enterovirus : B80, B81, B83, B100, D94, D111<br>Human poliovirus: 1, 2, 3<br>Human enterovirus : 70
TTAAAACAGCCTGGGGGTT	Enterovirus : C109
TTAAAACAGCTTGGGGGTT	Human enterovirus : C104
TTAAAACAGCCTTGGGGTT	Enterovirus : D68

Supplementary Table 2. List of Enterovirus genome references used in this study

Number	Strain Name	Virus Type	GenBank Accession	Sequence Length	Pango Genome Lineage	Collection Date	Serotype
1	CV-A10/P1005/2013/China	Coxsackievirus A10	KP289394	7412	2013	Human	A type
2	CV-A10/P118/2013/China	Coxsackievirus A10	KP289395	7411	2013	Human	A type
3	CV-A10/P448/2013/China	Coxsackievirus A10	KP289396	7412	2013	Human	A type
4	CV-A10/P460/2013/China	Coxsackievirus A10	KP289397	7411	2013	Human	A type
5	CV-A10/P630/2013/China	Coxsackievirus A10	KP289398	7412	2013	Human	A type
6	CV-A10/P638/2013/China	Coxsackievirus A10	KP289399	7412	2013	Human	A type
7	CV-A10/P639/2013/China	Coxsackievirus A10	KP289400	7412	2013	Human	A type
8	CV-A10/P670/2013/China	Coxsackievirus A10	KP289401	7412	2013	Human	A type
9	CV-A10/P73/2013/China	Coxsackievirus A10	KP289402	7412	2013	Human	A type
10	CV-A10/P737/2013/China	Coxsackievirus A10	KP289403	7411	2013	Human	A type

11	CV-A10/P864/2013/China	Coxsackievirus A10	KP289404	7412	2013	Human	A type
12	CV-A10/P866/2013/China	Coxsackievirus A10	KP289405	7412	2013	Human	A type
13	CV-A10/P91/2013/China	Coxsackievirus A10	KP289406	7412	2013	Human	A type
14	CV-A10/P911/2013/China	Coxsackievirus A10	KP289407	7412	2013	Human	A type
15	CV-A10/P935/2013/China	Coxsackievirus A10	KP289408	7412	2013	Human	A type
16	CV-A10/P962/2013/China	Coxsackievirus A10	KP289409	7412	2013	Human	A type
17	CV-A10/P978/2013/China	Coxsackievirus A10	KP289410	7412	2013	Human	A type
18	CVA10/JB141310010	Coxsackievirus A10	KJ641623	7412	06/28/2013	Human	A type
19	CVA10/SD/CHN/09	Coxsackievirus A10	HQ728262	7412	07/2009	Human	A type
20	FY01/AH/CHN/2013	Coxsackievirus A10	KP009574	7351	12/20/2013	Human	A type
21	FY02/AH/CHN/2013	Coxsackievirus A10	KP009575	7351	12/20/2013	Human	A type
22	FY03/AH/CHN/2013	Coxsackievirus A10	KP009576	7351	12/20/2013	Human	A type
23	FY04/AH/CHN/2013	Coxsackievirus A10	KP009577	7351	12/20/2013	Human	A type

24	FY05/AH/CHN/2013	Coxsackievirus A10	KP00957 8	7351	12/20/2 013	Human	A type
25	FY06/AH/CHN/2013	Coxsackievirus A10	KP00957 9	7351	12/20/2 013	Human	A type
26	FY07/AH/CHN/2013	Coxsackievirus A10	KP00958 0	7351	12/20/2 013	Human	A type
27	FY08/AH/CHN/2013	Coxsackievirus A10	KP00958 1	7351	12/20/2 013	Human	A type
28	QD-HDH507/SD/CHN/2011	Coxsackievirus A12	KF422143	7396	05/2011	Human	A type
29	QD-LXH535/SD/CHN/2009	Coxsackievirus A12	KF422142	7396	04/2009	Human	A type
30	Texas-12	Coxsackievirus A12	AY421768	7404	-N/A-	Human	A type
31	G-14	Coxsackievirus A14	AY421769	7415	-N/A-	Human	A type
32	PZ05Y/JS/2012	Coxsackievirus A14	KP03648 2	7404	04/15/2 012	Human	A type
33	PZ15G/JS/2012	Coxsackievirus A14	KP03648 3	7400	-N/A-	Human	A type
34	SEN-14-254	Coxsackievirus A14	MG67248 2	7353	-N/A-	Human	A type
35	CV-A16/P10/2013/China	Coxsackievirus A16	KP289411	7409	2013	Human	A type
36	CV-A16/P1014/2013/China	Coxsackievirus A16	KP28941 2	7410	2013	Human	A type
37	CV-A16/P187/2013/China	Coxsackievirus A16	KP28941 3	7408	2013	Human	A type
38	CV-A16/P255/2013/China	Coxsackievirus A16	KP28941 4	7409	2013	Human	A type

39	CV-A16/P301/2013/China	Coxsackievirus A16	KP289415	7410	2013	Human	A type
40	CV-A16/P83/2013/China	Coxsackievirus A16	KP289416	7409	2013	Human	A type
41	CVA16-WIBP-P4-731	Coxsackievirus A16	KF924762	7410	05/27/2010	Human	A type
42	CVA16/SZ29/CHN/2014	Coxsackievirus A16	KM215267	7410	05/2014	Human	A type
43	DL16	Coxsackievirus A16	KF991007	7377	05/18/2012	Human	A type
44	FJ09-01	Coxsackievirus A16	KF193628	7400	2009	Human	A type
45	FJ09-02	Coxsackievirus A16	KF193627	7400	2009	Human	A type
46	FJ10-03	Coxsackievirus A16	KF193626	7400	2010	Human	A type
47	FY18	Coxsackievirus A16	EU812514	7414	05/2008	Human	A type
48	G-10	Coxsackievirus A16	U05876	7413	-N/A-	Human	A type
49	G08	Coxsackievirus A16	KC342228	7410	-N/A-	Human	A type
50	G20	Coxsackievirus A16	JN590244	7410	05/24/2010	Human	A type
51	GX10-01	Coxsackievirus A16	KF193625	7400	2010	Human	A type
52	GZ08	Coxsackievirus A16	FJ198212	7410	06/2008	Human	A type
53	HN09-02	Coxsackievirus A16	KF193624	7400	2009	Human	A type
54	HN11-03	Coxsackievirus A16	KF193623	7420	2011	Human	A type

55	HN1662/HN/CHN/2010	Coxsackievirus A16	JN674176	7411	2010	Human	A type
56	KMM/08	Coxsackievirus A16	HQ42314<br>1	7409	05/2008	Human	A type
57	Kor08-CVA16	Coxsackievirus A16	JX839965	7411	06/2008	Human	A type
58	MAV	Coxsackievirus A16	KC69583<br>0	7417	-N/A-	Human	A type
59	Ningbo.CHN/028-2/2009	Coxsackievirus A16	JQ354992	7409	04/02/2<br>009	Human	A type
60	NJ10-31	Coxsackievirus A16	KC75523<br>2	7454	2010	Human	A type
61	NJ10-75	Coxsackievirus A16	KC75523<br>3	7457	2010	Human	A type
62	PM-00033-07	Coxsackievirus A16	JQ746660	7403	2007	Human	A type
63	PM-12284-99	Coxsackievirus A16	JQ746661	7391	1999	Human	A type
64	PM-12727-99	Coxsackievirus A16	JQ746662	7395	1999	Human	A type
65	PM-13884-97	Coxsackievirus A16	JQ746663	7400	1997	Human	A type
66	PM-13998-00	Coxsackievirus A16	JQ746664	7388	2000	Human	A type
67	PM-14660-97	Coxsackievirus A16	JQ746665	7400	1997	Human	A type
68	PM-15765-00	Coxsackievirus A16	JQ746666	7396	2000	Human	A type
69	PM-15922-00	Coxsackievirus A16	JQ746667	7393	2000	Human	A type
70	PM-1651402-06	Coxsackievirus A16	JQ746668	7355	2006	Human	A type
71	PM-16809-98	Coxsackievirus A16	JQ746669	7386	1998	Human	A type
72	PM-16985-98	Coxsackievirus A16	JQ746670	7395	1998	Human	A type

73	PM-1791021-07	Coxsackievirus A16	JQ746671	7401	2007	Human	A type
74	PM-1795457-07	Coxsackievirus A16	JQ746672	7387	2007	Human	A type
75	PM-22159-02	Coxsackievirus A16	JQ746673	7400	2002	Human	A type
76	shzh05-1	Coxsackievirus A16	EU26265 8	7410	-N/A-	Human	A type
77	SZ/HK08-3	Coxsackievirus A16	GQ27936 8	7409	05/2008	Human	A type
78	SZ/HK08-7	Coxsackievirus A16	GQ27937 1	7409	05/2008	Human	A type
79	THA-CA16-069	Coxsackievirus A16	JF738004	7304	07/2010	Human	A type
80	THA-CA16-090	Coxsackievirus A16	JF738003	7278	02/2010	Human	A type
81	TS10/07	Coxsackievirus A16	JX068827	7411	07/09/2 010	Human	A type
82	TS10/08	Coxsackievirus A16	JX068829	7411	08/2010	Human	A type
83	Wh16	Coxsackievirus A16	KM51610 2	7406	06/2010	Human	A type
84	Wuhan0109/HuB/CHN/2011	Coxsackievirus A16	JX986740	7411	2011	Human	A type
85	Wuhan0127/HuB/CHN/2011	Coxsackievirus A16	JX986742	7411	2011	Human	A type
86	Wuhan0157/HuB/CHN/2011	Coxsackievirus A16	JX986741	7411	2011	Human	A type
87	CV-A2/P14/2013/China	Coxsackievirus A2	KP28935 7	7398	2013	Human	A type
88	CV-A2/P153/2013/China	Coxsackievirus A2	KP28935 8	7398	2013	Human	A type
89	CV-A2/P373/2013/China	Coxsackievirus A2	KP28935	7398	2013	Human	A type

			9				
90	CV-A2/P478/2013/China	Coxsackievirus A2	KP28936 0	7398	2013	Human	A type
91	CV-A2/P489/2013/China	Coxsackievirus A2	KP28936 1	7398	2013	Human	A type
92	CVA2/SD/CHN/09	Coxsackievirus A2	HQ72825 9	7383	03/2009	Human	A type
93	Fleetwood	Coxsackievirus A2	AY421760	7398	-N/A-	Human	A type
94	Olson	Coxsackievirus A3	AY421761	7395	-N/A-	Human	A type
95	1047/SH/CHN/2010	Coxsackievirus A4	KJ541164	7411	2010	Human	A type
96	701/SH/CHN/2010	Coxsackievirus A4	KJ541163	7411	2010	Human	A type
97	CV-A4/P1033/2013/China	Coxsackievirus A4	KP28944 2	7449	2013	Human	A type
98	CVA4/FT/CHN/05	Coxsackievirus A4	KP67698 4	7465	05/2011	Human	A type
99	CVA4/FT/CHN/07	Coxsackievirus A4	KP67698 5	7468	05/2011	Human	A type
100	CVA4/FT/CHN/27	Coxsackievirus A4	KP67698 6	7461	05/2011	Human	A type
101	CVA4/SZ/CHN/09	Coxsackievirus A4	HQ72826 0	7434	05/2009	Human	A type
102	High Point	Coxsackievirus A4	AY421762	7434	-N/A-	Human	A type
103	CV-A5/P74/2013/China	Coxsackievirus A5	KP28936 2	7406	2013	Human	A type
104	CV-A5/P836/2013/China	Coxsackievirus A5	KP28936	7406	2013	Human	A type

			3				
105	CV-A5/P99/2013/China	Coxsackievirus A5	KP28936 4	7406	2013	Human	A type
106	CVA5/SD/CHN/09	Coxsackievirus A5	HQ72826 1	7406	07/2009	Human	A type
107	Swartz	Coxsackievirus A5	AY421763	7400	-N/A-	Human	A type
108	1232/SH/CHN/2010	Coxsackievirus A6	KJ541167	7371	2010	Human	A type
109	12743/GZ/CHN/2013	Coxsackievirus A6	KR81599 2	7621	01/06/2 013	Human	A type
110	1827/SH/CHN/2011	Coxsackievirus A6	KJ541166	7370	2011	Human	A type
111	3913/SH/CHN/2011	Coxsackievirus A6	KJ541168	7369	2011	Human	A type
112	4368/SH/CHN/2012	Coxsackievirus A6	KJ541169	7370	2012	Human	A type
113	4592/SH/CHN/2012	Coxsackievirus A6	KJ541159	7412	2012	Human	A type
114	4645/SH/CHN/2012	Coxsackievirus A6	KJ541157	7412	2012	Human	A type
115	5039/SH/CHN/2013	Coxsackievirus A6	KJ541160	7411	2013	Human	A type
116	5047/SH/CHN/2013	Coxsackievirus A6	KJ541156	7412	2013	Human	A type
117	5056/SH/CHN/2013	Coxsackievirus A6	KJ541161	7411	2013	Human	A type
118	5069/SH/CHN/2013	Coxsackievirus A6	KJ541158	7412	2013	Human	A type
119	5084/SH/CHN/2013	Coxsackievirus A6	KJ541154	7412	2013	Human	A type
120	CA6/CC13/57	Coxsackievirus A6	KM27937 9	7434	2013	Human	A type
121	CV-A6/P115/2013/China	Coxsackievirus A6	KP28936 7	7435	2013	Human	A type

122	CV-A6/P143/2013/China	Coxsackievirus A6	KP289368	7433	2013	Human	A type
123	CV-A6/P169/2013/China	Coxsackievirus A6	KP289369	7435	2013	Human	A type
124	CV-A6/P2/2013/China	Coxsackievirus A6	KP289370	7434	2013	Human	A type
125	CV-A6/P223/2013/China	Coxsackievirus A6	KP289371	7434	2013	Human	A type
126	CV-A6/P225/2013/China	Coxsackievirus A6	KP289372	7434	2013	Human	A type
127	CV-A6/P246/2013/China	Coxsackievirus A6	KP289373	7434	2013	Human	A type
128	CV-A6/P278/2013/China	Coxsackievirus A6	KP289374	7434	2013	Human	A type
129	CV-A6/P289/2013/China	Coxsackievirus A6	KP289365	7476	2013	Human	A type
130	CV-A6/P309/2013/China	Coxsackievirus A6	KP289375	7433	2013	Human	A type
131	CV-A6/P345/2013/China	Coxsackievirus A6	KP289376	7434	2013	Human	A type
132	CV-A6/P358/2013/China	Coxsackievirus A6	KP289377	7433	2013	Human	A type
133	CV-A6/P360/2013/China	Coxsackievirus A6	KP289378	7433	2013	Human	A type
134	CV-A6/P361/2013/China	Coxsackievirus A6	KP289379	7434	2013	Human	A type

135	CV-A6/P362/2013/China	Coxsackievirus A6	KP289380	7434	2013	Human	A type
136	CV-A6/P366/2013/China	Coxsackievirus A6	KP289381	7435	2013	Human	A type
137	CV-A6/P406/2013/China	Coxsackievirus A6	KP289382	7433	2013	Human	A type
138	CV-A6/P423/2013/China	Coxsackievirus A6	KP289366	7473	2013	Human	A type
139	CV-A6/P426/2013/China	Coxsackievirus A6	KP289383	7433	2013	Human	A type
140	CV-A6/P6/2013/China	Coxsackievirus A6	KP289384	7434	2013	Human	A type
141	CV-A6/P66/2013/China	Coxsackievirus A6	KP289385	7434	2013	Human	A type
142	CV-A6/P674/2013/China	Coxsackievirus A6	KP289386	7433	2013	Human	A type
143	CV-A6/P695/2013/China	Coxsackievirus A6	KP289387	7433	2013	Human	A type
144	CV-A6/P702/2013/China	Coxsackievirus A6	KP289388	7434	2013	Human	A type
145	CV-A6/P728/2013/China	Coxsackievirus A6	KP289389	7433	2013	Human	A type
146	CV-A6/P731/2013/China	Coxsackievirus A6	KP289390	7434	2013	Human	A type
147	CV-A6/P786/2013/China	Coxsackievirus A6	KP289391	7433	2013	Human	A type

148	CV-A6/P794/2013/China	Coxsackievirus A6	KP289392	7433	2013	Human	A type
149	CV-A6/P874/2013/China	Coxsackievirus A6	KP289393	7433	2013	Human	A type
150	Finland/2008	Coxsackievirus A6	KM114057	7423	2008	Human	A type
151	Gdula	Coxsackievirus A6	AY421764	7434	-N/A-	Human	A type
152	HN421	Coxsackievirus A6	JQ964234	7434	2011	Human	A type
153	Kyoto1	Coxsackievirus A6	AB779614	7434	1999	Human	A type
154	Kyoto2	Coxsackievirus A6	AB779616	7434	2003	Human	A type
155	Kyoto3	Coxsackievirus A6	AB779615	7434	2009	Human	A type
156	Kyoto4	Coxsackievirus A6	AB779617	7434	2009	Human	A type
157	Kyoto5	Coxsackievirus A6	AB779618	7434	2009	Human	A type
158	PF001/SH/CHN/2013	Coxsackievirus A6	KJ541165	7412	2013	Human	A type
159	PF1/SH/CHN/2013	Coxsackievirus A6	KJ612513	7433	2013	Human	A type
160	PF19/SH/CHN/2013	Coxsackievirus A6	KJ541155	7412	2013	Human	A type
161	PF3/SH/CHN/2013	Coxsackievirus A6	KJ541162	7411	2013	Human	A type
162	Shizuoka 18	Coxsackievirus A6	AB678778	7434	2011	Human	A type
163	SZc173/13	Coxsackievirus A6	KF682362	7434	2013	Human	A type

164	SZc294/13	Coxsackievirus A6	KF682363	7434	2013	Human	A type
165	TW-2007-00141	Coxsackievirus A6	KR70630 9	7460	2007	Human	A type
166	TW/1537/2011	Coxsackievirus A6	JN582001	7434	2011	Human	A type
167	TW/20/09	Coxsackievirus A6	JQ946050	7390	01/06/2 009	Human	A type
168	TW/273/09	Coxsackievirus A6	JQ946051	7392	03/03/2 009	Human	A type
169	TW/295/09	Coxsackievirus A6	JQ946052	7392	03/04/2 009	Human	A type
170	TW/391/10	Coxsackievirus A6	JQ946053	7390	06/07/2 010	Human	A type
171	TW/399/10	Coxsackievirus A6	JQ946054	7390	06/08/2 010	Human	A type
172	TW/409/10	Coxsackievirus A6	JQ946055	7391	06/08/2 010	Human	A type
173	1162/GZ/CHN/2007	Coxsackievirus A7	MT17978 6	7407	2007	Human	A type
174	CV-A7|37183|TKM|2010	Coxsackievirus A7	LR027540	7398	-N/A-	Human	A type
175	ET1080	Coxsackievirus A7	GU94282 1	7404	-N/A-	Human	A type
176	LEV8	Coxsackievirus A7	JQ041367	7404	2011	Human	A type
177	Parker	Coxsackievirus A7	AY421765	7404	-N/A-	Human	A type
178	CV-A8/P82/2013/China	Coxsackievirus A8	KP28943 5	7399	2013	Human	A type

179	CVA8/SZ124/CHN/2012	Coxsackievirus A8	KM609476	7396	05/2012	Human	A type
180	CVA8/SZ127/CHN/2012	Coxsackievirus A8	KM609477	7396	05/2012	Human	A type
181	CVA8/SZ141/CHN/2012	Coxsackievirus A8	KM609478	7396	06/2012	Human	A type
182	CVA8/SZ157/CHN/2012	Coxsackievirus A8	KM609479	7396	06/2012	Human	A type
183	CVA8/SZ266/CHN/2014	Coxsackievirus A8	KP765687	7396	06/2014	Human	A type
184	CVA8/SZ39/CHN/2013	Coxsackievirus A8	KM609480	7396	05/2013	Human	A type
185	CVA8/SZ40/CHN/2013	Coxsackievirus A8	KM609481	7396	05/2013	Human	A type
186	CVA8/SZ93/CHN/2012	Coxsackievirus A8	KM609475	7396	05/2012	Human	A type
187	MAD-2741-11	Enterovirus A120	LK021688	7407	2011	Human	A type
188	Q0082/XZ/CHN/2000	Enterovirus A120	MT123346	7406	2000	Human	A type
189	TRT/2013-23196	Enterovirus A120	MT081367	7408	2013	Human	A type
190	OM112t (P12)	enterovirus A123	AF326761	7348	-N/A-	Human	A type
191	OM22 (P15)	enterovirus A124	AF326764	7393	-N/A-	Human	A type
192	A13	enterovirus A125	AF326750	7389	-N/A-	Human	A type
193	03-0-002	Enterovirus A71	KP691644	7379	11/06/2013	Human	A type

194	03-0-003	Enterovirus A71	KP691649	7376	11/06/2013	Human	A type
195	03-KOR-00	Enterovirus A71	DQ341356	7409	-N/A-	Human	A type
196	06-KOR-00	Enterovirus A71	DQ341355	7409	-N/A-	Human	A type
197	0964/SYD/98	Enterovirus A71	JN992283	7409	-N/A-	Human	A type
198	0964/SYD/98	Enterovirus A71	JN992285	7409	-N/A-	Human	A type
199	1/SHENZHEN/08/China/HFMD/2008	Enterovirus A71	FJ607334	7404	05/2008	Human	A type
200	1095-LPS1	Enterovirus A71	AB550333	7409	-N/A-	Human	A type
201	1095-org	Enterovirus A71	AB550332	7409	-N/A-	Human	A type
202	121/SHENZHEN/08/China/HFMD Fatal/2008	Enterovirus A71	FJ607337	7404	05/2008	Human	A type
203	1245a/98/tw	Enterovirus A71	AF176044	7433	-N/A-	Human	A type
204	1M-AUS-12-00	Enterovirus A71	DQ341361	7409	-N/A-	Human	A type
205	2006-52-9	Enterovirus A71	KP266579	7406	11/14/2006	Human	A type
206	2008-43-16	Enterovirus A71	KP266572	7407	12/02/2008	Human	A type
207	2010FJLY008	Enterovirus A71	HQ426649	7403	06/2010	Human	A type
208	202/Jingdezhen/China/HFMD Severe/2011	Enterovirus A71	KC109780	7405	10/2011	Human	A type

209	237-TW86	Enterovirus A71	FJ357380	7414	1986	Human	A type
210	26M/AUS/4/99	Enterovirus A71	EU36484<br>1	7412	-N/A-	Human	A type
211	26M/AUS/4/99	Enterovirus A71	EU37600<br>4	7412	-N/A-	Human	A type
212	26M/AUS/4/99	Enterovirus A71	EU37600<br>5	7412	-N/A-	Human	A type
213	26M/AUS/4/99/GuaR1	Enterovirus A71	JQ950555	6582	09/13/2<br>010	Human	A type
214	28/SHENZHEN/08/China/HFMD/2008	Enterovirus A71	FJ607336	7403	05/2008	Human	A type
215	306A	Enterovirus A71	KJ746493	7424	2010	Human	A type
216	306B	Enterovirus A71	KJ746494	7424	2010	Human	A type
217	7F-AUS-6-99	Enterovirus A71	DQ34135<br>7	7409	-N/A-	Human	A type
218	804/NO/03	Enterovirus A71	DQ45207<br>4	7410	2003	Human	A type
219	87-2008 Xi'an Shaanxi	Enterovirus A71	HM00320<br>7	7414	2008	Human	A type
220	A2011125	Enterovirus A71	KF134486	7338	2011	Human	A type
221	AFP2001064/EV71/GX/CHN/2001	Enterovirus A71	JQ742001	7406	04/2001	Human	A type
222	AFP2001071/EV71/GX/CHN/2001	Enterovirus A71	JQ742002	7406	04/2001	Human	A type
223	AH08/06	Enterovirus A71	HQ61114<br>8	7405	2008	Human	A type
224	Anhui1-09-China	Enterovirus A71	GQ99498<br>8	7395	04/2009	Human	A type

225	Anhui2007	Enterovirus A71	KC954662	7405	2007	Human	A type
226	B5-03172-TW-2011	Enterovirus A71	KF154354	7414	11/05/2011	Human	A type
227	B5-96015-TW-2012	Enterovirus A71	KF154355	7411	04/18/2012	Human	A type
228	BC08	Enterovirus A71	JQ514785	7406	08/2008	Human	A type
229	BJ08	Enterovirus A71	FJ828519	7405	2008	Human	A type
230	BJ08-Z004-3	Enterovirus A71	FJ606447	7405	07/2008	Human	A type
231	BJ08-Z011-4	Enterovirus A71	FJ606448	7405	07/2008	Human	A type
232	BJ08-Z020-1	Enterovirus A71	FJ606449	7405	07/2008	Human	A type
233	BJ08-Z025-5	Enterovirus A71	FJ606450	7405	07/2008	Human	A type
234	BJ09/07	Enterovirus A71	JQ319054	7406	2009	Human	A type
235	BJ110	Enterovirus A71	HM002486	7406	05/30/2008	Human	A type
236	BJ293	Enterovirus A71	HM053669	7405	07/28/2008	Human	A type
237	BJ303	Enterovirus A71	HM002487	7406	07/31/2008	Human	A type
238	BJ366	Enterovirus A71	HM002488	7405	04/10/2009	Human	A type
239	BJ393	Enterovirus A71	HM053670	7405	04/30/2009	Human	A type
240	EV71/Homo sapiens/KHM/271/2012	Enterovirus A71	KP308433	7360	07/29/2012	Human	A type

241	EV71/Homo sapiens/KHM/272/2012	Enterovirus A71	KP308432	7360	07/29/2012	Human	A type
242	EV71/Homo sapiens/KHM/275/2012	Enterovirus A71	KP308441	7360	07/31/2012	Human	A type
243	EV71/Homo sapiens/KHM/277/2012	Enterovirus A71	KP308414	7345	07/30/2012	Human	A type
244	EV71/Homo sapiens/KHM/280/2012	Enterovirus A71	KP308430	7361	08/07/2012	Human	A type
245	EV71/Homo sapiens/KHM/287/2012	Enterovirus A71	KP308428	7330	08/12/2012	Human	A type
246	EV71/Homo sapiens/KHM/293/2012	Enterovirus A71	KP308437	7360	08/20/2012	Human	A type
247	EV71/Homo sapiens/KHM/295/2012	Enterovirus A71	KP308436	7360	08/21/2012	Human	A type
248	EV71/Homo sapiens/KHM/297/2012	Enterovirus A71	KP308403	7358	08/26/2012	Human	A type
249	EV71/Homo sapiens/KHM/298/2012	Enterovirus A71	KP308434	7360	08/25/2012	Human	A type
250	EV71/Homo sapiens/KHM/299/2012	Enterovirus A71	KP308421	7255	08/25/2012	Human	A type
251	EV71/Homo sapiens/KHM/300/2012	Enterovirus A71	KP308451	7360	09/06/2012	Human	A type
252	EV71/Homo sapiens/KHM/302/2012	Enterovirus A71	KP308438	7359	09/09/2012	Human	A type
253	EV71/Homo sapiens/KHM/304/2012	Enterovirus A71	KP308408	7361	09/16/2012	Human	A type

254	EV71/Homo sapiens/KHM/310/2012	Enterovirus A71	KP308402	7360	11/13/2012	Human	A type
255	EV71/Homo sapiens/KHM/311/2012	Enterovirus A71	KP308439	7360	11/23/2012	Human	A type
256	EV71/Homo sapiens/VNM/108/2012	Enterovirus A71	KJ686214	7360	03/29/2012	Human	A type
257	EV71/Homo sapiens/VNM/109/2012	Enterovirus A71	KJ686150	7360	03/30/2012	Human	A type
258	EV71/Homo sapiens/VNM/11/2011	Enterovirus A71	KJ686242	7360	10/11/2011	Human	A type
259	EV71/Homo sapiens/VNM/110/2012	Enterovirus A71	KJ686174	7360	03/31/2012	Human	A type
260	EV71/Homo sapiens/VNM/111/2012	Enterovirus A71	KJ686156	7360	03/31/2012	Human	A type
261	EV71/Homo sapiens/VNM/113/2012	Enterovirus A71	KJ686225	7366	04/03/2012	Human	A type
262	EV71/Homo sapiens/VNM/114/2012	Enterovirus A71	KJ686205	7360	04/07/2012	Human	A type
263	EV71/Homo sapiens/VNM/116/2012	Enterovirus A71	KJ686168	7360	04/11/2012	Human	A type
264	EV71/Homo sapiens/VNM/117/2012	Enterovirus A71	KJ686138	7360	04/12/2012	Human	A type
265	EV71/Homo sapiens/VNM/119/2012	Enterovirus A71	KJ686267	7360	04/12/2012	Human	A type
266	EV71/Homo sapiens/VNM/12/2011	Enterovirus A71	KJ686301	7360	10/11/2011	Human	A type

267	EV71/Homo sapiens/VNM/121/2012	Enterovirus A71	KJ686277	7366	04/16/2012	Human	A type
268	EV71/Homo sapiens/VNM/122/2012	Enterovirus A71	KJ686132	7360	04/17/2012	Human	A type
269	EV71/Homo sapiens/VNM/123/2012	Enterovirus A71	KJ686131	7366	04/18/2012	Human	A type
270	EV71/Homo sapiens/VNM/127/2012	Enterovirus A71	KJ686248	7359	04/21/2012	Human	A type
271	EV71/Homo sapiens/VNM/128/2012	Enterovirus A71	KJ686220	7360	04/22/2012	Human	A type
272	EV71/Homo sapiens/VNM/129/2012	Enterovirus A71	KJ686296	7366	04/22/2012	Human	A type
273	EV71/Homo sapiens/VNM/13/2011	Enterovirus A71	KJ686158	7360	10/11/2011	Human	A type
274	EV71/Homo sapiens/VNM/159/2012	Enterovirus A71	KJ686253	7286	06/29/2012	Human	A type
275	EV71/Homo sapiens/VNM/16/2011	Enterovirus A71	KJ686275	7359	10/16/2011	Human	A type
276	EV71/Homo sapiens/VNM/160/2012	Enterovirus A71	KJ686199	7358	07/04/2012	Human	A type
277	EV71/Homo sapiens/VNM/161/2012	Enterovirus A71	KJ686157	7359	07/20/2012	Human	A type
278	EV71/Homo sapiens/VNM/162/2012	Enterovirus A71	KJ686201	7359	07/21/2012	Human	A type
279	EV71/Homo sapiens/VNM/163/2012	Enterovirus A71	KJ686266	7359	08/20/2012	Human	A type

280	EV71/Homo sapiens/VNM/164/2012	Enterovirus A71	KJ686219	7360	08/23/2012	Human	A type
281	EV71/Homo sapiens/VNM/167/2012	Enterovirus A71	KJ686295	7353	09/08/2012	Human	A type
282	EV71/Homo sapiens/VNM/168/2012	Enterovirus A71	KJ686176	7366	09/20/2012	Human	A type
283	EV71/Homo sapiens/VNM/170/2012	Enterovirus A71	KJ686252	7360	10/06/2012	Human	A type
284	EV71/Homo sapiens/VNM/171/2012	Enterovirus A71	KJ686270	7366	10/23/2012	Human	A type
285	EV71/Homo sapiens/VNM/173/2012	Enterovirus A71	KJ686250	7359	10/30/2012	Human	A type
286	EV71/Homo sapiens/VNM/174/2012	Enterovirus A71	KJ686211	7366	11/02/2012	Human	A type
287	EV71/Homo sapiens/VNM/176/2012	Enterovirus A71	KJ686234	7366	11/03/2012	Human	A type
288	EV71/Homo sapiens/VNM/177/2012	Enterovirus A71	KJ686264	7366	11/07/2012	Human	A type
289	EV71/P1027/2013/China	Enterovirus A71	KP289417	7412	2013	Human	A type
290	EV71/P1031/2013/China	Enterovirus A71	KP289418	7405	2013	Human	A type
291	EV71/P1034/2013/China	Enterovirus A71	KP289419	7405	2013	Human	A type
292	EV71/P123/2013/China	Enterovirus A71	KP289420	7405	2013	Human	A type

293	EV71/P156/2013/China	Enterovirus A71	KP289421	7405	2013	Human	A type
294	EV71/P16/2013/China	Enterovirus A71	KP289422	7405	2013	Human	A type
295	EV71/P222/2013/China	Enterovirus A71	KP289423	7405	2013	Human	A type
296	EV71/P267/2013/China	Enterovirus A71	KP289424	7405	2013	Human	A type
297	EV71/P352/2013/China	Enterovirus A71	KP289425	7405	2013	Human	A type
298	EV71/P40/2013/China	Enterovirus A71	KP289426	7405	2013	Human	A type
299	EV71/P454/2013/China	Enterovirus A71	KP289427	7414	2013	Human	A type
300	EV71/P63/2013/China	Enterovirus A71	KP289428	7412	2013	Human	A type
301	EV71/P654/2013/China	Enterovirus A71	KP289429	7405	2013	Human	A type
302	EV71/P868/2013/China	Enterovirus A71	KP289430	7405	2013	Human	A type
303	EV71/P977/2013/China	Enterovirus A71	KP289431	7412	2013	Human	A type
304	EV71/P990/2013/China	Enterovirus A71	KP289432	7414	2013	Human	A type
305	EV71/Queenmary/HongKong/2012	Enterovirus A71	KF444809	7405	2012	Human	A type
306	EV71/wuhan/3018/2010	Enterovirus A71	KF501389	7408	2010	Human	A type

307	EV71/Xiamen/2009	Enterovirus A71	JN964686	7430	2009	Human	A type
308	EV71/Zhejiang08	Enterovirus A71	EU86450 7	7406	2008	Human	A type
309	EV92_07	Enterovirus A71	HQ64716 8	7419	08/2007	Human	A type
310	Fuyang-0805	Enterovirus A71	FJ439769	7405	05/2008	Human	A type
311	04360/SD/CHN/2004/EV76	Enterovirus A76	JF905564	7441	08/15/2 004	Human	A type
312	Human-AY697458	Enterovirus A76	AY697458	7438	-N/A-	Human	A type
313	BAN00-10359	Enterovirus A89	AY697459	7429	-N/A-	Human	A type
314	KSYPH-TRMH22F/XJ/CHN/2011	Enterovirus A89	KT277550	7429	09/16/2 011	Human	A type
315	01336/SD/CHN/EV90	Enterovirus A90	JX390654	7425	2001	Human	A type
316	01421/SD/CHN/EV90	Enterovirus A90	JX390655	7424	2001	Human	A type
317	03446/SD/CHN/EV90	Enterovirus A90	JX390656	7423	2003	Human	A type
318	10-2879-1	Enterovirus A90	MH11803 1	7434	2010	Human	A type
319	BAN99-10399	Enterovirus A90	AY697460	7425	-N/A-	Human	A type
320	CAM1956	Enterovirus A90	AB19287 7	7425	-N/A-	Human	A type
321	F950027	Enterovirus A90	AY773285	7438	-N/A-	Human	A type
322	HTH08F/XJ/CHN/2011	Enterovirus A90	MG25303 3	7423	2011	Human	A type
323	KSH13F/XJ/CHN/2011	Enterovirus A90	MG25303	7423	2011	Human	A type

			5				
324	MYH12F/XJ/CHN/2011	Enterovirus A90	MG253032	7423	2011	Human	A type
325	PSH40F/XJ/CHN/2011	Enterovirus A90	MG253034	7425	2011	Human	A type
326	SCH05F/XJ/CHN/2011	Enterovirus A90	MG253036	7424	2011	Human	A type
327	BAN00-10406	Enterovirus A91	AY697461	7427	-N/A-	Human	A type
328	RJG7	Enterovirus A92	EF667344	7379	-N/A-	Human	A type
329	09-1701-1	Human coxsackievirus A10	MH118089	7495	2009	Human	A type
330	09-1895-1	Human coxsackievirus A10	MH118088	7414	2009	Human	A type
331	09-1996-1	Human coxsackievirus A10	MH118087	7550	2009	Human	A type
332	09-207-1	Human coxsackievirus A10	MH118086	7412	2009	Human	A type
333	09-2093-1	Human coxsackievirus A10	MH118085	7537	2009	Human	A type
334	09-3318-1	Human coxsackievirus A10	MH118084	7534	2009	Human	A type
335	10-1183-1	Human coxsackievirus A10	MH118083	7410	2010	Human	A type
336	10-1989-1	Human coxsackievirus A10	MH118082	7487	2010	Human	A type
337	10-2064-1	Human coxsackievirus A10	MH11808	7418	2010	Human	A type

			1				
338	10-2565-1	Human coxsackievirus A10	MH11808<br>0	7383	2010	Human	A type
339	10-2792-1	Human coxsackievirus A10	MH11807<br>9	7447	2010	Human	A type
340	10-3408-2	Human coxsackievirus A10	MH11807<br>8	7414	2010	Human	A type
341	10-3432-2	Human coxsackievirus A10	MH11807<br>7	7546	2010	Human	A type
342	10-3445-2	Human coxsackievirus A10	MH11807<br>6	7467	2010	Human	A type
343	10-3749-1	Human coxsackievirus A10	MH11807<br>5	7378	2010	Human	A type
344	10-381-2	Human coxsackievirus A10	MH11807<br>4	7488	2010	Human	A type
345	10-4214-1	Human coxsackievirus A10	MH11807<br>3	7415	2010	Human	A type
346	10-923-1	Human coxsackievirus A10	MH11807<br>2	7427	2010	Human	A type
347	11-1208-1	Human coxsackievirus A10	MH11807<br>1	7361	2011	Human	A type
348	11-1415-1	Human coxsackievirus A10	MH11807<br>0	7418	2011	Human	A type
349	11-1525-1	Human coxsackievirus A10	MH11806<br>9	7495	2011	Human	A type
350	11-1828-1	Human coxsackievirus A10	MH11806	7433	2011	Human	A type

			8				
351	11-2017-1	Human coxsackievirus A10	MH118067	7406	2011	Human	A type
352	11-3186-1	Human coxsackievirus A10	MH118066	7382	2011	Human	A type
353	11-3293-2	Human coxsackievirus A10	MH118065	7389	2011	Human	A type
354	11-363-2	Human coxsackievirus A10	MH118064	7525	2011	Human	A type
355	11-4056-2	Human coxsackievirus A10	MH118063	7510	2011	Human	A type
356	11-4057-2	Human coxsackievirus A10	MH118062	7416	2011	Human	A type
357	11-4519-1	Human coxsackievirus A10	MH144591	7399	2011	Human	A type
358	11-670-1	Human coxsackievirus A10	MH118061	7455	2011	Human	A type
359	11-691-1	Human coxsackievirus A10	MH118060	7517	2011	Human	A type
360	11-759-2	Human coxsackievirus A10	MH144595	7404	2011	Human	A type
361	11-862-1	Human coxsackievirus A10	MH118059	7395	2011	Human	A type
362	11-863-1	Human coxsackievirus A10	MH118058	7428	2011	Human	A type
363	11-964-1	Human coxsackievirus A10	MH11805	7396	2011	Human	A type

			7				
364	11-972-1	Human coxsackievirus A10	MH11805 6	7395	2011	Human	A type
365	12-145-2	Human coxsackievirus A10	MH11805 5	7470	2012	Human	A type
366	12-2067-2	Human coxsackievirus A10	MH11805 4	7495	2012	Human	A type
367	12-4616-1	Human coxsackievirus A10	MH11805 3	7394	2012	Human	A type
368	13-002-1	Human coxsackievirus A10	MH14459 6	7393	2013	Human	A type
369	13-078-1	Human coxsackievirus A10	MH11805 2	7436	2013	Human	A type
370	13-1281-2	Human coxsackievirus A10	MH11805 1	7427	2013	Human	A type
371	13-1473-2	Human coxsackievirus A10	MH11805 0	7406	2013	Human	A type
372	13-1512-1	Human coxsackievirus A10	MH11804 9	7387	2013	Human	A type
373	13-1754-2	Human coxsackievirus A10	MH11804 8	7442	2013	Human	A type
374	13-1779-1	Human coxsackievirus A10	MH11804 7	7409	2013	Human	A type
375	13-1897-1	Human coxsackievirus A10	MH11804 6	7398	2013	Human	A type
376	13-2053-1_A10	Human coxsackievirus A10	MH14460	7400	2013	Human	A type

			3				
377	13-2185-1	Human coxsackievirus A10	MH11804 5	7463	2013	Human	A type
378	13-2357-1	Human coxsackievirus A10	MH11804 4	7482	2013	Human	A type
379	13-2380-2_A10	Human coxsackievirus A10	MH14459 9	7398	2013	Human	A type
380	13-2591-2	Human coxsackievirus A10	MH14459 2	7387	2013	Human	A type
381	15-2711-1	Human coxsackievirus A10	MH14459 0	7432	2015	Human	A type
382	15-3857-2	Human coxsackievirus A10	MH11804 3	7411	2015	Human	A type
383	15-3903-1	Human coxsackievirus A10	MH11804 2	7390	2015	Human	A type
384	16-2871-1	Human coxsackievirus A10	MH11804 1	7511	2017	Human	A type
385	16-2922-1	Human coxsackievirus A10	MH11804 0	7413	2016	Human	A type
386	17-118-2	Human coxsackievirus A10	MH11803 9	7385	2017	Human	A type
387	17-1537-1	Human coxsackievirus A10	MH11803 8	7528	2017	Human	A type
388	17-1573-2	Human coxsackievirus A10	MH11803 7	7413	2017	Human	A type
389	17-1656-1	Human coxsackievirus A10	MH11803	7482	2017	Human	A type

			6				
390	17-1936-1_A10	Human coxsackievirus A10	MH11802 3	7403	2017	Human	A type
391	17-2233-1	Human coxsackievirus A10	MH14459 4	7390	2017	Human	A type
392	17-2249-2	Human coxsackievirus A10	MH11803 5	7550	2017	Human	A type
393	17-2255-1_A10	Human coxsackievirus A10	MH14459 7	7392	2017	Human	A type
394	17-2548-1	Human coxsackievirus A10	MH11803 4	7499	2017	Human	A type
395	17-2579-1	Human coxsackievirus A10	MH11803 3	7482	2017	Human	A type
396	2014-XMCDC-361-CA10	Human coxsackievirus A10	KX76815 6	7395	2014	Human	A type
397	2015-XMCDC-162-CA10	Human coxsackievirus A10	KX76816 3	7395	2015	Human	A type
398	2015-XMCDC-220-CA10	Human coxsackievirus A10	KX76816 6	7442	2015	Human	A type
399	2015-XMCDC-85-CA10	Human coxsackievirus A10	KX76815 9	7366	2015	Human	A type
400	2015-XMCDC-85-CA10	Human coxsackievirus A10	KX76816 0	7366	2015	Human	A type
401	A10	Human coxsackievirus A10	MF68881 4	7397	-N/A-	Human	A type
402	CV-A10-HB09-035	Human coxsackievirus A10	MT26372	7356	10/2009	Human	A type

			9				
403	CV-A10/HVN11_028_Bac_GiangVNM/2011	Human coxsackievirus A10	LC483986	7386	2011	Human	A type
404	CV-A10/HVN13_016_Hai_PhongVNM/2013	Human coxsackievirus A10	LC483987	7386	2013	Human	A type
405	CV-A10/HVN16_073_Hai_PhongVNM/2016	Human coxsackievirus A10	LC483988	7385	2016	Human	A type
406	CV-CNIC/Guizhou/CHN/14	Human coxsackievirus A10	KT588920	7412	03/07/2014	Human	A type
407	CVA10-FJ-01	Human coxsackievirus A10	KY012321	7412	2014	Human	A type
408	CVA10/Shenzhen10/CHN/2015	Human coxsackievirus A10	KX595290	7411	05/2015	Human	A type
409	CVA10/Shenzhen152/CHN/2013	Human coxsackievirus A10	KX595287	7411	06/2013	Human	A type
410	CVA10/Shenzhen18/CHN/2014	Human coxsackievirus A10	KX595288	7411	04/2014	Human	A type
411	CVA10/Shenzhen180/CHN/2014	Human coxsackievirus A10	KX595289	7411	04/2014	Human	A type
412	GD03	Human coxsackievirus A10	MK791147	7351	08/27/2018	Human	A type
413	HEV792611	Human coxsackievirus A10	MK814855	7411	06/26/2018	Environmental	A type
414	HZ302R/Shandong/China/2014	Human coxsackievirus A10	KY272010	7403	06/06/2014	Human	A type
415	NSW-V23-2007-CVA10	Human coxsackievirus A10	MF678312	6954	2007	Human	A type
416	P148/ZS/CHN/2012	Human coxsackievirus A10	MK645898	7411	04/2012	Human	A type

417	QD102R/Shandong/China/2014	Human coxsackievirus A10	KY272008	7399	04/03/2014	Human	A type
418	R6-19/XY/CHN/2017	Human coxsackievirus A10	MK301475	7356	10/2017	Human	A type
419	TA151R/Shandong/China/2014	Human coxsackievirus A10	KY272009	7337	04/29/2014	Human	A type
420	Human-MK814854	Human coxsackievirus A10	MK814854	7411	06/16/2018	Human	A type
421	USA/2014-23299	Human coxsackievirus A10	MT347977	7411	2014	Human	A type
422	USA/TN/2016-OB2038	Human coxsackievirus A10	KY271944	7243	05/26/2016	Human	A type
423	V6-19/XY/CHN/2017-P1	Human coxsackievirus A10	MK301476	7359	09/2018	Human	A type
424	HEV16087564CA12	Human coxsackievirus A12	MK061425	7396	09/19/2018	Human	A type
425	HEV16537821CA12	Human coxsackievirus A12	MK061424	7396	09/19/2018	Human	A type
426	HEV786663	Human coxsackievirus A12	MH888020	7396	05/03/2018	Human	A type
427	AH17-18/AH/East/CHN/2017-02-12	Human coxsackievirus A16	MT211988	7408	02/12/2017	Human	A type
428	AH18-25/AH/East/CHN/2018-04-26	Human coxsackievirus A16	MT211989	7408	04/26/2018	Human	A type
429	BJ14-3	Human coxsackievirus A16	KU254597	7408	07/09/2014	Human	A type

430	BJ14-4	Human coxsackievirus A16	KU254598	7400	07/09/2014	Human	A type
431	BJ16-68/BJ/Central/CHN/2016-08-01	Human coxsackievirus A16	MT211990	7408	08/01/2016	Human	A type
432	BJ17-14/BJ/Central/CHN/2017-02-15	Human coxsackievirus A16	MT211991	7408	02/15/2017	Human	A type
433	BJ18-58/BJ/Central/CHN/2018-08-20	Human coxsackievirus A16	MT211992	7408	08/20/2018	Human	A type
434	CA16-193	Human coxsackievirus A16	KU854873	7411	2008	Human	A type
435	CA16-194	Human coxsackievirus A16	KX056216	7410	2008	Human	A type
436	CA16-196	Human coxsackievirus A16	KX580041	7410	08/07/2008	Human	A type
437	CQ16-104/CQ/West/CHN/2016-01-13	Human coxsackievirus A16	MT211993	7409	01/13/2016	Human	A type
438	CQ17-38/CQ/West/CHN/2017-04-12	Human coxsackievirus A16	MT211994	7408	04/12/2017	Human	A type
439	CQ18-3/CQ/West/CHN/2018-06-28	Human coxsackievirus A16	MT211995	7409	06/28/2018	Human	A type
440	CV-A16-A01-BLR-IN	Human coxsackievirus A16	KY792576	7410	10/2012	Human	A type
441	CV-A16-A02-BLR-IN	Human coxsackievirus A16	KY792577	7410	10/2012	Human	A type
442	CV-A16-A06-BLR-IN	Human coxsackievirus A16	KY792578	7410	07/2013	Human	A type

443	CV-A16-A10-BLR-IN	Human coxsackievirus A16	KY792579	7410	07/2013	Human	A type
444	CV-A16-A122-BLR-IN	Human coxsackievirus A16	KY792581	7410	06/2015	Human	A type
445	CV-A16-A128-BLR-IN	Human coxsackievirus A16	KY792582	7410	06/2015	Human	A type
446	CV-A16-A13-BLR-IN	Human coxsackievirus A16	KY792580	7407	07/2013	Human	A type
447	CV-A16-M02-BLR-IN	Human coxsackievirus A16	KY792583	7410	04/2013	Human	A type
448	CV-A16-M69-BLR-IN	Human coxsackievirus A16	KY792584	7407	10/2015	Human	A type
449	CV-A16/HVN08.039_HA_GIANGVNM/2008	Human coxsackievirus A16	LC506455	7396	2008	Human	A type
450	CV-A16/HVN08.055_HAI_PHONGVNM/2008	Human coxsackievirus A16	LC506456	7389	2008	Human	A type
451	CV-A16/HVN11.650_THANH_HOAVNM/2011	Human coxsackievirus A16	LC506457	7394	2011	Human	A type
452	CV-A16/HVN13.004_HOA_BINHVNM/2013	Human coxsackievirus A16	LC506458	7374	2013	Human	A type
453	CV-A16/HVN13.005_HAI_PHONGVNM/2013	Human coxsackievirus A16	LC506459	7394	2013	Human	A type
454	CV-A16/HVN14.327_HAI_PHONGVNM/2014	Human coxsackievirus A16	LC506460	7388	2014	Human	A type
455	CV-A16/HVN16.082_HAI_PHONGVNM/2016	Human coxsackievirus A16	LC506461	7298	2016	Human	A type
456	CV-A16/HVN17.120_HAI_PHONGVNM/2017	Human coxsackievirus A16	LC506462	7384	2017	Human	A type
457	CVA16/Shenzhen169/CHN/2017	Human coxsackievirus A16	MH010203	7311	05/2017	Human	A type
458	CVA16/Shenzhen174/CHN/2017	Human coxsackievirus A16	MH010204	7365	05/2017	Human	A type

459	CVA16/Shenzhen179/CHN/2014	Human coxsackievirus A16	KX59529<br>5	7409	04/2014	Human	A type
460	CVA16/Shenzhen189/CHN/2017	Human coxsackievirus A16	MH01020<br>5	7366	05/2017	Human	A type
461	CVA16/Shenzhen220/CHN/2017	Human coxsackievirus A16	MH01020<br>6	7306	05/2017	Human	A type
462	CVA16/Shenzhen289/CHN/2016	Human coxsackievirus A16	MH01020<br>1	7354	06/2016	Human	A type
463	CVA16/Shenzhen36/CHN/2014	Human coxsackievirus A16	KX59529<br>1	7410	05/2014	Human	A type
464	CVA16/Shenzhen469/CHN/2015	Human coxsackievirus A16	MH01019<br>9	7380	10/2015	Human	A type
465	CVA16/Shenzhen500/CHN/2014	Human coxsackievirus A16	MH01019<br>8	7363	12/2014	Human	A type
466	CVA16/Shenzhen73/CHN/2014	Human coxsackievirus A16	KX59529<br>2	7410	06/2014	Human	A type
467	CVA16/Shenzhen74/CHN/2014	Human coxsackievirus A16	KX59529<br>3	7410	06/2014	Human	A type
468	CVA16/Shenzhen76/CHN/2017	Human coxsackievirus A16	MH01020<br>2	7410	03/2017	Human	A type
469	CVA16/Shenzhen79/CHN/2014	Human coxsackievirus A16	KX59529<br>4	7410	07/2014	Human	A type
470	CVA16/Shenzhen87/CHN/2016	Human coxsackievirus A16	MH01020<br>0	7410	04/2016	Human	A type
471	ensh01-CHN-12	Human coxsackievirus A16	KX05853<br>3	7407	2012	Human	A type

472	GD16-105/GD/South/CHN/2016-06-17	Human coxsackievirus A16	MT211996	7409	06/17/2016	Human	A type
473	GD17-81/GD/South/CHN/2017-06-01	Human coxsackievirus A16	MT211997	7408	06/01/2017	Human	A type
474	GD18-104/GD/South/CHN/2018-08-14	Human coxsackievirus A16	MT211998	7408	08/14/2018	Human	A type
475	GDV126	Human coxsackievirus A16	KU163608	7404	2010	Human	A type
476	GS16-110/GS/West/CHN/2016-05-30	Human coxsackievirus A16	MT211999	7409	05/30/2016	Human	A type
477	GS17-636/GS/West/CHN/2017-06-15	Human coxsackievirus A16	MT212000	7411	06/15/2017	Human	A type
478	GS17-658/GS/West/CHN/2017-06-15	Human coxsackievirus A16	MT212001	7411	06/15/2017	Human	A type
479	GS18-179/GS/West/CHN/2018-05-14	Human coxsackievirus A16	MT212002	7408	05/14/2018	Human	A type
480	GZ16-QN035/GZ/South/CHN/2016-06-30	Human coxsackievirus A16	MT212003	7408	06/30/2016	Human	A type
481	HAN17-66/HaN/South/CHN/2017-12-11	Human coxsackievirus A16	MT212004	7408	12/11/2017	Human	A type
482	HAN18-8/HaN/South/CHN/2018-02-28	Human coxsackievirus A16	MT212005	7408	02/28/2018	Human	A type
483	HB16-54015/HB/Central/CHN/2016-01-15	Human coxsackievirus A16	MT212006	7408	01/15/2016	Human	A type
484	HB17-54371/HB/Central/CHN/2017-07-07	Human coxsackievirus A16	MT212007	7409	07/07/2017	Human	A type

485	HB18-20/HB/Central/CHN/2018-05-16	Human coxsackievirus A16	MT212008	7408	05/16/2018	Human	A type
486	HEN17-108/HeN/Central/CHN/2017-06-30	Human coxsackievirus A16	MT212009	7407	06/30/2017	Human	A type
487	HeN18-400/HeN/Central/CHN/2018-07-11	Human coxsackievirus A16	MT212010	7408	07/11/2018	Human	A type
488	HF146/SD/CHN/2008	Human coxsackievirus A16	MG450666	7411	2008	Human	A type
489	HLJ16-HH2016033/HLJ/North/CHN/2016-09-19	Human coxsackievirus A16	MT212011	7408	09/19/2016	Human	A type
490	HLJ18-16/HLJ/North/CHN/2018-08-05	Human coxsackievirus A16	MT212012	7408	08/05/2018	Human	A type
491	HuN16-75/HuN/South/CHN/2016-05-09	Human coxsackievirus A16	MT212013	7408	05/09/2016	Human	A type
492	HUN17-33/HuN/South/CHN/2017-08-07	Human coxsackievirus A16	MT212014	7408	08/07/2017	Human	A type
493	HuN18-5/HuN/South/CHN/2018-02-26	Human coxsackievirus A16	MT212015	7408	02/26/2018	Human	A type
494	JL18-97/JL/North/CHN/2018-08-05	Human coxsackievirus A16	MT212016	7408	08/05/2018	Human	A type
495	JX16-126/JX/East/CHN/2016-12-27	Human coxsackievirus A16	MT212017	7408	12/27/2016	Human	A type
496	JX18-30/JX/East/CHN/2018-03-26	Human coxsackievirus A16	MT212018	7408	03/26/2018	Human	A type
497	K11/YN/CHN/2011	Human coxsackievirus A16	KY425528	7412	06/06/2011	Human	A type

498	K168/8	Human coxsackievirus A16	KY08808<br>4	7411	08/06/2<br>010	Human	A type
499	K34/YN/CHN/2011	Human coxsackievirus A16	KY42553<br>1	7410	05/01/2<br>011	Human	A type
500	KM/M08	Human coxsackievirus A16	MN04620<br>8	7433	11/16/20<br>15	Human	A type
501	L23	Human coxsackievirus A16	KJ746492	7430	2010	Human	A type
502	LN16-23-12/LN/North/CHN/2016-07-11	Human coxsackievirus A16	MT21201<br>9	7409	07/11/20<br>16	Human	A type
503	NSW-V10-2008-CVA16	Human coxsackievirus A16	MF67829<br>9	6950	2008	Human	A type
504	NSW-V24-2008-CVA16	Human coxsackievirus A16	MF67831<br>3	6953	2008	Human	A type
505	NSW-V43-2006-CVA16	Human coxsackievirus A16	MF67833<br>2	6954	2006	Human	A type
506	QH16-8/QH/West/CHN/2016-07-07	Human coxsackievirus A16	MT21202<br>0	7408	07/07/2<br>016	Human	A type
507	QH17-104/QH/West/CHN/2017-10-13	Human coxsackievirus A16	MT21202<br>1	7409	10/13/2<br>017	Human	A type
508	QH18-5/QH/West/CHN/2018-05-12	Human coxsackievirus A16	MT21202<br>2	7410	05/12/2<br>018	Human	A type
509	R141/YN/CHN/2009	Human coxsackievirus A16	KY42553<br>7	7410	06/24/2<br>009	Human	A type
510	R254/YN/CHN/2010	Human coxsackievirus A16	KY42553<br>8	7410	05/18/2<br>010	Human	A type
511	R255/YN/CHN/2010	Human coxsackievirus A16	KY42553	7410	05/18/2	Human	A type

			9		010		
512	R34/YN/CHN/2012	Human coxsackievirus A16	KY42553 4	7410	06/23/2 012	Human	A type
513	R35/YN/CHN/2012	Human coxsackievirus A16	KY42553 3	7410	06/23/2 012	Human	A type
514	R37/YN/CHN/2013	Human coxsackievirus A16	KY42553 5	7398	10/14/2 013	Human	A type
515	R5/YN/CHN/2011	Human coxsackievirus A16	KY42553 0	7412	06/02/2 011	Human	A type
516	R68/YN/CHN/2009	Human coxsackievirus A16	KY42553 2	7409	05/24/2 009	Human	A type
517	S0470B	Human coxsackievirus A16	MN33760 0	7423	-N/A-	Human	A type
518	S1082b	Human coxsackievirus A16	MN33759 3	7588	-N/A-	Human	A type
519	SAX17-50/SaX/Central/CHN/2017-09-16	Human coxsackievirus A16	MT21202 3	7408	09/16/2 017	Human	A type
520	SD16-101/SD/East/CHN/2016-07-22	Human coxsackievirus A16	MT21202 4	7408	07/22/2 016	Human	A type
521	SH-HP-16-51	Human coxsackievirus A16	MG95711 7	7232	10/05/2 016	Human	A type
522	SiICRC01/TH/2012	Human coxsackievirus A16	KX37233 6	7416	08/08/2 012	Human	A type
523	SiICRC01/TH/2014	Human coxsackievirus A16	KX37233 9	7415	07/08/2 014	Human	A type
524	SiICRC05/TH/2011	Human coxsackievirus A16	KX37233	7422	08/15/2	Human	A type

			4		011		
525	SiICRC06/TH/2011	Human coxsackievirus A16	KX37233 5	7418	08/16/2 011	Human	A type
526	SX17-286/SX/Central/CHN/2017-06-16	Human coxsackievirus A16	MT21202 5	7408	06/16/2 017	Human	A type
527	TA271/Shandong/China/2015	Human coxsackievirus A16	MG67482 7	7388	07/18/2 015	Human	A type
528	TJ16-7/TJ/Central/CHN/2016-02-29	Human coxsackievirus A16	MT21202 6	7409	02/29/2 016	Human	A type
529	TJ17-36/TJ/Central/CHN/2017-03-13	Human coxsackievirus A16	MT21202 7	7408	03/13/2 017	Human	A type
530	TJ18-63/TJ/Central/CHN/2018-05-28	Human coxsackievirus A16	MT21202 8	7407	05/28/2 018	Human	A type
531	USA/CT/2016-19518	Human coxsackievirus A16	MF18918 0	7399	11/16/20 16	Human	A type
532	176/GD/CHN/2015	Human coxsackievirus A2	MF28125 7	7363	2015	Human	A type
533	BJ13-53/BJ/CHN/2013	Human coxsackievirus A2	KX15636 0	7400	06/14/2 013	Human	A type
534	CV-A2|41149|RUS|2011	Human coxsackievirus A2	LR027550	4962	-N/A-	Human	A type
535	CV-A2|41963|RUS|2011	Human coxsackievirus A2	LR027549	7347	-N/A-	Human	A type
536	CV-A2|42115|RUS|2011	Human coxsackievirus A2	LR027551	7393	-N/A-	Human	A type
537	CVA2-SHZH13-01	Human coxsackievirus A2	MG21425 7	7400	05/2013	Human	A type
538	CVA2/Shenzhen133/CHN/2013	Human coxsackievirus A2	KX59528 2	7400	05/2013	Human	A type

539	CVA2/Shenzhen143/CHN/2013	Human coxsackievirus A2	KX595283	7400	05/2013	Human	A type
540	CVA2/Shenzhen21/CHN/2015	Human coxsackievirus A2	KX595284	7312	05/2015	Human	A type
541	CVA2/Shenzhen50/CHN/2012	Human coxsackievirus A2	KX595281	7400	09/2012	Human	A type
542	Fleetwood	Human coxsackievirus A2	NC_038306 *	7398	-N/A-	Human	A type
543	HeN13-6/HeN/CHN/2013	Human coxsackievirus A2	KX156350	7400	06/03/2013	Human	A type
544	NSW-V21-2010-CVA2	Human coxsackievirus A2	MF678310	6946	2010	Human	A type
545	NSW-V34-2008-CVA2	Human coxsackievirus A2	MF678322	6946	2008	Human	A type
546	NSW-V44-2008-CVA2	Human coxsackievirus A2	MF678333	6945	2008	Human	A type
547	NSW-V45-2005-CVA2	Human coxsackievirus A2	MF678334	6931	2005	Human	A type
548	NSW-V48-2008-CVA2	Human coxsackievirus A2	MF678338	6944	2008	Human	A type
549	USA/2014-19510	Human coxsackievirus A2	KX810065	7363	01/13/2014	Human	A type
550	Xiangyang 1388	Human coxsackievirus A2	MW846233	7400	12/2017	Human	A type
551	xz028-HEV-1	Human coxsackievirus A2	OL519580	7329	2018	Human	A type

552	01/Taian/SD/2017	Human coxsackievirus A4	MK65883<br>0	7417	08/20/2<br>017	Human	A type
553	06/Taian/SD/2017	Human coxsackievirus A4	MK65883<br>1	7459	09/06/2<br>017	Human	A type
554	1-E9-CA4	Human coxsackievirus A4	KT353722	7337	2008	Human	A type
555	10-108-HaN-2010	Human coxsackievirus A4	MK39106<br>3	7434	04/22/2<br>010	Human	A type
556	10-117-HuN-2010	Human coxsackievirus A4	MK39106<br>4	7422	09/25/2<br>010	Human	A type
557	105/Taian/SD/2017	Human coxsackievirus A4	MK65883<br>2	7440	09/07/2<br>017	Human	A type
558	11-60-CQ-2011	Human coxsackievirus A4	MK39106<br>5	7434	06/06/2<br>011	Human	A type
559	12-119-YN-2012	Human coxsackievirus A4	MK39106<br>6	7434	07/23/2<br>012	Human	A type
560	13-58-JL-2013	Human coxsackievirus A4	MK39106<br>8	7434	08/08/2<br>013	Human	A type
561	13-7-SaX-2013	Human coxsackievirus A4	MK39106<br>7	7434	06/21/2<br>013	Human	A type
562	14-17-BJ-2014	Human coxsackievirus A4	MK39106<br>9	7434	05/23/2<br>014	Human	A type
563	14-29-JS-2014	Human coxsackievirus A4	MK39107<br>0	7434	07/10/2<br>014	Human	A type
564	14-41-JX-2014	Human coxsackievirus A4	MK39107<br>1	7433	05/07/2<br>014	Human	A type
565	15-116-LN-2015	Human coxsackievirus A4	MK39107	7434	06/10/2	Human	A type

			2		015		
566	16-128-JX-2016	Human coxsackievirus A4	MK39107 5	7432	12/22/2 016	Human	A type
567	16-5-HuN-2016	Human coxsackievirus A4	MK39107 3	7424	02/26/2 016	Human	A type
568	16-71-XJ-2016	Human coxsackievirus A4	MK39107 4	7433	06/12/2 016	Human	A type
569	A1/Taian/SD/2018	Human coxsackievirus A4	MK65883 3	7359	06/05/2 018	Human	A type
570	CV-A4/2016/HFMD/104	Human coxsackievirus A4	MN96407 7	7449	2016	Human	A type
571	CV-A4/2016/HFMD/305	Human coxsackievirus A4	MN96407 6	7449	2016	Human	A type
572	CV-A4/2018/herpangina/16	Human coxsackievirus A4	MN96408 2	7450	2018	Human	A type
573	CV-A4/2018/herpangina/25	Human coxsackievirus A4	MN96408 1	7450	2018	Human	A type
574	CV-A4/2018/herpangina/31	Human coxsackievirus A4	MN96408 0	7450	2018	Human	A type
575	CV-A4/2018/herpangina/8	Human coxsackievirus A4	MN96407 8	7450	2018	Human	A type
576	CV-A4/2018/HFMD/792	Human coxsackievirus A4	MN96407 9	7450	2018	Human	A type
577	FY218/QD/CHN/2015	Human coxsackievirus A4	MT78721 9	7426	07/04/2 015	Human	A type
578	HK421778/2014	Human coxsackievirus A4	MH78072	7436	2014	Human	A type

			8				
579	HK422123/2014	Human coxsackievirus A4	MH780725	7417	2014	Human	A type
580	HK422825/2010	Human coxsackievirus A4	MH780727	7441	2010	Human	A type
581	HK423378/2010	Human coxsackievirus A4	MH780726	7425	2010	Human	A type
582	HK436180/2012	Human coxsackievirus A4	MH780724	7438	2012	Human	A type
583	HK452775/2016	Human coxsackievirus A4	MH780729	7439	2016	Human	A type
584	HK458564/2016	Human coxsackievirus A4	MH780730	7440	2016	Human	A type
585	HS144/QD/CHN/2014	Human coxsackievirus A4	MT787220	7416	05/30/2014	Human	A type
586	HS312/QD/CHN/2013	Human coxsackievirus A4	MT787221	7408	08/08/2013	Human	A type
587	HS605/QD/CHN/2014	Human coxsackievirus A4	MT787222	7413	07/17/2014	Human	A type
588	KR110/YN/CHN/2019	Human coxsackievirus A4	MT828545	7436	10/01/2019	Human	A type
589	KR3/YN/CHN/2019	Human coxsackievirus A4	MT828544	7434	10/01/2019	Human	A type
590	MAD-9745-11	Human coxsackievirus A4	LT719044	7414	2011	Human	A type
591	R11-20/YN/CHN/2011	Human coxsackievirus A4	MG431822	7434	2011	Human	A type

592	S270/Changsha/CHN/2019	Human coxsackievirus A4	MT920660	7454	06/25/2019	Human	A type
593	USA/TN/2015-OB2038	Human coxsackievirus A4	KY271949	7378	04/16/2015	Human	A type
594	xz009-HEV-1	Human coxsackievirus A4	OL519574	7372	2018	Human	A type
595	xz012-HEV-1	Human coxsackievirus A4	OL519576	7405	2018	Human	A type
596	xz017-HEV-1	Human coxsackievirus A4	OL519578	7286	2018	Human	A type
597	CV-A5-3487-M14-XY-CHN-2017	Human coxsackievirus A5	MW079817	7424	2017	Human	A type
598	CV-A5|41143|RUS|2011	Human coxsackievirus A5	LR027548	1377	-N/A-	Human	A type
599	CVA5/13164/HUN/2015	Human coxsackievirus A5	KU761262	7405	2015	Human	A type
600	USA/2014-23300	Human coxsackievirus A5	MT081376	7405	2014	Human	A type
601	USA/2014-23301	Human coxsackievirus A5	MT081377	7405	2014	Human	A type
602	Xiangyang 3487	Human coxsackievirus A5	MN663160	7404	12/2017	Human	A type
603	CVA6 -3141290831-FRA-2014	Human coxsackievirus A6	MT814458	7434	05/07/2014	Human	A type
604	CVA6 -3141321081-FRA-2014	Human coxsackievirus A6	MT814556	7434	04/30/2014	Human	A type
605	CVA6 -3141321151-FRA-2014	Human coxsackievirus A6	MT81444	7434	04/11/20	Human	A type

			5		14		
606	CVA6 -3141351171-FRA-2014	Human coxsackievirus A6	MT814504	7434	05/06/2014	Human	A type
607	CVA6 -3141360411-FRA-2014	Human coxsackievirus A6	MT814581	7433	05/10/2014	Human	A type
608	CVA6 -3141390801-FRA-2014	Human coxsackievirus A6	MT814452	7434	05/15/2014	Human	A type
609	CVA6 -3141461291-FRA-2014	Human coxsackievirus A6	MT814591	7434	05/20/2014	Human	A type
610	CVA6 -3141480491-FRA-2014	Human coxsackievirus A6	MT814541	7434	05/23/2014	Human	A type
611	CVA6 -3141480511-FRA-2014	Human coxsackievirus A6	MT814542	7434	05/23/2014	Human	A type
612	CVA6 -3141531301-FRA-2014	Human coxsackievirus A6	MT814550	7434	05/25/2014	Human	A type
613	CVA6 -3141531381-FRA-2014	Human coxsackievirus A6	MT814553	7433	05/30/2014	Human	A type
614	CVA6 -3141541231-FRA-2014	Human coxsackievirus A6	MT814475	7434	05/30/2014	Human	A type
615	CVA6 -3141550561-FRA-2014	Human coxsackievirus A6	MT814593	7434	06/02/2014	Human	A type
616	CVA6 -3141610841-FRA-2014	Human coxsackievirus A6	MT814558	7434	06/04/2014	Human	A type
617	CVA6/S2215/BJ/CHN/2014	Human coxsackievirus A6	MF285639	7433	2014	Human	A type
618	CVA6/S2244/BJ/CHN/2014	Human coxsackievirus A6	MF28564	7434	2014	Human	A type

			0				
619	CVA6/S2307/BJ/CHN/2014	Human coxsackievirus A6	MF28564<br>1	7434	2014	Human	A type
620	CVA6/S2407/BJ/CHN/2014	Human coxsackievirus A6	MF28564<br>2	7433	2014	Human	A type
621	CVA6/S2422/BJ/CHN/2014	Human coxsackievirus A6	MF28564<br>3	7434	2014	Human	A type
622	CVA6/S2534/BJ/CHN/2014	Human coxsackievirus A6	MF28564<br>4	7434	2014	Human	A type
623	CVA6/S2647/BJ/CHN/2014	Human coxsackievirus A6	MF28564<br>5	7433	2014	Human	A type
624	CVA6/S2727/BJ/CHN/2014	Human coxsackievirus A6	MF28564<br>6	7434	2014	Human	A type
625	CVA6/S2770/BJ/CHN/2014	Human coxsackievirus A6	MF28564<br>7	7434	2014	Human	A type
626	CVA6/S2792/BJ/CHN/2014	Human coxsackievirus A6	MF28564<br>8	7434	2014	Human	A type
627	CVA6/S2829/BJ/CHN/2014	Human coxsackievirus A6	MF28564<br>9	7433	2014	Human	A type
628	CVA6/S2859/BJ/CHN/2014	Human coxsackievirus A6	MF28565<br>0	7434	2014	Human	A type
629	CVA6/S2870/BJ/CHN/2014	Human coxsackievirus A6	MF28565<br>1	7434	2014	Human	A type
630	DY081/SD/CHN/2015	Human coxsackievirus A6	MK10620<br>6	7434	2015	Human	A type
631	F106/YN/CHN/2016	Human coxsackievirus A6	MN84579	7434	2016	Human	A type

			7				
632	F141/YN/CHN/2016	Human coxsackievirus A6	MK10621 2	7434	2016	Human	A type
633	F173/YN/CHN/2016	Human coxsackievirus A6	MK10621 3	7435	2016	Human	A type
634	F219/YN/CHN/2016	Human coxsackievirus A6	MN84578 1	7434	2016	Human	A type
635	F220/YN/CHN/2016	Human coxsackievirus A6	MN84579 8	7434	2016	Human	A type
636	F224/YN/CHN/2016	Human coxsackievirus A6	MN84578 2	7434	2016	Human	A type
637	F274/YN/CHN/2016	Human coxsackievirus A6	MN84579 9	7434	2016	Human	A type
638	F92/YN/CHN/2016	Human coxsackievirus A6	MN84579 6	7435	2016	Human	A type
639	FH117/SX/CHN/2013	Human coxsackievirus A6	MK10619 5	7435	2013	Human	A type
640	FH233/SX/CHN/2013	Human coxsackievirus A6	MK10619 6	7436	2013	Human	A type
641	GD246/2011	Human coxsackievirus A6	KX18917 8	7391	2011	Human	A type
642	15NHP108	Human enterovirus 71	LC627072	7390	2015	Human	A type
643	15NHP120	Human enterovirus 71	LC627073	7400	2015	Human	A type
644	15NHP128	Human enterovirus 71	LC627074	7399	2015	Human	A type
645	15NHP152	Human enterovirus 71	LC627075	7399	2015	Human	A type

646	15NHP157	Human enterovirus 71	LC627076	7402	2015	Human	A type
647	15NHP226	Human enterovirus 71	LC627077	7397	2015	Human	A type
648	15|VSV286032|FRA16	Human enterovirus 71	LR027536	7411	-N/A-	Human	A type
649	160-50	Human enterovirus 71	MG875331	7404	2016	Human	A type
650	16L	Human enterovirus 71	MN515037	7368	2016	Human	A type
651	16NHP373	Human enterovirus 71	LC627078	7397	2016	Human	A type
652	16NHP379	Human enterovirus 71	LC627086	7395	2016	Human	A type
653	16NHP391	Human enterovirus 71	LC627087	7395	2016	Human	A type
654	16NHP399	Human enterovirus 71	LC627079	7402	2016	Human	A type
655	16NHP417	Human enterovirus 71	LC627088	7394	2016	Human	A type
656	16NHP436	Human enterovirus 71	LC627089	7398	2016	Human	A type
657	16NHP442	Human enterovirus 71	LC627090	7397	2016	Human	A type
658	16NHP450	Human enterovirus 71	LC627081	7404	2016	Human	A type
659	16NHP453	Human enterovirus 71	LC627082	7401	2016	Human	A type
660	16NHP455	Human enterovirus 71	LC627091	7392	2016	Human	A type
661	16NHP456	Human enterovirus 71	LC627083	7410	2016	Human	A type
662	16NHP462	Human enterovirus 71	LC627084	7402	2016	Human	A type
663	16NHP475	Human enterovirus 71	LC627092	7394	2016	Human	A type
664	16|VSV286034|FRA16	Human enterovirus 71	LR027537	7411	-N/A-	Human	A type
665	17|VSV286035|FRA16	Human enterovirus 71	LR027535	7411	-N/A-	Human	A type

666	1859-Yamagata-2012	Human enterovirus 71	LC626879	7402	2012	Human	A type
667	1888-Yamagata-2012	Human enterovirus 71	LC626878	7402	2012	Human	A type
668	18|VSV286036|FRA16	Human enterovirus 71	LR027538	7411	-N/A-	Human	A type
669	1934-Yamagata-2003	Human enterovirus 71	LC626896	7404	2003	Human	A type
670	2000-Yamagata-2012	Human enterovirus 71	LC626900	7402	2012	Human	A type
671	2005-1480|NLD05	Human enterovirus 71	LR027541	6773	-N/A-	Human	A type
672	2015g03	Human enterovirus 71	KY612315	7404	01/2015	Human	A type
673	2019-EV-A71-R398	Human enterovirus 71	MT708799	7410	2019	Human	A type
674	2019-EV-A71-R400	Human enterovirus 71	MT708800	7410	2019	Human	A type
675	2019-EV-A71-R405	Human enterovirus 71	MT708804	7406	2019	Human	A type
676	2019-EV-A71-R431	Human enterovirus 71	MT708805	7406	2019	Human	A type
677	2019-EV-A71-R477	Human enterovirus 71	MT708801	7410	2019	Human	A type
678	2019-EV-A71-R480	Human enterovirus 71	MT708802	7410	2019	Human	A type
679	2019-EV-A71-R798	Human enterovirus 71	MT708803	7410	2019	Human	A type
680	2065-Yamagata-2004	Human enterovirus 71	LC506516	7434	2004	Human	A type
681	2317-Yamagata-2006	Human enterovirus 71	LC626895	7396	2006	Human	A type

682	23|TOU307036|FRA16	Human enterovirus 71	LR027532	7411	-N/A-	Human	A type
683	2488-Yamagata-2006	Human enterovirus 71	LC626894	7395	2006	Human	A type
684	2498-Yamagata-2006	Human enterovirus 71	LC626893	7393	2006	Human	A type
685	25|AMI302001|FRA16	Human enterovirus 71	LR027546	7410	-N/A-	Human	A type
686	25|AMI302002|FRA16	Human enterovirus 71	LR027531	7409	-N/A-	Human	A type
687	26|PMB501259|FRA17	Human enterovirus 71	LR027539	7413	-N/A-	Human	A type
688	2716-Yamagata-03	Human enterovirus 71	LC375766	7434	2003	Human	A type
689	2779-Yamagata-2002	Human enterovirus 71	LC626892	7401	2002	Human	A type
690	30-2/2015/BJ	Human enterovirus 71	MG214681	7409	04/2015	Human	A type
691	TW-70389-2008	Human enterovirus 71	MG756708	7394	2008	Human	A type
692	TW-70521-2005	Human enterovirus 71	MG756702	7387	2005	Human	A type
693	TW-70751-2005	Human enterovirus 71	MG756703	7388	2005	Human	A type
694	TW-70954-2008	Human enterovirus 71	MG756709	7394	2008	Human	A type
695	TW-71215-2011	Human enterovirus 71	MG756722	7388	2011	Human	A type
696	TW-71379-2008	Human enterovirus 71	MG756710	7394	2008	Human	A type
697	TW-71650-2011	Human enterovirus 71	MG756723	7394	2011	Human	A type

698	TW-71680-2008	Human enterovirus 71	MG756711	7394	2008	Human	A type
699	TW-72031-2008	Human enterovirus 71	MG756712	7394	2008	Human	A type
700	TW-72043-2007	Human enterovirus 71	MG756705	7391	2007	Human	A type
701	TW-72219-2008	Human enterovirus 71	MG756713	7394	2008	Human	A type
702	TW-72523-2008	Human enterovirus 71	MG756714	7394	2008	Human	A type
703	TW-96050-2010	Human enterovirus 71	MG756719	7376	2010	Human	A type
704	TW-96102-2005	Human enterovirus 71	MG756704	7387	2005	Human	A type
705	USA/2018-23092	Human enterovirus 71	MK652139	7389	11/2018	Human	A type
706	USA/2018-23124	Human enterovirus 71	MK800119	7335	2018	Human	A type
707	USA/2018-23296	Human enterovirus 71	MT081373	7410	2018	Human	A type
708	USA/2018-23297	Human enterovirus 71	MT081374	7408	2018	Human	A type
709	USA/2018-23298	Human enterovirus 71	MT081375	7408	2018	Human	A type
710	USA/AK/2016-19516	Human enterovirus 71	MG976581	7385	2016	Human	A type

711	USA/CO/2018-23026	Human enterovirus 71	MH718269	6798	2018	Human	A type
712	wh064-CHN-2010	Human enterovirus 71	MN747117	7405	2010	Human	A type
713	wh170-CHN-2012	Human enterovirus 71	MN747118	7405	2012	Human	A type
714	Y90-3896	Human enterovirus 71	LC506514	7434	1990	Human	A type
715	SiICRC01/TH/2012	Human enterovirus 71	KX372314	7416	08/17/2012	Human	A type
716	SiICRC01/TH/2013	Human enterovirus 71	KX372323	7417	02/26/2013	Human	A type
717	SiICRC02/TH/2012	Human enterovirus 71	KX372315	7418	07/23/2012	Human	A type
718	SiICRC03/TH/2012	Human enterovirus 71	KX372316	7418	10/02/2012	Human	A type
719	SiICRC04/TH/2012	Human enterovirus 71	KX372317	7422	07/26/2012	Human	A type
720	SiICRC05/TH/2012	Human enterovirus 71	KX372318	7417	08/28/2012	Human	A type
721	SiICRC05/TH/2013	Human enterovirus 71	KX372327	7417	11/01/2013	Human	A type
722	SiICRC05/TH/2014	Human enterovirus 71	KX372329	7418	02/12/2014	Human	A type
723	SiICRC06/TH/2012	Human enterovirus 71	KX372319	7419	09/12/2012	Human	A type
724	SiICRC07/TH/2014	Human enterovirus 71	KX37233	7421	07/07/2	Human	A type

			1		014		
725	SiICRC08/TH/2011	Human enterovirus 71	KX37231 0	7418	08/10/2 011	Human	A type
726	SiICRC09/TH/2012	Human enterovirus 71	KX37232 0	7414	10/01/2 012	Human	A type
727	SiICRC10/TH/2011	Human enterovirus 71	KX372311	7427	08/15/2 011	Human	A type
728	SiICRC10/TH/2012	Human enterovirus 71	KX37232 1	7420	11/01/20 12	Human	A type
729	SiICRC11/TH/2012	Human enterovirus 71	KX37232 2	7417	10/01/2 012	Human	A type
730	SiICRC15/TH/2011	Human enterovirus 71	KX37231 2	7430	08/10/2 011	Human	A type
731	STU562356|DEU03	Human enterovirus 71	LR027542	7410	-N/A-	Human	A type
732	10-2188-2	Human enterovirus 76	MH11802 8	7423	2010	Human	A type
733	10-3291-2	Human enterovirus 76	MH11802 9	7586	2010	Human	A type
734	11-1302-2	Human enterovirus 76	MH11803 0	7414	2011	Human	A type
735	17-1936-1_A76	Human enterovirus 76	MH11802 4	7378	2017	Human	A type
736	M19s (P2)	Simian enterovirus 19	AF326754	7373	-N/A-	Human	A type
737	RNM5	Simian enterovirus 46	EF667343	7395	-N/A-	Human	A type
738	A108/YN/CHN/2009	Coxsackievirus A9	KM89027 7	7424	07/06/2 009	Human	B type

739	A242/YN/CHN/2009	Coxsackievirus A9	KM890278	7446	09/18/2009	Human	B type
740	CV-A9/P220/2013/China	Coxsackievirus A9	KP290111	7460	2013	Human	B type
741	CV-A9/P56/2013/China	Coxsackievirus A9	KP289434	7448	2013	Human	B type
742	Griggs	Coxsackievirus A9	D00627	7452	-N/A-	Human	B type
743	LHY03	Coxsackievirus A9	KP266574	7405	04/25/2010	Human	B type
744	B1/Groningen/2011	Coxsackievirus B1	LN854562	7410	2011	Human	B type
745	CVB1SD2011CHN	Coxsackievirus B1	JX976769	7368	07/2011	Human	B type
746	MSH/KM9/2009	Coxsackievirus B1	JN596588	7384	10/2009	Human	B type
747	Human-M16560	Coxsackievirus B1	M16560	7389	-N/A-	Human	B type
748	KOR 04-243	Coxsackievirus B2	EF174468	7411	-N/A-	Human	B type
749	KOR 04-279	Coxsackievirus B2	EF174469	7411	-N/A-	Human	B type
750	Ohio	Coxsackievirus B2	AF081485	7403	-N/A-	Human	B type
751	31-1-93	Coxsackievirus B3	AF231763	7400	-N/A-	Human	B type
752	A103/KM/09	Coxsackievirus B3	JX843810	7389	07/20/2009	Human	B type
753	AH30	Coxsackievirus B3	KC481610	7399	11/2011	Human	B type
754	B3	Coxsackievirus B3	U57056	7400	-N/A-	Human	B type
755	Beijing0811	Coxsackievirus B3	GQ141875	7402	11/2008	Human	B type

756	Nancy	Coxsackievirus B3	JN048468	7415	-N/A-	Human	B type
757	Nancy	Coxsackievirus B3	JX312064	7399	10/01/2<br>008	Human	B type
758	Nancy	Coxsackievirus B3	M16572	7396	-N/A-	Human	B type
759	SSM-CVB3	Coxsackievirus B3	GU10948<br>1	7397	2006	Monkey	B type
760	Human-M33854	Coxsackievirus B3	M33854	7399	-N/A-	Human	B type
761	Human-M88483	Coxsackievirus B3	M88483	7399	-N/A-	Human	B type
762	BM24G/NM/CHN/2010	Coxsackievirus B4	KF781525	7394	2010	Human	B type
763	CV-B4/P11/2013/China	Coxsackievirus B4	KP28943<br>3	7402	2013	Human	B type
764	E2 variant	Coxsackievirus B4	AF311939	7397	-N/A-	Human	B type
765	HHHT34T/NM/CHN/2010	Coxsackievirus B4	KF781524	7393	2010	Human	B type
766	J.V.B. Benschoten	Coxsackievirus B4	X05690	7395	-N/A-	Human	B type
767	Tuscany	Coxsackievirus B4	DQ48042<br>0	7395	-N/A-	Human	B type
768	03001N	Coxsackievirus B5	JX017383	7408	2011	Human	B type
769	17Y	Coxsackievirus B5	JX017382	7404	2011	Human	B type
770	1954/85/UK	Coxsackievirus B5	X67706	7402	-N/A-	Human	B type
771	19CSF	Coxsackievirus B5	JX017381	7402	2011	Human	B type
772	2000/CSF/KOR	Coxsackievirus B5	AY875692	7403	-N/A-	Human	B type
773	CV-B5/P727/2013/China	Coxsackievirus B5	KP28943<br>8	7400	2013	Human	B type

774	CVB5/CC10/10	Coxsackievirus B5	JN580070	7402	2010	Human	B type
775	CVB5/CC10/16	Coxsackievirus B5	JN695050	6558	2010	Human	B type
776	CVB5/CC10/17	Coxsackievirus B5	JN695051	6558	2010	Human	B type
777	CVB5/SD/09	Coxsackievirus B5	JX276378	7399	05/02/2009	Human	B type
778	Faulkner	Coxsackievirus B5	AF114383	7400	-N/A-	Human	B type
779	LEV15	Coxsackievirus B6	JQ041368	7340	2011	Human	B type
780	Schmitt	Coxsackievirus B6	AF105342	7398	-N/A-	Human	B type
781	Schmitt	Coxsackievirus B6	AF114384	7397	-N/A-	Human	B type
782	Schmitt (1-15-21)	Coxsackievirus B6	AF039205	7398	-N/A-	Human	B type
783	E1/TO/BR/069	Echovirus E1	MW775345	7419	09/13/2014	Human	B type
784	01/GZ/CHN/2019	Echovirus E11	MW883610	7392	2019	Human	B type
785	02/GZ/CHN/2019	Echovirus E11	MW883611	7392	2019	Human	B type
786	03/GZ/CHN/2019	Echovirus E11	MW883612	7393	2019	Human	B type
787	04/GZ/CHN/2019	Echovirus E11	MW883613	7392	2019	Human	B type
788	05/GZ/CHN/2019	Echovirus E11	MW883614	7392	2019	Human	B type
789	1000/ISR/1999	Echovirus E11	KY981561	7412	1999	Human	B type

790	5824/ISR/1992	Echovirus E11	KY981570	7431	1992	Human	B type
791	6067/ISR/1993	Echovirus E11	KY981572	7419	1993	Human	B type
792	8416/ISR/1997	Echovirus E11	KY981577	7422	05/06/1997	Human	B type
793	9310/ISR/1998	Echovirus E11	KY981580	7432	02/19/1998	Human	B type
794	9368/ISR/1998	Echovirus E11	KY981581	7431	04/01/1998	Human	B type
795	D207	Echovirus E11	EF634316	7436	-N/A-	Human	B type
796	K1529/YN/CHN/2013	Echovirus E12	MF083154	7417	06/14/2013	Human	B type
797	K605/YN/CHN/2013	Echovirus E12	MF083152	7421	06/02/2013	Human	B type
798	K624/YN/CHN/2013	Echovirus E12	MF083153	7424	06/06/2013	Human	B type
799	Human-X77708	Echovirus E12	X77708	7501	-N/A-	Human	B type
800	wildtype	Echovirus E12	X79047	7501	-N/A-	Human	B type
801	Del Carmen	Echovirus E13	AY302539	7410	-N/A-	Human	B type
802	E13/Spain_LCR53/2016	Echovirus E13	MZ389224	7366	05/2016	Human	B type
803	E14/P843/2013/China	Echovirus E14	KP289440	7467	2013	Human	B type
804	E14/P968/2013/China	Echovirus E14	KP289441	7451	2013	Human	B type

805	RO-81-1-79	Echovirus E14	LS451299	7450	1979	Human	B type
806	Tow	Echovirus E14	AY302540	7450	-N/A-	Human	B type
807	CH 96-51	Echovirus E15	AY302541	7437	-N/A-	Human	B type
808	E16/P85/2013/China	Echovirus E16	KP28943 6	7433	2013	Human	B type
809	Harrington	Echovirus E16	AY302542	7437	-N/A-	Human	B type
810	CHHE-29	Echovirus E17	AY302543	7416	-N/A-	Human	B type
811	12G5	Echovirus E18	MN74914 3	7412	2015	Human	B type
812	12J3	Echovirus E18	MN74914 6	7422	2015	Human	B type
813	A83/YN/CHN/2016	Echovirus E18	KY82885 1	7404	07/21/2 016	Human	B type
814	A86/YN/CHN/2016	Echovirus E18	KY82885 2	7399	07/12/2 016	Human	B type
815	BJ2019-S7323	Echovirus E18	MN81581 2	7413	2019	Human	B type
816	E18-398/HeB/CHN/2015	Echovirus E18	MG72025 9	7414	08/2015	Human	B type
817	E18-HeB15-54462/HeB/CHN/2015	Echovirus E18	MG72026 0	7414	07/2015	Human	B type
818	E18-HeB15-54498/HeB/CHN/2015	Echovirus E18	MG72026 1	7414	07/2015	Human	B type
819	E18/JXY2-2/2019	Echovirus E18	MT35022 4	7414	2019	Human	B type

820	Jena/VI10227/10	Echovirus E18	KX139447	7424	11/19/2010	Human	B type
821	PC06/JS/CHN/2019	Echovirus E18	MT755385	7414	06/2019	Human	B type
822	USA/2015/CA-RGDS-1049	Echovirus E18	MN166092	7412	07/31/2015	Human	B type
823	Burke	Echovirus E19	AY302544	7433	-N/A-	Human	B type
824	HAI/2016-23039B	Echovirus E19	MK000148	7417	04/20/2016	Human	B type
825	HAI/2016-23052	Echovirus E19	MK000191	7417	07/27/2016	Human	B type
826	HAI/2017-23079	Echovirus E19	MK000216	7417	01/26/2017	Human	B type
827	NGR_2014	Echovirus E19	MH745407	7426	2014	Human	B type
828	PDV_BLR_IN	Echovirus E19	KY792585	7437	07/1996	Human	B type
829	Cornelis	Echovirus E2	AF465518	7435	-N/A-	Human	B type
830	Cornelis	Echovirus E2	AY302545	7435	-N/A-	Human	B type
831	USA/2013-19511	Echovirus E2	KX810066	7364	06/20/2013	Human	B type
832	812/YN/CHN/2010	Echovirus E20	KX060810	7398	05/2010	Human	B type
833	JV-1	Echovirus E20	AY302546	7394	-N/A-	Human	B type
834	KM-EV20-2010	Echovirus E20	KF812551	7395	05/20/2010	Human	B type

835	553/YN/CHN/2013	Echovirus E21	MN481500	7426	05/20/2013	Human	B type
836	Farina	Echovirus E21	AY302547	7426	-N/A-	Human	B type
837	13-2053-1_E24	Echovirus E24	MH144604	6681	2013	Human	B type
838	DeCamp	Echovirus E24	AY302548	7433	-N/A-	Human	B type
839	PZ18G/JS/20120703	Echovirus E24	KP036484	7373	-N/A-	Human	B type
840	10-4339-2	Echovirus E25	MH118026	7376	2010	Human	B type
841	E25/2010/CHN/BJ	Echovirus E25	KJ957190	7429	08/2010	Human	B type
842	E25/ZE-wly/Zhejiang/CHN/2005	Echovirus E25	KX774483	7439	2005	Human	B type
843	E25SD2010CHN	Echovirus E25	JX976772	7420	07/2010	Human	B type
844	USA/2018-23126	Echovirus E25	MK800121	7212	2018	Human	B type
845	USA/CA/RGDS-2017-1010	Echovirus E25	MK532311	7281	06/2017	Human	B type
846	XM0297	Echovirus E25	KP099941	7428	2013	Human	B type
847	Coronel	Echovirus E26	AY302550	7424	-N/A-	Human	B type
848	Bacon	Echovirus E27	AY302551	7412	-N/A-	Human	B type
849	E29/TO/BR/193	Echovirus E29	MW775347	7418	10/22/2015	Human	B type
850	Human/E29/BRA/PA-29/BRA/2014	Echovirus E29	MK68907	7410	2014	Human	B type

			0				
851	JV-10	Echovirus E29	AY302552	7427	-N/A-	Human	B type
852	123-R2	Echovirus E3	MK791150	7414	11/14/2018	Human	B type
853	2-E6-TW	Echovirus E3	KT353723	7328	2008	Human	B type
854	Env_2016_Sep_E-3	Echovirus E3	MG451804	7303	09/08/2016	Human	B type
855	OC10-774	Echovirus E3	AB647325	7428	10/2010	Human	B type
856	OC10-798	Echovirus E3	AB647326	7428	11/2010	Human	B type
857	PicoBank/DM1/E3	Echovirus E3	AJ849942	7427	-N/A-	Human	B type
858	1-B4-TW	Echovirus E30	KT353720	7190	2008	Human	B type
859	13-311	Echovirus E30	KY888272	7428	2013	Human	B type
860	13-759	Echovirus E30	KY888273	7426	2013	Human	B type
861	E30/Spain_LCR675/2017	Echovirus E30	MZ389232	7431	03/2017	Human	B type
862	E30/TO/BR/032	Echovirus E30	MW775344	7465	04/25/2014	Human	B type
863	E30SD2010CHN	Echovirus E30	JX976773	7425	07/2010	Human	B type
864	Echo30/Hokkaido.JPN/21208/2017	Echovirus E30	LC416533	7428	08/31/2017	Human	B type
865	Echo30/Hokkaido.JPN/21326/2017	Echovirus E30	LC416536	7428	11/24/20	Human	B type

					17		
866	Caldwell	Echovirus E31	AY302554	7432	-N/A-	Human	B type
867	Pr-10	Echovirus E32	AY302555	7420	-N/A-	Human	B type
868	Toluca-3	Echovirus E33	AY302556	7394	-N/A-	Human	B type
869	YNA12/CHN/2013	Echovirus E33	KU379646	7392	09/19/2013	Human	B type
870	YNK35/CHN/2013	Echovirus E33	KT965725	7392	10/15/2013	Human	B type
871	2F5	Echovirus E4	MF554740	7288	-N/A-	Human	B type
872	AUS250G	Echovirus E4	FJ172447	7384	10/22/2007	Human	B type
873	Pesacek	Echovirus E4	AY302557	7394	-N/A-	Human	B type
874	Kor06-ECV5-253cn	Echovirus E5	HM775882	7430	07/2006	Human	B type
875	Noyce	Echovirus E5	AF083069	7433	-N/A-	Human	B type
876	2-C2	Echovirus E6	KT353724	7018	2008	Human	B type
877	2-D5-TW	Echovirus E6	KT353725	7018	2008	Human	B type
878	2005-29-1	Echovirus E6	KP266568	7383	12/12/2009	Human	B type
879	E6/Spain_LCR365/2015	Echovirus E6	MZ389227	7352	05/2015	Human	B type
880	Echo6/Hokkaido.JPN/19722/2011	Echovirus E6	AB705309	7419	09/02/2011	Human	B type

881	NSW-V18-2007-ECHO6	Echovirus E6	MF678307	6902	2007	Human	B type
882	RA/E6/Ahvaz/Iran/2011	Echovirus E6	KX619440	7456	2011	Human	B type
883	07VI447	Echovirus E7	MH043136	7441	01/2007	Human	B type
884	2001-031	Echovirus E7	KP266569	7426	11/21/2001	Human	B type
885	2001-31	Echovirus E7	KP266570	7426	11/21/2001	Human	B type
886	DH22G/JS/2012	Echovirus E7	KJ765699	7393	2012	Human	B type
887	Env_2016_Sep_E-7a	Echovirus E7	MG451805	7196	09/08/2016	Human	B type
888	NSW-V35A-2006-ECHO7	Echovirus E7	MF678323	6911	2006	Human	B type
889	NSW-V36-2012-ECHO7	Echovirus E7	MF678325	6918	2012	Human	B type
890	UMMC	Echovirus E7	AY036578	7426	-N/A-	Human	B type
891	Barty	Echovirus E9	AF524866	7453	-N/A-	Human	B type
892	DM	Echovirus E9	AF524867	7453	-N/A-	Human	B type
893	DM14	Echovirus E9	KC238669	7453	-N/A-	Human	B type
894	DM33	Echovirus E9	KC238667	7453	-N/A-	Human	B type
895	DM34	Echovirus E9	KC238668	7453	-N/A-	Human	B type

896	NSW-V47A-2005-ECHO9	Echovirus E9	MF678336	6944	2005	Human	B type
897	NSW-V54-2005-ECHO9	Echovirus E9	MF678344	7005	2005	Human	B type
898	UW1	Echovirus E9	KX610685	7464	06/2016	Human	B type
899	BAN2000-10500	Enterovirus B100	DQ902713	7421	-N/A-	Human	B type
900	CIV03-10361	Enterovirus B101	AY843308	7445	-N/A-	Human	B type
901	148/YN/CHN/12	Enterovirus B106	KF990476	7420	01/01/2012	Human	B type
902	TN94-0349	Enterovirus B107	AB426609	7423	-N/A-	Human	B type
903	Q0011/XZ/CHN/2000	Enterovirus B111	KF312882	7428	09/22/2000	Human	B type
904	Toluca-1	Enterovirus B69	AY302560	7411	-N/A-	Human	B type
905	088/SD/CHN/04	Enterovirus B73	KF874626	7412	2004	Human	B type
906	CA55-1988	Enterovirus B73	AF241359	7411	-N/A-	Human	B type
907	TO-127	Enterovirus B73	MK069966	6993	2015	Human	B type
908	Rikaze-136/XZ/CHN/2010	Enterovirus B74	JQ397329	7407	12/08/2010	Human	B type
909	USA/CA75-10213	Enterovirus B74	AY556057	7410	-N/A-	Human	B type
910	102/SD/CHN/97	Enterovirus B75	KF874627	7426	1997	Human	B type
911	USA/OK85-10362	Enterovirus B75	AY556070	7429	-N/A-	Human	B type

912	Y16/XZ/CHN/2007	Enterovirus B75	MW183133	7425	2007	Human	B type
913	Y20/XZ/CHN/2007	Enterovirus B75	MW183136	7425	2007	Human	B type
914	Y24/XZ/CHN/2007	Enterovirus B75	MW183137	7425	2007	Human	B type
915	Y25/XZ/CHN/2007	Enterovirus B75	MW183138	7425	2007	Human	B type
916	Y26/XZ/CHN/2007	Enterovirus B75	MW183139	7425	2007	Human	B type
917	CF496-99	Enterovirus B77	AJ493062	7415	-N/A-	Human	B type
918	USA/TX97-10394	Enterovirus B77	AY843302	7422	-N/A-	Human	B type
919	NH95-0601	enterovirus B79	AB426610	7427	-N/A-	Human	B type
920	HT-LYKH203F/XJ/CHN/2011	Enterovirus B80	MH614922	7464	2011	Human	B type
921	HT-TSLH64F/XJ/CHN/2011	Enterovirus B80	MH614923	7464	2011	Human	B type
922	HTYT-XBBZH73F/XJ/CHN/2011	Enterovirus B80	MH614924	7464	2011	Human	B type
923	HZ01/SD/CHN/2004	Enterovirus B80	JX644073	7464	06/01/2004	Human	B type
924	KOUAN67/XZ/CHN/2010	Enterovirus B80	MH614925	7463	2010	Human	B type
925	USA/CA67-10387	Enterovirus B80	AY843298	7428	-N/A-	Human	B type
926	99279/XZ/CHN/1999	Enterovirus B81	KJ755189	7417	10/10/1	Human	B type

					999		
927	99298c/XZ/CHN/1999	Enterovirus B81	KJ755190	7417	10/10/1 999	Human	B type
928	USA/CA68-10389	Enterovirus B81	AY843299	7415	-N/A-	Human	B type
929	USA/CA64-10390	Enterovirus B82	AY843300	7427	-N/A-	Human	B type
930	246/YN/CHN/08	Enterovirus B83	KU70790 2	7394	2008	Human	B type
931	99245/XZ/CHN/1999	Enterovirus B83	MN16468 3	7396	09/17/1 999	Human	B type
932	99258/XZ/CHN/1999	Enterovirus B83	MN16468 4	7396	09/05/1 999	Human	B type
933	99267/XZ/CHN/1999	Enterovirus B83	MN16468 5	7396	09/06/1 999	Human	B type
934	AFP341-GD-CHN-2001	Enterovirus B83	MN59745 3	7396	08/18/2 001	Human	B type
935	USA/CA76-10392	Enterovirus B83	AY843301	7394	-N/A-	Human	B type
936	AFP452/GD/CHN/2004	Enterovirus B84	KP26205 3	7421	10/05/2 004	Human	B type
937	CIV2003-10603	Enterovirus B84	DQ90271 2	7423	-N/A-	Human	B type
938	BAN00-10353	Enterovirus B85	AY843303	7418	-N/A-	Human	B type
939	HT-LYKH202F/XJ/CHN/2011	Enterovirus B85	JX898908	7423	09/01/2 011	Human	B type
940	HTPS-MJH21F/XJ/CHN/2011	Enterovirus B85	JX898906	7423	09/06/2 011	Human	B type

941	HTPS-MKLH04F/XJ/CHN/2011	Enterovirus B85	JX898907	7424	09/06/2011	Human	B type
942	HTYT-ARL-AFP02F/XJ/CHN/2011	Enterovirus B85	JX898909	7423	08/30/2011	Human	B type
943	HTYT-ARLH403F/XJ/CHN/2011	Enterovirus B85	JX898905	7422	08/29/2011	Human	B type
944	BAN00-10354	Enterovirus B86	AY843304	7400	-N/A-	Human	B type
945	BAN01-10396	Enterovirus B87	AY843305	7422	-N/A-	Human	B type
946	LY02/SD/CHN/2000	Enterovirus B87	KC292019	7423	01/01/2000	Human	B type
947	11-1899-1	Enterovirus B88	MH144601	7421	2011	Human	B type
948	11-4644-1	Enterovirus B88	MH118025	7427	2011	Human	B type
949	12-008-2_E88	Enterovirus B88	MH144607	7408	2012	Human	B type
950	BAN01-10398	Enterovirus B88	AY843306	7433	-N/A-	Human	B type
951	NIV-17-3150-2	Enterovirus B88	MG982664	7369	2017	Human	B type
952	99188/SD/CHN/1999/EV97	Enterovirus B97	GU550508	7394	08/27/1999	Human	B type
953	BAN99-10355	Enterovirus B97	AY843307	7413	-N/A-	Human	B type
954	DT94-0227	Enterovirus B97	AB426611	7413	-N/A-	Human	B type
955	T92-1499	Enterovirus B98	AB426608	7422	-N/A-	Human	B type

956	1-D7-CA9	Human coxsackievirus A9	KT353721	7219	2008	Human	B type
957	16C2	Human coxsackievirus A9	KY67497<br>4	7387	2016	Human	B type
958	NSW-V14-2009-CVA9	Human coxsackievirus A9	MF67830<br>3	6931	2009	Human	B type
959	NSW-V20-2008-CVA9	Human coxsackievirus A9	MF67830<br>9	7278	2008	Human	B type
960	USA/MI/2005-23029	Human coxsackievirus A9	MH75298<br>7	7408	2005	Human	B type
961	xz018-HEV-1	Human coxsackievirus A9	OL51957<br>9	7396	2018	Human	B type
962	CVB1/XM0108	Human coxsackievirus B1	MG78041<br>4	7371	2011	Human	B type
963	13-2380-2_B2	Human coxsackievirus B2	MH14460<br>0	7389	2013	Human	B type
964	NSW-V06-2008-CVB2	Human coxsackievirus B2	MF67829<br>5	6889	2008	Human	B type
965	NSW-V52-2008-CVB2	Human coxsackievirus B2	MF67834<br>2	6910	2008	Human	B type
966	NSW-V53-2010-CVB2	Human coxsackievirus B2	MF67834<br>3	6919	2010	Human	B type
967	RW41-2/YN/CHN/2012	Human coxsackievirus B2	KX49953<br>6	7405	07/19/2<br>012	Human	B type
968	08TC170	Human coxsackievirus B3	KR36287<br>8	7401	2008	Human	B type
969	37012	Human coxsackievirus B3	MK79114	7400	05/01/2	Human	B type

			8		001		
970	CB3/2035A	Human coxsackievirus B3	KY28652 9	7388	2008	Human	B type
971	DH16G/JS/2012	Human coxsackievirus B3	KP03648 0	7390	-N/A-	Human	B type
972	Env_2016_Sep_CV-B3	Human coxsackievirus B3	MG45180 2	7306	09/08/2 016	Human	B type
973	LRY007	Human coxsackievirus B3	KX98198 7	7388	02/11/20 14	Human	B type
974	XZ2011028	Human coxsackievirus B3	MH83632 3	7399	2011	Human	B type
975	2019/FR/3996	Human coxsackievirus B4	MN59027 3	7203	03/31/2 019	Human	B type
976	B4_C08-219	Human coxsackievirus B4	JX417724	7394	2008	Human	B type
977	Env_2017_Jan_CV-B4	Human coxsackievirus B4	MG45180 8	6815	01/10/2 017	Human	B type
978	EPV3715	Human coxsackievirus B4	KY36990 4	7368	2016	Human	B type
979	HN23/CHN/2013	Human coxsackievirus B4	KU56650 7	7394	2013	Human	B type
980	Laiwu/SD/CHN/2013	Human coxsackievirus B4	KX75278 4	7372	06/04/2 013	Human	B type
981	NSW-V11-2010-CVB4	Human coxsackievirus B4	MF67830 0	7013	2010	Human	B type
982	NSW-V31-2010-CVB4	Human coxsackievirus B4	MF67831 9	6878	2010	Human	B type

983	NSW-V57-2007-CVB4	Human coxsackievirus B4	MF678347	6885	2007	Human	B type
984	10T1	Human coxsackievirus B5	MN749162	7409	2013	Human	B type
985	10T2	Human coxsackievirus B5	MN749163	7406	2013	Human	B type
986	12C8	Human coxsackievirus B5	MN749140	7411	2015	Human	B type
987	NSW-V50-2008-CVB5	Human coxsackievirus B5	MF678340	6897	2008	Human	B type
988	NSW-V51-2008-CVB5	Human coxsackievirus B5	MF678341	6894	2008	Human	B type
989	USA/MI/2009-23030	Human coxsackievirus B5	MH752988	7307	2009	Human	B type
990	AKS-AWT-AFP2F/XJ/CHN/2011	Human enterovirus 106	KX171334	7422	05/24/2011	Human	B type
991	HTPS-QDH11F/XJ/CHN/2011	Human enterovirus 106	KX171335	7421	09/06/2011	Human	B type
992	KS-MGTH90F/XJ/CHN/2011	Human enterovirus 106	KX171337	7421	09/09/2011	Human	B type
993	17-2255-1_E79	Human enterovirus 79	MH144598	7375	2017	Human	B type
994	USA/CA79-10384	Human enterovirus 79	AY843297	7430	-N/A-	Human	B type
995	99052/XZ/CHN/1999	Human enterovirus 93	MN580134	7432	08/11/1999	Human	B type
996	99096/XZ/CHN/1999	Human enterovirus 93	MN58013	7395	08/12/1	Human	B type

			5		999		
997	99167/XZ/CHN/1999	Human enterovirus 93	MN580136	7438	09/04/1999	Human	B type
998	1222/YN/CHN/2010	Coxsackievirus A1	MK250423	7396	12/09/2010	Human	C type
999	HT-THLH02F/XJ/CHN/2011	Coxsackievirus A1	JX174176	7397	09/02/2011	Human	C type
1000	KS-ZPH01F/XJ/CHN/2011	Coxsackievirus A1	JX174177	7398	09/06/2011	Human	C type
1001	Tompkins	Coxsackievirus A1	AF499635	7397	-N/A-	Human	C type
1002	BAN00-10444	Coxsackievirus A11	DQ995633	7452	-N/A-	Human	C type
1003	BAN01-10589	Coxsackievirus A11	DQ995634	7454	-N/A-	Human	C type
1004	Belgium-1	Coxsackievirus A11	AF499636	7453	-N/A-	Human	C type
1005	CV-A11_66122	Coxsackievirus A11	JF260917	7478	-N/A-	Human	C type
1006	CV-A11_66990	Coxsackievirus A11	JF260918	7474	-N/A-	Human	C type
1007	CV-A11_67874	Coxsackievirus A11	JF260919	7475	-N/A-	Human	C type
1008	Human-HW349523	Coxsackievirus A11	HW349523	7453	-N/A-	Human	C type
1009	ARG98-10613	Coxsackievirus A13	DQ995635	7461	-N/A-	Human	C type
1010	ARG98-10614	Coxsackievirus A13	DQ995636	7462	-N/A-	Human	C type
1011	AUS89-10611	Coxsackievirus A13	DQ99563	7461	-N/A-	Human	C type

			7				
1012	BAN00-10494	Coxsackievirus A13	DQ99563 9	7457	-N/A-	Human	C type
1013	BAN00-10562	Coxsackievirus A13	DQ99564 3	7458	-N/A-	Human	C type
1014	BAN00-10564	Coxsackievirus A13	DQ99564 2	7457	-N/A-	Human	C type
1015	BAN01-10637	Coxsackievirus A13	DQ99563 8	7459	-N/A-	Human	C type
1016	BAN99-10419	Coxsackievirus A13	DQ99564 0	7457	-N/A-	Human	C type
1017	BAN99-10430	Coxsackievirus A13	DQ99564 1	7458	-N/A-	Human	C type
1018	CV-A13_67001	Coxsackievirus A13	JF260920	7482	-N/A-	Human	C type
1019	CV-A13_67900	Coxsackievirus A13	JF260921	7479	-N/A-	Human	C type
1020	CV-A13_68095	Coxsackievirus A13	JF260922	7483	-N/A-	Human	C type
1021	CV-A13_68145	Coxsackievirus A13	JF260923	7482	-N/A-	Human	C type
1022	Flores	Coxsackievirus A13	AF465511	7458	-N/A-	Human	C type
1023	Flores	Coxsackievirus A13	AF499637	7458	-N/A-	Human	C type
1024	USA/Ca98-10615	Coxsackievirus A13	DQ99564 4	7458	-N/A-	Human	C type
1025	G-9	Coxsackievirus A15	AF465512	7441	-N/A-	Human	C type
1026	G9	Coxsackievirus A15	AF499638	7441	-N/A-	Human	C type
1027	BAN01-10577	Coxsackievirus A17	DQ99564	7454	-N/A-	Human	C type

			5				
1028	BAN03-10616	Coxsackievirus A17	DQ99564<br>6	7456	-N/A-	Human	C type
1029	cCA17.67591	Coxsackievirus A17	FM95527<br>8	7457	2002	Human	C type
1030	CV-A17_67610	Coxsackievirus A17	JF260924	7465	-N/A-	Human	C type
1031	CV-A17_68154	Coxsackievirus A17	JF260925	7477	-N/A-	Human	C type
1032	G12	Coxsackievirus A17	AF499639	7457	-N/A-	Human	C type
1033	CAM1972	Coxsackievirus A18	AB20539<br>6	7460	-N/A-	Human	C type
1034	G-13	Coxsackievirus A18	AF465513	7457	-N/A-	Human	C type
1035	G13	Coxsackievirus A18	AF499640	7458	-N/A-	Human	C type
1036	2019103106/XX/CHN/2019	Coxsackievirus A19	MT17570<br>6	7409	10/2019	Human	C type
1037	8663	Coxsackievirus A19	AF499641	7410	-N/A-	Human	C type
1038	NSW-V03-2008-CVA19	Coxsackievirus A19	MF67829<br>4	6950	2008	Human	C type
1039	89490	Coxsackievirus A20	DQ35807<br>8	7444	1988	Human	C type
1040	BAN00-10447	Coxsackievirus A20	EF015017	7440	-N/A-	Human	C type
1041	BAN00-10462	Coxsackievirus A20	EF015013	7441	-N/A-	Human	C type
1042	BAN00-10529	Coxsackievirus A20	EF015015	7441	-N/A-	Human	C type
1043	BAN00-10538	Coxsackievirus A20	EF015018	7441	-N/A-	Human	C type

1044	BAN01-10618	Coxsackievirus A20	EF015014	7441	-N/A-	Human	C type
1045	BAN99-10635	Coxsackievirus A20	EF015016	7441	-N/A-	Human	C type
1046	CVA20a-Tulane	Coxsackievirus A20	EF015021	7438	-N/A-	Human	C type
1047	CVA20b-Cecil	Coxsackievirus A20	EF015019	7439	-N/A-	Human	C type
1048	IH Pool 35	Coxsackievirus A20	AF465514	7436	-N/A-	Human	C type
1049	IH35	Coxsackievirus A20	AF499642	7436	-N/A-	Human	C type
1050	MOR83-10617	Coxsackievirus A20	EF015020	7441	-N/A-	Human	C type
1051	BAN00-10467	Coxsackievirus A21	EF015031	7440	-N/A-	Human	C type
1052	BAN00-10542	Coxsackievirus A21	EF015029	7441	-N/A-	Human	C type
1053	BAN00-10552	Coxsackievirus A21	EF015030	7441	-N/A-	Human	C type
1054	BAN00-10566	Coxsackievirus A21	EF015032	7439	-N/A-	Human	C type
1055	GUT88-10619	Coxsackievirus A21	EF015027	7438	-N/A-	Human	C type
1056	GUT88-10687	Coxsackievirus A21	EF015028	7438	-N/A-	Human	C type
1057	JN12377/SD/CHN/2012/CVA21	Coxsackievirus A21	KT161266	7405	2012	Human	C type
1058	Kuykendall	Coxsackievirus A21	AF465515	7405	-N/A-	Human	C type
1059	Kuykendall	Coxsackievirus A21	AF546702	7406	-N/A-	Human	C type
1060	438913	Coxsackievirus A22	JN542510	7404	07/2010	Human	C type
1061	ban99-10427	Coxsackievirus A22	DQ99564 7	7401	-N/A-	Human	C type
1062	Chulman	Coxsackievirus A22	AF499643	7406	-N/A-	Human	C type
1063	USA75-10624	Coxsackievirus A22	DQ99564 8	7402	-N/A-	Human	C type

1064	110390	Coxsackievirus A24	KF725085	7458	07/01/2002	Human	C type
1065	BRA87-10629	Coxsackievirus A24	EF015038	7456	-N/A-	Human	C type
1066	CA24v/Ishigaki/28/2011	Coxsackievirus A24	AB769163	7462	10/2011	Human	C type
1067	CA24v/Ishigaki/29/2011	Coxsackievirus A24	AB769164	7462	10/2011	Human	C type
1068	CA24v/Ishigaki/35/2011	Coxsackievirus A24	AB769165	7462	10/2011	Human	C type
1069	CA24v/Okinawa/19/2011	Coxsackievirus A24	AB769159	7462	06/2011	Human	C type
1070	CA24v/Okinawa/20/2011	Coxsackievirus A24	AB769160	7462	06/2011	Human	C type
1071	CA24v/Okinawa/23/2011	Coxsackievirus A24	AB769161	7462	06/2011	Human	C type
1072	CA24v/Okinawa/25/2011	Coxsackievirus A24	AB769162	7462	06/2011	Human	C type
1073	CA24v/Okinawa/5/2011	Coxsackievirus A24	AB769152	7462	06/2011	Human	C type
1074	CA24v/Okinawa/7/2011	Coxsackievirus A24	AB769154	7462	06/2011	Human	C type
1075	CA24v/Okinawa/9/2011	Coxsackievirus A24	AB769156	7462	06/2011	Human	C type
1076	China/GD01/2010	Coxsackievirus A24	JF742577	7456	09/14/2010	Human	C type
1077	China/GD332/2007	Coxsackievirus A24	JF742579	7456	2007	Human	C type

1078	China/GD391/2007	Coxsackievirus A24	JF742578	7456	2007	Human	C type
1079	China/GD46/2010	Coxsackievirus A24	JF742576	7457	09/30/2010	Human	C type
1080	DOR93-10630	Coxsackievirus A24	EF015039	7459	-N/A-	Human	C type
1081	DSO-26/2005	Coxsackievirus A24	DQ443002	7461	-N/A-	Human	C type
1082	DSO-52/2005	Coxsackievirus A24	DQ443001	7461	-N/A-	Human	C type
1083	E34-dn19	Coxsackievirus A24	EF015034	7458	-N/A-	Human	C type
1084	EH24/70	Coxsackievirus A24	D90457	7461	-N/A-	Human	C type
1085	HG	Coxsackievirus A24	JN228097	7460	08/08/2004	Human	C type
1086	INDNIV1034661LV463	Coxsackievirus A24	KF667358	7461	2010	Human	C type
1087	INDNIV1036731LV476	Coxsackievirus A24	KF667359	7460	2010	Human	C type
1088	INDNIV1040633LV639	Coxsackievirus A24	KF667361	7459	2010	Human	C type
1089	INDNIV1044161LV530	Coxsackievirus A24	KF667360	7459	2010	Human	C type
1090	JAM87-10628	Coxsackievirus A24	EF015037	7455	-N/A-	Human	C type
1091	Joseph	Coxsackievirus A24	EF026081	7459	1952	Human	C type
1092	PUR82-10626	Coxsackievirus A24	EF015036	7461	-N/A-	Human	C type
1093	PUR82-10686	Coxsackievirus A24	EF015035	7462	-N/A-	Human	C type
1094	USA-Fl98 10631	Coxsackievirus A24	EF015040	7459	-N/A-	Human	C type
1095	USA-Tx79-10625	Coxsackievirus A24	EF015033	7459	-N/A-	Human	C type
1096	03-0100	Enterovirus C	MN91420	6875	2003	Human	C type

			6				
1097	03-4101	Enterovirus C	MN914196	7256	2003	Human	C type
1098	03-4107	Enterovirus C	MN914200	6810	2003	Human	C type
1099	03-4166	Enterovirus C	MN914199	6856	2003	Human	C type
1100	03-4209	Enterovirus C	MN914197	7286	2003	Human	C type
1101	04-1378	Enterovirus C	MN914198	6849	2004	Human	C type
1102	04-1438	Enterovirus C	MN914203	7312	2004	Human	C type
1103	04-1450	Enterovirus C	MN914201	7333	2004	Human	C type
1104	04-4444	Enterovirus C	MN914204	6845	2004	Human	C type
1105	04-4491	Enterovirus C	MN914205	7383	2004	Human	C type
1106	04-4517	Enterovirus C	MN914202	6998	2004	Human	C type
1107	Brunenders	Enterovirus C	KP793687	7507	-N/A-	Human	C type
1108	Cambodia-02	Enterovirus C	AB205395	7431	-N/A-	Human	C type
1109	EQG1419332	Enterovirus C	KX16269	7422	03/31/2	Human	C type

			4		014		
1110	GUF-2017-B0519079	Enterovirus C	MG55756 1	7415	2017	Human	C type
1111	GUF-2017-B0607050	Enterovirus C	MG55756 3	7415	2017	Human	C type
1112	GUF-2017-B0627045	Enterovirus C	MG55756 2	7415	2017	Human	C type
1113	Human poliovirus 1 Mahoney	Enterovirus C	NC_0020 58 *	7440	-N/A-	Human	C type
1114	InDRE2764/MEX/2017	Enterovirus C	MG88074 5	7436	10/11/20 17	Human	C type
1115	InDRE2768/MEX/2017	Enterovirus C	MG88074 7	7436	10/02/2 017	Human	C type
1116	InDRE2771/MEX/2017	Enterovirus C	MG88074 8	7435	10/02/2 017	Human	C type
1117	InDRE2773/MEX/2017	Enterovirus C	MG88074 9	7436	10/11/20 17	Human	C type
1118	InDRE2778/MEX/2017	Enterovirus C	MG88075 0	7436	10/11/20 17	Human	C type
1119	InDRE2780/MEX/2017	Enterovirus C	MG88075 1	7436	10/02/2 017	Human	C type
1120	InDRE2782/MEX/2017	Enterovirus C	MG88075 2	7436	10/02/2 017	Human	C type
1121	Polozj-3	Enterovirus C	MZ54618 8	7459	2016	Human	C type
1122	PUFFIN095-2	Enterovirus C	MT95717	7465	2014	Human	C type

			9				
1123	PUFFIN104-3	Enterovirus C	MT95718 1	7421	2014	Human	C type
1124	PUFFIN110-1	Enterovirus C	MT95718 2	7469	2014	Human	C type
1125	PUFFIN110-3	Enterovirus C	MT95718 3	7460	2014	Human	C type
1126	PUFFIN122-1	Enterovirus C	MT95718 8	7423	2014	Human	C type
1127	PUFFIN122-2	Enterovirus C	MT95718 9	7454	2014	Human	C type
1128	PUFFIN131-3	Enterovirus C	MT95719 0	7469	2014	Human	C type
1129	PUFFIN132-3	Enterovirus C	MT95719 1	7494	2014	Human	C type
1130	PUFFIN133-2	Enterovirus C	MT95719 2	7547	2014	Human	C type
1131	PUFFIN134-3	Enterovirus C	MT95719 3	7648	2014	Human	C type
1132	PUFFIN137-1	Enterovirus C	MT95719 4	7694	2014	Human	C type
1133	PUFFIN137-2	Enterovirus C	MT95719 5	7683	2014	Human	C type
1134	PUFFIN137-3	Enterovirus C	MT95719 6	7548	2014	Human	C type
1135	PUFFIN138-2	Enterovirus C	MT95719	7441	2014	Human	C type

			7				
1136	PUFFIN145-3	Enterovirus C	MT957198	7458	2014	Human	C type
1137	PUFFIN146-2	Enterovirus C	MT957199	7502	2014	Human	C type
1138	PUFFIN160-2	Enterovirus C	MT957201	7453	2014	Human	C type
1139	PUFFIN161-1	Enterovirus C	MT957202	7457	2014	Human	C type
1140	PUFFIN161-3	Enterovirus C	MT957203	7420	2014	Human	C type
1141	PUFFIN164-1	Enterovirus C	MT957204	7557	2014	Human	C type
1142	PUFFIN176-3	Enterovirus C	MT957207	7445	2014	Human	C type
1143	V2-BEL	Enterovirus C	HQ738286	7445	2005	Human	C type
1144	V2-Sak	Enterovirus C	HQ738287	7445	2005	Human	C type
1145	V2-TOL cl1	Enterovirus C	HQ738288	7445	2005	Human	C type
1146	V2-TOL cl2	Enterovirus C	HQ738289	7445	2005	Human	C type
1147	V2-Tol.1	Enterovirus C	HQ738303	7407	2005	Human	C type
1148	V2-TSI	Enterovirus C	HQ73829	7444	2005	Human	C type

			0				
1149	V2-Tsi.1	Enterovirus C	HQ73829<br>2	7409	2005	Human	C type
1150	V2-Tsi.2	Enterovirus C	HQ73829<br>3	7409	2005	Human	C type
1151	V2-Tsi.3	Enterovirus C	HQ73829<br>4	7409	2005	Human	C type
1152	V2-Tsi.4	Enterovirus C	HQ73829<br>5	7409	2005	Human	C type
1153	V3-TUL	Enterovirus C	HQ73829<br>1	7432	2005	Human	C type
1154	V3-Tul.1	Enterovirus C	HQ73829<br>6	7397	2005	Human	C type
1155	V3-Tul.2	Enterovirus C	HQ73829<br>7	7397	2005	Human	C type
1156	V3-Tul.3	Enterovirus C	HQ73829<br>8	7397	2005	Human	C type
1157	V3-Tul.4	Enterovirus C	HQ73829<br>9	7397	2005	Human	C type
1158	V3-Tul.5	Enterovirus C	HQ73830<br>0	7397	2005	Human	C type
1159	V3-Tul.6	Enterovirus C	HQ73830<br>1	7397	2005	Human	C type
1160	V3-Tul.7	Enterovirus C	HQ73830<br>2	7397	2005	Human	C type
1161	WIV14	Enterovirus C	KY70369	7432	12/2014	Human	C type

			7				
1162	AK11	Enterovirus C104	AB68652 4	7408	02/2011	Human	C type
1163	NL_2018_R1	Enterovirus C104	MZ09270 2	7390	11/27/20 18	Human	C type
1164	NL_2019_R3	Enterovirus C104	MZ09270 4	7411	01/28/2 019	Human	C type
1165	JX-C117-40-2017	enterovirus C117	MT33856 7	7363	05/04/2 017	Human	C type
1166	5517	Enterovirus C96	HQ41575 8	7470	2005	Human	C type
1167	09228C1	Enterovirus C96	HQ41575 9	7469	2009	Human	C type
1168	AFP809/GD/CHN/2011	Enterovirus C96	KF495604	7469	11/22/20 11	Human	C type
1169	BAN00-10488	Enterovirus C96	EF015886	7478	-N/A-	Human	C type
1170	FIN04-7	Enterovirus C96	FJ751914	7475	-N/A-	Human	C type
1171	FIN05-2	Enterovirus C96	FJ751915	7471	-N/A-	Human	C type
1172	SZ/GD/CHN/2015	Enterovirus C96	KR91980 4	7451	2015	Human	C type
1173	T49-XZ-CHN-2005	Enterovirus C96	KP98475 3	7471	2005	Human	C type
1174	XZ94-XZ-CHN-2012	Enterovirus C96	KP98475 4	7453	2012	Human	C type
1175	10L1	Enterovirus C99	MN91861 3	7416	2013	Human	C type

1176	12-008-2_C99	Enterovirus C99	MH144606	7423	2012	Human	C type
1177	3291/BRA-PA/10	Enterovirus C99	MH484164	7470	07/2010	Human	C type
1178	3944/BRA-PA/11	Enterovirus C99	MH484166	7453	02/2011	Human	C type
1179	BAN00-10461	Enterovirus C99	EF015008	7451	-N/A-	Human	C type
1180	BAN01-10582	Enterovirus C99	EF015009	7448	-N/A-	Human	C type
1181	BAN04-10697	Enterovirus C99	EF015010	7448	-N/A-	Human	C type
1182	HEV-99_68229	Enterovirus C99	JF260926	7467	-N/A-	Human	C type
1183	HT-XEBGH09F/XJ/CHN/2011	Enterovirus C99	KF129411	7455	08/30/2011	Human	C type
1184	Human/EV-C99/BRA/TO-16/BRA/2013	Enterovirus C99	MK689071	7415	2013	Human	C type
1185	K292/YN/CHN/2013	Enterovirus C99	KT946713	7453	05/05/2013	Human	C type
1186	KSSC-ALXHH01F/XJ/CHN/2011	Enterovirus C99	KF129412	7455	09/08/2011	Human	C type
1187	OMA99-10696	Enterovirus C99	EF015011	7455	-N/A-	Human	C type
1188	USA-GA84-10636	Enterovirus C99	EF555644	7447	-N/A-	Human	C type
1189	USA-Ok85-10627	Enterovirus C99	EF015012	7447	-N/A-	Human	C type
1190	YT23/SD/CHN/11	Enterovirus C99	KJ857507	7454	2011	Human	C type
1191	YT31/SD/CHN/11	Enterovirus C99	KJ857508	7456	2011	Human	C type
1192	Human-LP131905	Human coxsackievirus A11	LP131905	7453	-N/A-	Human	C type

1193	Human-MA783942	Human coxsackievirus A11	MA78394 2	7453	-N/A-	Human	C type
1194	Human-MP510547	Human coxsackievirus A11	MP51054 7	7453	-N/A-	Human	C type
1195	CAM1976	Human coxsackievirus A20	LC279542	7443	-N/A-	Human	C type
1196	NGR_2016	Human coxsackievirus A20	MH78518 3	7091	2016	Human	C type
1197	12MYKLU412	Human coxsackievirus A21	MW36696 3	7339	05/02/2 012	Human	C type
1198	12MYKLU434	Human coxsackievirus A21	MW36696 4	7339	05/04/2 012	Human	C type
1199	14MYKLU3370	Human coxsackievirus A21	MW36696 5	7339	01/13/2 014	Human	C type
1200	429/Ft Jackson South Carolina USA/1998	Human coxsackievirus A21	KX38496 2	7346	1998	Human	C type
1201	NIV17-608-2	Human coxsackievirus A21	MG98266 3	7422	2017	Human	C type
1202	T197/Ft Jackson South Carolina USA/2002	Human coxsackievirus A21	KX38496 1	7388	11/29/20 02	Human	C type
1203	USA/TN/2015-OB2038	Human coxsackievirus A21	KY27194 7	7317	05/31/2 015	Human	C type
1204	V12125(MSH6283)/Ft Jackson South Carolina USA/2000	Human coxsackievirus A21	KX38496 3	7334	2000	Human	C type
1205	V16073(UC8)/Ft Jackson South Carolina USA/2004	Human coxsackievirus A21	KX38496 4	7339	2004	Human	C type
1206	Coe	Human coxsackievirus A21	D00538	7401	-N/A-	Human	C type

		Coe						
1207	20693_66_CV-A24	Human coxsackievirus A24	MK989719	7422	06/03/2010	Human	C type	
1208	20693_84_CV-A24	Human coxsackievirus A24	MK989721	7477	05/26/2010	Human	C type	
1209	GUF-2017-B0519081	Human coxsackievirus A24	MF419263	7415	06/2017	Human	C type	
1210	K282/YN/CHN/2013	Human coxsackievirus A24	KU183495	7461	04/28/2013	Human	C type	
1211	Reunion0150638129-FRA15	Human coxsackievirus A24	KR399988	7458	03/12/2015	Human	C type	
1212	Reunion0150638154-FRA15	Human coxsackievirus A24	KR478685	7439	03/12/2015	Human	C type	
1213	BAN99-10424	Human enterovirus C102	EF555645	7438	-N/A-	Human	C type	
1214	C104	Human enterovirus C104	KR815824	7408	02/17/2014	Human	C type	
1215	Pavia259-7712	Human enterovirus C104	JX982253	7409	04/2009	Human	C type	
1216	Pavia260-9210	Human enterovirus C104	JX982254	7409	04/2009	Human	C type	
1217	Pavia261-9570	Human enterovirus C104	JX982257	7409	04/2009	Human	C type	
1218	Pavia262-11228	Human enterovirus C104	JX982259	7409	05/2009	Human	C type	
1219	Pavia263-11230	Human enterovirus C104	JX982255	7409	05/2009	Human	C type	
1220	Pavia264-16291	Human enterovirus C104	JX982256	7409	05/2009	Human	C type	
1221	Pavia68-10804B	Human enterovirus C104	JX982258	7409	05/2009	Human	C type	
1222	R2759	Human enterovirus C105	MH22999	7316	05/10/2	Human	C type	

			7		016		
1223	NICA08-4327	Human enterovirus C109	GQ865517	7354	02/23/2008	Human	C type
1224	USA/FL/2016-21002	Human enterovirus C109	MH128992	7336	06/11/2016	Human	C type
1225	USA/FL/2016-21003	Human enterovirus C109	MH128993	7327	02/17/2016	Human	C type
1226	CQ6747	Human enterovirus C117	MK089787	7364	06/11/2014	Human	C type
1227	LIT22	Human enterovirus C117	JX262382	7363	06/2011	Human	C type
1228	CQ5185	Human enterovirus C118	JX678288	7374	06/14/2011	Human	C type
1229	ISR10	Human enterovirus C118	JX961708	7357	01/2011	Human	C type
1230	ISR38	Human enterovirus C118	JX961709	7357	03/2011	Human	C type
1231	127/SD/CHN/1991	Human enterovirus C96	MK512666	7477	1991	Human	C type
1232	184C5/SD/CHN/2009	Human enterovirus C96	MK512667	7468	2009	Human	C type
1233	3499/BRA-PA/10	Human enterovirus C96	MH484165	7713	09/2010	Human	C type
1234	JN13048	Human enterovirus C96	MK512668	7470	2013	Human	C type
1235	Hangzhou13-02	Human enterovirus Hangzhou13-02	AY876913	7456	-N/A-	Human	C type
1236	Ningbo3-02	Human enterovirus Ningbo3-02	AY876912	7456	-N/A-	Human	C type

1237	1-B2	Human poliovirus 1	KT353719	7423	2008	Human	C type
1238	10050	Human poliovirus 1	FJ859058	7441	03/18/2006	Human	C type
1239	10086c	Human poliovirus 1	FJ859059	7441	05/15/2006	Human	C type
1240	10091c	Human poliovirus 1	FJ859060	7441	05/16/2006	Human	C type
1241	10092c	Human poliovirus 1	FJ859061	7441	05/16/2006	Human	C type
1242	10094c	Human poliovirus 1	FJ859062	7441	05/16/2006	Human	C type
1243	10095c	Human poliovirus 1	FJ859063	7441	05/16/2006	Human	C type
1244	10097c	Human poliovirus 1	FJ859064	7441	05/16/2006	Human	C type
1245	18339	Human poliovirus 1	MG212479	7460	2002	Human	C type
1246	1PMR046018	Human poliovirus 1	MG212429	7478	-N/A-	Human	C type
1247	28698	Human poliovirus 1	MG212433	7438	1993	Human	C type
1248	29690_c1	Human poliovirus 1	MG212437	7437	-N/A-	Human	C type
1249	29690_c6	Human poliovirus 1	MG212436	7437	-N/A-	Human	C type
1250	29690_c9	Human poliovirus 1	MG21243	7437	-N/A-	Human	C type

			8				
1251	36712	Human poliovirus 1	KC880382	7442	06/02/2010	Human	C type
1252	558/BRA-PE/88	Human poliovirus 1	KF537633	7445	1988	Human	C type
1253	8757	Human poliovirus 1	MG212472	7452	1998	Human	C type
1254	99/056-252-14	Human poliovirus 1	AF462418	7441	-N/A-	Human	C type
1255	Brunhilde	Human poliovirus 1	AY560657	7445	-N/A-	Human	C type
1256	CAE1419303	Human poliovirus 1	KX162679	7363	01/23/2014	Human	C type
1257	CAE1419304	Human poliovirus 1	KX162680	7431	02/02/2014	Human	C type
1258	CAE1419305	Human poliovirus 1	KX162681	7430	02/16/2014	Human	C type
1259	CAF2019340	Human poliovirus 1	KX162682	7360	10/11/2011	Human	C type
1260	CHA1119341	Human poliovirus 1	KX162683	7362	11/25/2011	Human	C type
1261	CHA1218878	Human poliovirus 1	KX162684	7435	06/23/2012	Human	C type
1262	CHAT 10A-11	Human poliovirus 1	AJ416942	7440	-N/A-	Human	C type
1263	CHN-Fujian/93-8; CHN-6343FJ93	Human poliovirus 1	AF111981	7444	-N/A-	Human	C type
1264	CHN-Guangdong/92-2; CHN-5157GD92	Human poliovirus 1	AF111961	7444	-N/A-	Human	C type
1265	CHN-Hainan/93-2; CHN-5732HA93	Human poliovirus 1	AF111966	7444	-N/A-	Human	C type

1266	CHN-Hebei/91-2; CHN-3645HB91	Human poliovirus 1	AF111953	7445	-N/A-	Human	C type
1267	CHN-Henan/91-3; CHN-3653HE91	Human poliovirus 1	AF111983	7444	-N/A-	Human	C type
1268	CHN-Jiangxi/89-1; CHN-1338JX89	Human poliovirus 1	AF111984	7443	-N/A-	Human	C type
1269	CHN-Yunnan/92; CHN-6421YN92	Human poliovirus 1	AF111982	7444	-N/A-	Human	C type
1270	CHN15115/Xinjiang/CHN/2011	Human poliovirus 1	MH75091<br>2	7442	2011	Human	C type
1271	CHN8184/GZ/CHN/2004	Human poliovirus 1	FJ769378	7441	07/13/2<br>004	Human	C type
1272	CHN8225c/GZ/CHN/2004	Human poliovirus 1	FJ769383	7441	08/08/2<br>004	Human	C type
1273	CHN8229-1/GZ/CHN/2004	Human poliovirus 1	FJ769379	7441	07/26/2<br>004	Human	C type
1274	CHN8229-2/GZ/CHN/2004	Human poliovirus 1	FJ769380	7441	08/10/2<br>004	Human	C type
1275	CHN8229-3/GZ/CHN/2004	Human poliovirus 1	FJ769381	7441	08/16/2<br>004	Human	C type
1276	CHN8233c/GZ/CHN/2004	Human poliovirus 1	FJ769382	7441	06/15/2<br>004	Human	C type
1277	CHN8248c/GZ/CHN/2004	Human poliovirus 1	FJ769384	7441	08/06/2<br>004	Human	C type
1278	CHN8264c/GZ/CHN/2004	Human poliovirus 1	FJ769385	7441	08/07/2<br>004	Human	C type
1279	Cox	Human poliovirus 1	AJ430385	7441	-N/A-	Human	C type
1280	d018-2002037602	Human poliovirus 1	AY928387	7432	-N/A-	Human	C type
1281	d054-2002037604	Human poliovirus 1	AY928385	7432	-N/A-	Human	C type

1282	d179-2002037605	Human poliovirus 1	AY928386	7432	-N/A-	Human	C type
1283	d224-2002037606	Human poliovirus 1	AY928384	7432	-N/A-	Human	C type
1284	d261-2002037607	Human poliovirus 1	AY928383	7433	-N/A-	Human	C type
1285	DOR00013	Human poliovirus 1	AF405690	7441	-N/A-	Human	C type
1286	DOR00015	Human poliovirus 1	AF405688	7441	-N/A-	Human	C type
1287	DOR00016	Human poliovirus 1	AF405689	7441	-N/A-	Human	C type
1288	DOR00023C	Human poliovirus 1	AF405685	7441	-N/A-	Human	C type
1289	DOR00024	Human poliovirus 1	AF405687	7441	-N/A-	Human	C type
1290	DOR00025	Human poliovirus 1	AF405686	7441	-N/A-	Human	C type
1291	DOR00028	Human poliovirus 1	AF405684	7441	-N/A-	Human	C type
1292	DOR00028C	Human poliovirus 1	AF405683	7441	-N/A-	Human	C type
1293	DOR00041C1	Human poliovirus 1	AF405682	7441	-N/A-	Human	C type
1294	DOR00041C2	Human poliovirus 1	AF405680	7441	-N/A-	Human	C type
1295	DOR00041C3	Human poliovirus 1	AF405679	7441	-N/A-	Human	C type
1296	DOR00042	Human poliovirus 1	AF405678	7441	-N/A-	Human	C type
1297	DOR00042C1	Human poliovirus 1	AF405677	7441	-N/A-	Human	C type
1298	DOR00042C2	Human poliovirus 1	AF405676	7441	-N/A-	Human	C type
1299	DOR00044	Human poliovirus 1	AF405681	7441	-N/A-	Human	C type
1300	DOR01001	Human poliovirus 1	AF405675	7441	-N/A-	Human	C type
1301	DOR01001C1	Human poliovirus 1	AF405674	7441	-N/A-	Human	C type
1302	DOR01001C2	Human poliovirus 1	AF405673	7441	-N/A-	Human	C type

1303	DOR01002	Human poliovirus 1	AF405672	7441	-N/A-	Human	C type
1304	DOR01002C	Human poliovirus 1	AF405671	7441	-N/A-	Human	C type
1305	DOR01012	Human poliovirus 1	AF405670	7441	-N/A-	Human	C type
1306	EGY1218585	Human poliovirus 1	KJ155499	7443	2012	Human	C type
1307	EGY1218586	Human poliovirus 1	KJ155500	7443	2012	Human	C type
1308	EGY1218587	Human poliovirus 1	KJ155495	7443	2012	Human	C type
1309	EGY1218588	Human poliovirus 1	KJ155496	7443	2012	Human	C type
1310	EQG1418881	Human poliovirus 1	KX162691	7424	06/02/2014	Human	C type
1311	EQG1419328	Human poliovirus 1	KX162692	7428	02/09/2014	Human	C type
1312	EQG1419331	Human poliovirus 1	KX162693	7418	03/24/2014	Human	C type
1313	EQG1419333	Human poliovirus 1	KX162695	7301	04/29/2014	Human	C type
1314	ETH1319533	Human poliovirus 1	KY941931	7450	2013	Human	C type
1315	HAI00003	Human poliovirus 1	AF405669	7442	-N/A-	Human	C type
1316	HAI01001	Human poliovirus 1	AF405668	7441	-N/A-	Human	C type
1317	HAI01002	Human poliovirus 1	AF405667	7441	-N/A-	Human	C type
1318	HAI01007	Human poliovirus 1	AF405666	7441	-N/A-	Human	C type
1319	HAI01008	Human poliovirus 1	AF405665	7441	-N/A-	Human	C type
1320	HAI01008C1	Human poliovirus 1	AF405663	7441	-N/A-	Human	C type

1321	HAI01008C2	Human poliovirus 1	AF405662	7441	-N/A-	Human	C type
1322	HAI01009	Human poliovirus 1	AF405664	7441	-N/A-	Human	C type
1323	HAI01013all	Human poliovirus 1	AF416342	7441	-N/A-	Human	C type
1324	HAI01015	Human poliovirus 1	AF458333	7441	-N/A-	Human	C type
1325	ISR1318572	Human poliovirus 1	KJ019831	7443	2013	Human	C type
1326	ISR1318573	Human poliovirus 1	KJ019832	7443	2013	Human	C type
1327	ISR1318574	Human poliovirus 1	KJ019833	7443	2013	Human	C type
1328	KAZ38374	Human poliovirus 1	KC880371	7442	08/12/2010	Human	C type
1329	Luzon-01-1	Human poliovirus 1	AB180071	7440	-N/A-	Human	C type
1330	Luzon-01-2	Human poliovirus 1	AB180072	7440	-N/A-	Human	C type
1331	Luzon-01-2c	Human poliovirus 1	AB180073	7440	-N/A-	Human	C type
1332	Mahoney	Human poliovirus 1	V01148	7433	-N/A-	Human	C type
1333	Mahoney_CDC	Human poliovirus 1	KU866422	7441	-N/A-	Human	C type
1334	Mindanao-01-1	Human poliovirus 1	AB180070	7440	-N/A-	Human	C type
1335	NIE0918311	Human poliovirus 1	KJ170467	7388	2009	Human	C type
1336	NIE0918312	Human poliovirus 1	KJ170484	7388	2009	Human	C type
1337	NIE0918313	Human poliovirus 1	KJ170528	7388	2009	Human	C type

1338	NIE0918314	Human poliovirus 1	KJ170477	7388	2009	Human	C type
1339	NIE0918315	Human poliovirus 1	KJ170500	7388	2009	Human	C type
1340	NIE0918380	Human poliovirus 1	KJ170486	7388	2009	Human	C type
1341	NIE0918387	Human poliovirus 1	KJ170523	7388	2009	Human	C type
1342	NIE0918388	Human poliovirus 1	KJ170502	7388	2009	Human	C type
1343	NIE0918389	Human poliovirus 1	KJ170503	7388	2009	Human	C type
1344	NIE0918390	Human poliovirus 1	KJ170524	7388	2009	Human	C type
1345	NIE0918391	Human poliovirus 1	KJ170478	7388	2009	Human	C type
1346	NIE0918392	Human poliovirus 1	KJ170509	7388	2009	Human	C type
1347	NIE1018316	Human poliovirus 1	KJ170436	7388	2010	Human	C type
1348	NIE1018319	Human poliovirus 1	KJ170438	7388	2010	Human	C type
1349	NIE1018320	Human poliovirus 1	KJ170475	7388	2010	Human	C type
1350	NIE1018321	Human poliovirus 1	KJ170451	7388	2010	Human	C type
1351	NIE1018322	Human poliovirus 1	KJ170458	7388	2010	Human	C type
1352	NIE1018323	Human poliovirus 1	KJ170517	7388	2010	Human	C type
1353	NIE1018324	Human poliovirus 1	KJ170491	7388	2010	Human	C type
1354	NIE1018325	Human poliovirus 1	KJ170488	7388	2010	Human	C type
1355	NIE1018326	Human poliovirus 1	KJ170504	7388	2010	Human	C type
1356	NIE1018354	Human poliovirus 1	KJ170439	7388	2010	Human	C type
1357	NIE1018355	Human poliovirus 1	KJ170530	7388	2010	Human	C type
1358	NIE1018356	Human poliovirus 1	KJ170529	7388	2010	Human	C type

1359	NIE1018357	Human poliovirus 1	KJ170520	7388	2010	Human	C type
1360	NIE1018358	Human poliovirus 1	KJ170516	7388	2010	Human	C type
1361	NIE1018359	Human poliovirus 1	KJ170507	7388	2010	Human	C type
1362	NIE1018360	Human poliovirus 1	KJ170440	7388	2010	Human	C type
1363	NIE1018361	Human poliovirus 1	KJ170444	7388	2010	Human	C type
1364	NIE1018362	Human poliovirus 1	KJ170441	7388	2010	Human	C type
1365	NIE1018363	Human poliovirus 1	KJ170531	7388	2010	Human	C type
1366	NIE1018364	Human poliovirus 1	KJ170442	7388	2010	Human	C type
1367	NIE1018365	Human poliovirus 1	KJ170445	7388	2010	Human	C type
1368	NIE1018366	Human poliovirus 1	KJ170446	7388	2010	Human	C type
1369	NIE1018381	Human poliovirus 1	KJ170522	7388	2010	Human	C type
1370	NIE1018382	Human poliovirus 1	KJ170508	7388	2010	Human	C type
1371	NIE1018393	Human poliovirus 1	KJ170501	7388	2010	Human	C type
1372	NIE1018394	Human poliovirus 1	KJ170515	7388	2010	Human	C type
1373	NIE1018395	Human poliovirus 1	KJ170506	7388	2010	Human	C type
1374	NIE1018396	Human poliovirus 1	KJ170470	7388	2010	Human	C type
1375	NIE1116622	Human poliovirus 1	KX162708	7363	09/04/2011	Human	C type
1376	NIE1116623	Human poliovirus 1	KX162709	7076	09/18/2011	Human	C type
1377	NIE1118317	Human poliovirus 1	KJ170505	7388	2011	Human	C type
1378	NIE1118318	Human poliovirus 1	KJ170487	7388	2011	Human	C type

1379	NIE1118327	Human poliovirus 1	KJ170489	7388	2011	Human	C type
1380	NIE1118328	Human poliovirus 1	KJ170480	7388	2011	Human	C type
1381	NIE1118329	Human poliovirus 1	KJ170494	7388	2011	Human	C type
1382	NIE1118330	Human poliovirus 1	KJ170481	7388	2011	Human	C type
1383	NIE1118331	Human poliovirus 1	KJ170460	7388	2011	Human	C type
1384	NIE1118332	Human poliovirus 1	KJ170472	7388	2011	Human	C type
1385	NIE1118333	Human poliovirus 1	KJ170490	7388	2011	Human	C type
1386	NIE1118334	Human poliovirus 1	KJ170471	7388	2011	Human	C type
1387	NIE1118335	Human poliovirus 1	KJ170495	7388	2011	Human	C type
1388	NIE1118336	Human poliovirus 1	KJ170461	7388	2011	Human	C type
1389	NIE1118337	Human poliovirus 1	KJ170473	7388	2011	Human	C type
1390	NIE1118338	Human poliovirus 1	KJ170468	7388	2011	Human	C type
1391	NIE1118339	Human poliovirus 1	KJ170474	7388	2011	Human	C type
1392	NIE1118340	Human poliovirus 1	KJ170462	7388	2011	Human	C type
1393	NIE1118367	Human poliovirus 1	KJ170493	7388	2011	Human	C type
1394	NIE1118368	Human poliovirus 1	KJ170449	7388	2011	Human	C type
1395	NIE1118369	Human poliovirus 1	KJ170447	7388	2011	Human	C type
1396	NIE1118370	Human poliovirus 1	KJ170521	7388	2011	Human	C type
1397	NIE1118371	Human poliovirus 1	KJ170510	7388	2011	Human	C type
1398	NIE1118372	Human poliovirus 1	KJ170514	7388	2011	Human	C type
1399	NIE1118373	Human poliovirus 1	KJ170527	7388	2011	Human	C type

1400	NIE1118374	Human poliovirus 1	KJ170511	7388	2011	Human	C type
1401	NIE1118375	Human poliovirus 1	KJ170532	7388	2011	Human	C type
1402	NIE1118376	Human poliovirus 1	KJ170448	7388	2011	Human	C type
1403	NIE1118377	Human poliovirus 1	KJ170518	7388	2011	Human	C type
1404	NIE1118378	Human poliovirus 1	KJ170459	7388	2011	Human	C type
1405	NIE1118379	Human poliovirus 1	KJ170526	7388	2011	Human	C type
1406	NIE1118383	Human poliovirus 1	KJ170450	7388	2011	Human	C type
1407	NIE1118384	Human poliovirus 1	KJ170512	7388	2011	Human	C type
1408	NIE1118385	Human poliovirus 1	KJ170513	7388	2011	Human	C type
1409	NIE1118386	Human poliovirus 1	KJ170479	7388	2011	Human	C type
1410	NIE1118397	Human poliovirus 1	KJ170452	7388	2011	Human	C type
1411	NIE1118398	Human poliovirus 1	KJ170453	7388	2011	Human	C type
1412	NIE1118399	Human poliovirus 1	KJ170499	7388	2011	Human	C type
1413	NIE1118400	Human poliovirus 1	KJ170492	7388	2011	Human	C type
1414	NIE1118401	Human poliovirus 1	KJ170454	7388	2011	Human	C type
1415	NIE1118402	Human poliovirus 1	KJ170455	7388	2011	Human	C type
1416	NIE1118403	Human poliovirus 1	KJ170456	7388	2011	Human	C type
1417	NIE1118404	Human poliovirus 1	KJ170476	7388	2011	Human	C type
1418	NIE1118405	Human poliovirus 1	KJ170525	7388	2011	Human	C type
1419	NIE1118406	Human poliovirus 1	KJ170443	7388	2011	Human	C type
1420	NIE1118407	Human poliovirus 1	KJ170457	7388	2011	Human	C type

1421	NIE1218341	Human poliovirus 1	KJ170482	7388	2012	Human	C type
1422	NIE1218342	Human poliovirus 1	KJ170463	7388	2012	Human	C type
1423	NIE1218343	Human poliovirus 1	KJ170469	7388	2012	Human	C type
1424	NIE1218344	Human poliovirus 1	KJ170497	7388	2012	Human	C type
1425	NIE1218345	Human poliovirus 1	KJ170464	7388	2012	Human	C type
1426	Pak-1	Human poliovirus 1	KU161395	7441	06/09/2013	Human	C type
1427	Pak-11	Human poliovirus 1	KU161399	7445	06/09/2013	Human	C type
1428	Pak-12	Human poliovirus 1	KU161398	7433	06/09/2013	Human	C type
1429	Pak-2-1	Human poliovirus 1	KU161396	7441	06/09/2013	Human	C type
1430	Pak-2-2	Human poliovirus 1	KU161397	7441	06/09/2013	Human	C type
1431	PAK1519534	Human poliovirus 1	KY941935	7449	2015	Human	C type
1432	PV1-RC2010-45	Human poliovirus 1	JF838278	7443	2010	Human	C type
1433	R71712	Human poliovirus 1	KR259355	7436	2011	Human	C type
1434	RUS-1161-96-001	Human poliovirus 1	AF462419	7441	-N/A-	Human	C type
1435	RUS36716	Human poliovirus 1	KC880373	7442	06/02/2010	Human	C type
1436	RUS36815	Human poliovirus 1	KC880380	7442	06/08/2010	Human	C type

1437	RUS37255	Human poliovirus 1	KC880378	7442	06/09/2010	Human	C type
1438	RUS39223	Human poliovirus 1	KC880365	7442	11/22/2010	Human	C type
1439	RUS39270	Human poliovirus 1	KC880370	7442	11/22/2010	Human	C type
1440	Sabin 1	Human poliovirus 1	AY184219	7441	-N/A-	Human	C type
1441	Sabin 1	Human poliovirus 1	GQ984141	7441	-N/A-	Human	C type
1442	Sabin 1	Human poliovirus 1	V01150	7441	-N/A-	Human	C type
1443	TCDC01-113	Human poliovirus 1	AF538841	7478	-N/A-	Human	C type
1444	TCDC01-330	Human poliovirus 1	AF538842	7456	-N/A-	Human	C type
1445	TCDC01-861	Human poliovirus 1	AF538843	7445	-N/A-	Human	C type
1446	TCDCE01-135	Human poliovirus 1	AF538840	7443	-N/A-	Human	C type
1447	TJK35325	Human poliovirus 1	KC880372	7442	03/28/2010	Human	C type
1448	TJK35363	Human poliovirus 1	KC880366	7442	04/10/2010	Human	C type
1449	TJK35363	Human poliovirus 1	KC880367	7442	04/10/2010	Human	C type
1450	TJK35363	Human poliovirus 1	KC880368	7442	04/10/2010	Human	C type
1451	TJK35363	Human poliovirus 1	KC880369	7442	04/10/2010	Human	C type
1452	TJK35389	Human poliovirus 1	KC88037	7442	02/01/2	Human	C type

			5		010		
1453	TJK35389	Human poliovirus 1	KC880376	7442	02/01/2010	Human	C type
1454	TJK35453	Human poliovirus 1	KC880379	7442	04/26/2010	Human	C type
1455	TJK36567	Human poliovirus 1	KC880377	7442	05/14/2010	Human	C type
1456	TJK37339	Human poliovirus 1	KC880381	7442	07/04/2010	Human	C type
1457	TKM37184	Human poliovirus 1	KC880374	7442	06/19/2010	Human	C type
1458	USA10771	Human poliovirus 1	EF682349	7415	-N/A-	Human	C type
1459	USA10772	Human poliovirus 1	EF682350	7415	-N/A-	Human	C type
1460	USA10773	Human poliovirus 1	EF682345	7415	-N/A-	Human	C type
1461	USA10774	Human poliovirus 1	EF682343	7415	-N/A-	Human	C type
1462	USA10775	Human poliovirus 1	EF682344	7415	-N/A-	Human	C type
1463	USA10776	Human poliovirus 1	EF682355	7415	-N/A-	Human	C type
1464	USA10777	Human poliovirus 1	EF682353	7415	-N/A-	Human	C type
1465	USA10778	Human poliovirus 1	EF682351	7415	-N/A-	Human	C type
1466	USA10779	Human poliovirus 1	EF682354	7415	-N/A-	Human	C type
1467	USA10780	Human poliovirus 1	EF682346	7415	-N/A-	Human	C type
1468	USA10781	Human poliovirus 1	EF682347	7415	-N/A-	Human	C type
1469	USA10782	Human poliovirus 1	EF682348	7415	-N/A-	Human	C type

1470	USA10783	Human poliovirus 1	EF682358	7441	-N/A-	Human	C type
1471	USA10784	Human poliovirus 1	EF682356	7441	-N/A-	Human	C type
1472	USA10785	Human poliovirus 1	EF682357	7441	-N/A-	Human	C type
1473	USA10786	Human poliovirus 1	EF682359	7441	-N/A-	Human	C type
1474	Mahoney	Human poliovirus 1 Mahoney	V01149	7440	-N/A-	Human	C type
1475	10	Human poliovirus 2	MG212488	7435	-N/A-	Human	C type
1476	102050	Human poliovirus 2	AJ544513	7439	-N/A-	Human	C type
1477	10630	Human poliovirus 2	MG212469	7454	1999	Human	C type
1478	12209	Human poliovirus 2	MG212465	7441	1999	Human	C type
1479	12604	Human poliovirus 2	MG212486	7460	2000	Human	C type
1480	13770	Human poliovirus 2	MG212484	7459	2000	Human	C type
1481	14304	Human poliovirus 2	MG212464	7440	2001	Human	C type
1482	14337	Human poliovirus 2	MG212463	7455	2001	Human	C type
1483	14732	Human poliovirus 2	MG212462	7441	2001	Human	C type
1484	15763_T2	Human poliovirus 2	MG212459	7456	2001	Human	C type
1485	15767_T2_c0	Human poliovirus 2	MG21245	7457	2001	Human	C type

			7				
1486	16849	Human poliovirus 2	MG212450	7437	2002	Human	C type
1487	17629	Human poliovirus 2	MG212456	7454	2002	Human	C type
1488	17719_T2	Human poliovirus 2	MG212487	7460	2002	Human	C type
1489	18058	Human poliovirus 2	MG212480	7459	2002	Human	C type
1490	18580	Human poliovirus 2	MG212455	7450	2002	Human	C type
1491	19062	Human poliovirus 2	MG212454	7457	2002	Human	C type
1492	19890	Human poliovirus 2	MG212476	7459	-N/A-	Human	C type
1493	21348_T2	Human poliovirus 2	MG212475	7457	2004	Human	C type
1494	29425_c0	Human poliovirus 2	MG212435	7442	-N/A-	Human	C type
1495	29425_c11	Human poliovirus 2	MG212434	7442	-N/A-	Human	C type
1496	29683_c0	Human poliovirus 2	MG212452	7456	-N/A-	Human	C type
1497	29683_c24	Human poliovirus 2	MG212453	7456	-N/A-	Human	C type
1498	31947	Human poliovirus 2	MG21244	7438	-N/A-	Human	C type

			0				
1499	31996	Human poliovirus 2	FJ460225	7434	2000	Human	C type
1500	32189+AP1	Human poliovirus 2	FJ460224	7434	2000	Human	C type
1501	32191	Human poliovirus 2	FJ460223	7446	2000	Human	C type
1502	44624/BRA2014	Human poliovirus 2	KU37265 2	7439	2014	Human	C type
1503	6M_V	Human poliovirus 2	MG21242 8	7439	-N/A-	Human	C type
1504	7	Human poliovirus 2	MG21248 9	7460	-N/A-	Human	C type
1505	9112	Human poliovirus 2	MG21247 1	7451	1998	Human	C type
1506	9632_T2	Human poliovirus 2	MG21244 6	7445	1999	Human	C type
1507	CHN1054	Human poliovirus 2	HM10783 2	7439	03/23/1 997	Human	C type
1508	CHN1078	Human poliovirus 2	HM10783 3	7439	05/06/1 997	Human	C type
1509	CHN15261/Sichuan/CHN/2011	Human poliovirus 2	KJ419273	7439	09/20/2 011	Human	C type
1510	CHN15284/Sichuan/CHN/2011	Human poliovirus 2	KJ419274	7439	08/18/2 011	Human	C type
1511	CHN16003/Sichuan/CHN/2012	Human poliovirus 2	KJ419275	7439	01/19/2 012	Human	C type
1512	CHN16017/Sichuan/CHN/2012	Human poliovirus 2	KJ419276	7439	02/23/2 012	Human	C type

1513	CHN16019c/Sichuan/CHN/2012	Human poliovirus 2	KJ419277	7439	02/08/2012	Human	C type
1514	CHN18046/Shanxi/CHN/2014	Human poliovirus 2	KP196613	7439	05/01/2014	Human	C type
1515	CHN3219	Human poliovirus 2	HM107834	7439	07/30/1999	Human	C type
1516	CHN8316	Human poliovirus 2	HM107835	7439	08/18/2004	Human	C type
1517	CSF16/YN/CHN/2013	Human poliovirus 2	KM433732	7438	05/14/2013	Human	C type
1518	E12-E12221/SD/CHN/sewage	Human poliovirus 2	KF656732	7439	12/03/2012	Human	C type
1519	EGY88-074	Human poliovirus 2	AF448782	7439	-N/A-	Human	C type
1520	EGY93-034	Human poliovirus 2	AF448783	7441	-N/A-	Human	C type
1521	Env2008_E2450	Human poliovirus 2	KC784367	7448	09/25/2008	Human	C type
1522	Env2008_E3218	Human poliovirus 2	KC784368	7439	11/16/2009	Human	C type
1523	IRQ1219537	Human poliovirus 2	KY941932	7436	2012	Human	C type
1524	IS_001	Human poliovirus 2	MG212490	7452	-N/A-	Human	C type
1525	IS_061	Human poliovirus 2	MG212491	7428	-N/A-	Human	C type
1526	Lansing	Human poliovirus 2	M12197	7440	-N/A-	Human	C type
1527	MAD004	Human poliovirus 2	AM08422	7439	-N/A-	Human	C type

			3				
1528	MAD007	Human poliovirus 2	AM084224	7439	-N/A-	Human	C type
1529	MAD029	Human poliovirus 2	AM084225	7439	-N/A-	Human	C type
1530	MEF-1	Human poliovirus 2	AY238473	7440	-N/A-	Human	C type
1531	NIE0110767	Human poliovirus 2	DQ890386	7441	-N/A-	Human	C type
1532	NIE0210766	Human poliovirus 2	DQ890385	7439	-N/A-	Human	C type
1533	NIE0511436	Human poliovirus 2	JX274980	7439	2005	Human	C type
1534	NIE0511440	Human poliovirus 2	JX274981	7439	2005	Human	C type
1535	NIE0511445	Human poliovirus 2	JX274983	7439	2005	Human	C type
1536	NIE0511448	Human poliovirus 2	JX274982	7439	2005	Human	C type
1537	NIE0611195	Human poliovirus 2	JX274985	7434	2006	Human	C type
1538	NIE0611195	Human poliovirus 2	KX162696	7414	05/19/2006	Human	C type
1539	NIE0611197	Human poliovirus 2	JX274995	7438	2006	Human	C type
1540	NIE0611200	Human poliovirus 2	JX274991	7439	2006	Human	C type
1541	NIE0611432	Human poliovirus 2	KX162697	7422	06/06/2006	Human	C type
1542	NIE0611444	Human poliovirus 2	JX274984	7439	2006	Human	C type
1543	NIE0611450	Human poliovirus 2	JX275015	7439	2006	Human	C type

1544	NIE0611451	Human poliovirus 2	JX274999	7439	2006	Human	C type
1545	NIE0611452	Human poliovirus 2	JX275008	7439	2006	Human	C type
1546	NIE0611452	Human poliovirus 2	KX162699	7422	10/17/2006	Human	C type
1547	NIE0611517	Human poliovirus 2	KX162700	7421	10/06/2006	Human	C type
1548	NIE0611522	Human poliovirus 2	KX162701	7422	10/27/2006	Human	C type
1549	NIE0611523	Human poliovirus 2	KX162702	7414	09/16/2006	Human	C type
1550	NIE0611531	Human poliovirus 2	KX162703	7422	09/14/2006	Human	C type
1551	NIE0611534	Human poliovirus 2	KX162704	7417	10/26/2006	Human	C type
1552	NIE0611535	Human poliovirus 2	KX162705	7414	10/10/2006	Human	C type
1553	NIE0611579	Human poliovirus 2	KX162706	7245	08/03/2006	Human	C type
1554	NIE0711199	Human poliovirus 2	JX275032	7442	2007	Human	C type
1555	NIE0711438	Human poliovirus 2	JX275085	7440	2007	Human	C type
1556	NIE0711441	Human poliovirus 2	JX275071	7442	2007	Human	C type
1557	NIE0811454	Human poliovirus 2	JX275162	7425	2008	Human	C type
1558	NIE0811456	Human poliovirus 2	JX275140	7442	2008	Human	C type
1559	NIE0811460	Human poliovirus 2	JX275147	7439	2008	Human	C type

1560	NIE0911443	Human poliovirus 2	JX275266	7440	2009	Human	C type
1561	NIE0911455	Human poliovirus 2	JX275184	7440	2009	Human	C type
1562	NIE0911458	Human poliovirus 2	JX275238	7440	2009	Human	C type
1563	NIE0918441	Human poliovirus 2	KJ170569	7387	2009	Human	C type
1564	NIE0918442	Human poliovirus 2	KJ170573	7387	2009	Human	C type
1565	NIE1018408	Human poliovirus 2	KJ170549	7387	2010	Human	C type
1566	NIE1018409	Human poliovirus 2	KJ170572	7387	2010	Human	C type
1567	NIE1018410	Human poliovirus 2	KJ170563	7387	2010	Human	C type
1568	NIE1018411	Human poliovirus 2	KJ170538	7387	2010	Human	C type
1569	NIE1018412	Human poliovirus 2	KJ170537	7387	2010	Human	C type
1570	NIE1018413	Human poliovirus 2	KJ170548	7387	2010	Human	C type
1571	NIE1018414	Human poliovirus 2	KJ170558	7387	2010	Human	C type
1572	NIE1018420	Human poliovirus 2	KJ170533	7387	2010	Human	C type
1573	NIE1018421	Human poliovirus 2	KJ170567	7387	2010	Human	C type
1574	NIE1018422	Human poliovirus 2	KJ170540	7387	2010	Human	C type
1575	NIE1018423	Human poliovirus 2	KJ170543	7387	2010	Human	C type
1576	NIE1018430	Human poliovirus 2	KJ170556	7387	2010	Human	C type
1577	NIE1018443	Human poliovirus 2	KJ170539	7387	2010	Human	C type
1578	NIE1018444	Human poliovirus 2	KJ170546	7387	2010	Human	C type
1579	NIE1018445	Human poliovirus 2	KJ170547	7387	2010	Human	C type
1580	NIE1018446	Human poliovirus 2	KJ170554	7387	2010	Human	C type

1581	NIE1116152	Human poliovirus 2	JX275352	7440	2011	Human	C type
1582	NIE1116178	Human poliovirus 2	JX275380	7440	2011	Human	C type
1583	NIE1116178	Human poliovirus 2	KX16270 7	7399	11/22/20 11	Human	C type
1584	NIE1118415	Human poliovirus 2	KJ170557	7387	2011	Human	C type
1585	NIE1118416	Human poliovirus 2	KJ170544	7387	2011	Human	C type
1586	NIE1118424	Human poliovirus 2	KJ170534	7387	2011	Human	C type
1587	NIE1118425	Human poliovirus 2	KJ170551	7387	2011	Human	C type
1588	NIE1118426	Human poliovirus 2	KJ170568	7387	2011	Human	C type
1589	NIE1118427	Human poliovirus 2	KJ170535	7387	2011	Human	C type
1590	NIE1118428	Human poliovirus 2	KJ170536	7387	2011	Human	C type
1591	NIE1118429	Human poliovirus 2	KJ170552	7387	2011	Human	C type
1592	NIE1118431	Human poliovirus 2	KJ170555	7387	2011	Human	C type
1593	NIE1118432	Human poliovirus 2	KJ170541	7387	2011	Human	C type
1594	NIE1118433	Human poliovirus 2	KJ170564	7387	2011	Human	C type
1595	NIE1118434	Human poliovirus 2	KJ170559	7387	2011	Human	C type
1596	NIE1118435	Human poliovirus 2	KJ170553	7387	2011	Human	C type
1597	NIE1118436	Human poliovirus 2	KJ170542	7387	2011	Human	C type
1598	NIE1118437	Human poliovirus 2	KJ170565	7387	2011	Human	C type
1599	NIE1118438	Human poliovirus 2	KJ170571	7387	2011	Human	C type
1600	NIE1118439	Human poliovirus 2	KJ170550	7387	2011	Human	C type

1601	NIE1118440	Human poliovirus 2	KJ170560	7387	2011	Human	C type
1602	NIE1118447	Human poliovirus 2	KJ170566	7387	2011	Human	C type
1603	NIE1118448	Human poliovirus 2	KJ170545	7387	2011	Human	C type
1604	NIE1119343	Human poliovirus 2	KX162710	7331	10/18/2011	Human	C type
1605	NIE1119344	Human poliovirus 2	KX162711	7357	10/24/2011	Human	C type
1606	NIE1218417	Human poliovirus 2	KJ170562	7387	2012	Human	C type
1607	NIE1218418	Human poliovirus 2	KJ170561	7387	2012	Human	C type
1608	NIE1218419	Human poliovirus 2	KJ170575	7387	2012	Human	C type
1609	NIE1218449	Human poliovirus 2	KJ170574	7387	2012	Human	C type
1610	NIE1218450	Human poliovirus 2	KJ170570	7387	2012	Human	C type
1611	NIE1419321	Human poliovirus 2	KX162712	7421	11/20/2014	Human	C type
1612	NIE1419323	Human poliovirus 2	KX162713	7429	11/13/2014	Human	C type
1613	NIE1519322	Human poliovirus 2	KX162714	7440	01/20/2015	Human	C type
1614	NIE1519324	Human poliovirus 2	KX162715	7422	02/09/2015	Human	C type
1615	NIE1519325	Human poliovirus 2	KX162716	7439	02/09/2015	Human	C type
1616	NIE1519342	Human poliovirus 2	KX162717	7398	08/10/2015	Human	C type

1617	P2S/Mog65-1 (20003)	Human poliovirus 2	AY278550	7439	-N/A-	Human	C type
1618	P2S/Mog65-2 (20077)	Human poliovirus 2	AY278552	7439	-N/A-	Human	C type
1619	P2S/Mog65-3 (20120)	Human poliovirus 2	AY278549	7439	-N/A-	Human	C type
1620	P2S/Mog66-4 (21043)	Human poliovirus 2	AY278551	7439	-N/A-	Human	C type
1621	PER8310769	Human poliovirus 2	DQ89038 8	7439	-N/A-	Human	C type
1622	PV2/4568-1/ISR98	Human poliovirus 2	AM04003 5	7312	-N/A-	Human	C type
1623	PV2/5021-1/ISR99	Human poliovirus 2	AM04003 6	7312	-N/A-	Human	C type
1624	PV2/5074-18/ISR99	Human poliovirus 2	AM04003 7	7312	-N/A-	Human	C type
1625	PV2/5104-1/ISR99	Human poliovirus 2	AM04003 8	7312	-N/A-	Human	C type
1626	PV2/5116-9/ISR99	Human poliovirus 2	AM04003 9	7312	-N/A-	Human	C type
1627	PV2/Bel	Human poliovirus 2	FJ517648	7418	05/2007	Human	C type
1628	PV2/Rus	Human poliovirus 2	FJ517649	7419	02/2008	Human	C type
1629	R93150	Human poliovirus 2	KR25935 6	7427	2014	Human	C type
1630	R93152	Human poliovirus 2	KR25935 7	7427	2014	Human	C type
1631	RF33/YN/CHN/2010	Human poliovirus 2	MN38443 8	7432	10/30/2 010	Human	C type
1632	Sabin	Human poliovirus 2	EU56693	7438	-N/A-	Human	C type

			4				
1633	Sabin	Human poliovirus 2	EU56693<br>5	7438	-N/A-	Human	C type
1634	Sabin	Human poliovirus 2	EU56693<br>6	7438	-N/A-	Human	C type
1635	Sabin	Human poliovirus 2	EU56693<br>7	7438	-N/A-	Human	C type
1636	Sabin	Human poliovirus 2	EU56693<br>8	7438	-N/A-	Human	C type
1637	Sabin	Human poliovirus 2	EU56693<br>9	7438	-N/A-	Human	C type
1638	Sabin	Human poliovirus 2	EU56694<br>0	7438	-N/A-	Human	C type
1639	Sabin	Human poliovirus 2	EU56694<br>1	7438	-N/A-	Human	C type
1640	Sabin	Human poliovirus 2	EU56694<br>2	7438	-N/A-	Human	C type
1641	Sabin	Human poliovirus 2	EU56694<br>3	7438	-N/A-	Human	C type
1642	Sabin	Human poliovirus 2	EU56694<br>4	7438	-N/A-	Human	C type
1643	Sabin	Human poliovirus 2	EU56694<br>5	7438	-N/A-	Human	C type
1644	Sabin	Human poliovirus 2	EU56694<br>6	7438	-N/A-	Human	C type
1645	Sabin	Human poliovirus 2	EU56694	7438	-N/A-	Human	C type

			7					
1646	Sabin	Human poliovirus 2	EU566948	7438	-N/A-	Human	C type
1647	Sabin	Human poliovirus 2	EU566949	7438	-N/A-	Human	C type
1648	Sabin	Human poliovirus 2	EU566950	7438	-N/A-	Human	C type
1649	Sabin 2	Human poliovirus 2	AY184220	7439	-N/A-	Human	C type
1650	Sabine 2	Human poliovirus 2	FJ898290	7439	01/19/1999	Human	C type
1651	T08-090	Human poliovirus 2	JX569709	7439	2008	Human	C type
1652	T09-216	Human poliovirus 2	JX569712	7438	2009	Human	C type
1653	T10-259	Human poliovirus 2	JX569711	7438	2010	Human	C type
1654	Human-AY177685	Human poliovirus 2	AY177685	7439	-N/A-	Human	C type
1655	Human-CS406482	Human poliovirus 2	CS406482	7439	-N/A-	Human	C type
1656	Human-CS406483	Human poliovirus 2	CS406483	7439	-N/A-	Human	C type
1657	Human-DQ205099	Human poliovirus 2	DQ205099	7439	-N/A-	Human	C type
1658	Human-MZ245455	Human poliovirus 2	MZ245455	7500	2015	Human	C type
1659	Human-X00595	Human poliovirus 2	X00595	7439	-N/A-	Human	C type
1660	USA0911201	Human poliovirus 2	GU390707	7437	03/03/2009	Human	C type

1661	USA9810768	Human poliovirus 2	DQ890387	7439	-N/A-	Human	C type
1662	VDPV MAD005	Human poliovirus 2	AM884184	7439	2002	Human	C type
1663	VDPV MAD006	Human poliovirus 2	AM884185	7439	2002	Human	C type
1664	W-2	Human poliovirus 2	D00625	7434	-N/A-	Human	C type
1665	Human-MN654096	Human poliovirus 2 nOPV2-CD	MN654096	7439	-N/A-	Human	C type
1666	10073	Human poliovirus 3	MG212470	7442	1999	Human	C type
1667	10664	Human poliovirus 3	MG212468	7443	1999	Human	C type
1668	11264	Human poliovirus 3	MG212467	7440	1999	Human	C type
1669	11948	Human poliovirus 3	MG212466	7443	2000	Human	C type
1670	14385	Human poliovirus 3	MG212483	7448	2001	Human	C type
1671	14770	Human poliovirus 3	MG212461	7440	2001	Human	C type
1672	14829	Human poliovirus 3	MG212460	7447	2001	Human	C type
1673	14984	Human poliovirus 3	MG212448	7424	-N/A-	Human	C type
1674	15762	Human poliovirus 3	MG21243	7443	1999	Human	C type

			1				
1675	15763_T3	Human poliovirus 3	MG212458	7448	2001	Human	C type
1676	15767_T3	Human poliovirus 3	MG212432	7425	2001	Human	C type
1677	15770	Human poliovirus 3	MG212449	7430	-N/A-	Human	C type
1678	16204	Human poliovirus 3	MG212482	7448	2002	Human	C type
1679	16938	Human poliovirus 3	MG212481	7446	2002	Human	C type
1680	17719_T3	Human poliovirus 3	MG212445	7440	2002	Human	C type
1681	17723	Human poliovirus 3	MG212451	7427	2002	Human	C type
1682	18963	Human poliovirus 3	MG212478	7448	2002	Human	C type
1683	19017	Human poliovirus 3	MG212477	7449	2002	Human	C type
1684	21348_T3	Human poliovirus 3	MG212474	7442	2004	Human	C type
1685	21390	Human poliovirus 3	MG212473	7459	2004	Human	C type
1686	23127	Human poliovirus 3	X04468	7435	-N/A-	Human	C type
1687	23_S2_P_6-4	Human poliovirus 3	MG212492	7430	-N/A-	Human	C type

1688	29813	Human poliovirus 3	MG212439	7431	-N/A-	Human	C type
1689	2K_P9	Human poliovirus 3	MG212427	7431	-N/A-	Human	C type
1690	31974	Human poliovirus 3	FJ460227	7330	2000	Human	C type
1691	32335_c0	Human poliovirus 3	MG212444	7424	-N/A-	Human	C type
1692	32335_c17	Human poliovirus 3	MG212441	7425	-N/A-	Human	C type
1693	32335_c19	Human poliovirus 3	MG212447	7425	-N/A-	Human	C type
1694	32335_c5	Human poliovirus 3	MG212442	7424	-N/A-	Human	C type
1695	32335_c8	Human poliovirus 3	MG212443	7425	-N/A-	Human	C type
1696	33239	Human poliovirus 3	FJ460226	7432	2000	Human	C type
1697	36_S2_P_7-3	Human poliovirus 3	MG212493	7432	-N/A-	Human	C type
1698	45507	Human poliovirus 3	KU763188	7431	01/2015	Human	C type
1699	9633_T3	Human poliovirus 3	MG212430	7429	1999	Human	C type
1700	CHN6218/GS/CHN/2002	Human poliovirus 3	FJ859187	7432	07/03/2002	Human	C type
1701	CHN6356/GZ/CHN/2002	Human poliovirus 3	FJ859188	7432	09/10/2002	Human	C type

1702	Env08_E2886	Human poliovirus 3	KC784372	7422	12/29/2008	Human	C type
1703	Env_2016_Sep_PV3	Human poliovirus 3	MG451807	7381	09/08/2016	Human	C type
1704	Env_2017_Jan_PV-3	Human poliovirus 3	MG451811	6695	01/10/2017	Human	C type
1705	FIN84-2493	Human poliovirus 3	FJ842159	7398	1984	Human	C type
1706	FIN84-60212	Human poliovirus 3	FJ842158	7416	1984	Human	C type
1707	IRA10852	Human poliovirus 3	EU684057	7432	12/18/2006	Human	C type
1708	IRA10853	Human poliovirus 3	EU684056	7432	12/21/2006	Human	C type
1709	MB1	Human poliovirus 3	GU180608	7432	-N/A-	Human	C type
1710	NIE0918451	Human poliovirus 3	KJ170642	7379	2009	Human	C type
1711	NIE0918452	Human poliovirus 3	KJ170662	7379	2009	Human	C type
1712	NIE0918501	Human poliovirus 3	KJ170670	7379	2009	Human	C type
1713	NIE0918514	Human poliovirus 3	KJ170664	7379	2009	Human	C type
1714	NIE0918515	Human poliovirus 3	KJ170673	7379	2009	Human	C type
1715	NIE0918516	Human poliovirus 3	KJ170674	7379	2009	Human	C type
1716	NIE0918517	Human poliovirus 3	KJ170663	7379	2009	Human	C type
1717	NIE0918518	Human poliovirus 3	KJ170669	7379	2009	Human	C type
1718	NIE0918519	Human poliovirus 3	KJ170676	7379	2009	Human	C type

1719	NIE1018453	Human poliovirus 3	KJ170677	7379	2010	Human	C type
1720	NIE1018454	Human poliovirus 3	KJ170640	7379	2010	Human	C type
1721	NIE1018457	Human poliovirus 3	KJ170590	7379	2010	Human	C type
1722	NIE1018458	Human poliovirus 3	KJ170636	7379	2010	Human	C type
1723	NIE1018459	Human poliovirus 3	KJ170624	7379	2010	Human	C type
1724	NIE1018460	Human poliovirus 3	KJ170666	7379	2010	Human	C type
1725	NIE1018480	Human poliovirus 3	KJ170588	7379	2010	Human	C type
1726	NIE1018481	Human poliovirus 3	KJ170580	7379	2010	Human	C type
1727	NIE1018482	Human poliovirus 3	KJ170607	7379	2010	Human	C type
1728	NIE1018483	Human poliovirus 3	KJ170581	7379	2010	Human	C type
1729	NIE1018484	Human poliovirus 3	KJ170638	7379	2010	Human	C type
1730	NIE1018485	Human poliovirus 3	KJ170671	7379	2010	Human	C type
1731	NIE1018486	Human poliovirus 3	KJ170634	7379	2010	Human	C type
1732	NIE1018487	Human poliovirus 3	KJ170596	7379	2010	Human	C type
1733	NIE1018488	Human poliovirus 3	KJ170591	7379	2010	Human	C type
1734	NIE1018489	Human poliovirus 3	KJ170582	7379	2010	Human	C type
1735	NIE1018490	Human poliovirus 3	KJ170660	7379	2010	Human	C type
1736	NIE1018491	Human poliovirus 3	KJ170632	7379	2010	Human	C type
1737	NIE1018492	Human poliovirus 3	KJ170594	7379	2010	Human	C type
1738	NIE1018493	Human poliovirus 3	KJ170639	7379	2010	Human	C type
1739	NIE1018502	Human poliovirus 3	KJ170600	7379	2010	Human	C type

1740	NIE1018503	Human poliovirus 3	KJ170602	7379	2010	Human	C type
1741	NIE1018525	Human poliovirus 3	KJ170672	7379	2010	Human	C type
1742	NIE1118455	Human poliovirus 3	KJ170577	7379	2011	Human	C type
1743	NIE1118456	Human poliovirus 3	KJ170618	7379	2011	Human	C type
1744	NIE1118468	Human poliovirus 3	KJ170593	7379	2011	Human	C type
1745	NIE1118469	Human poliovirus 3	KJ170648	7379	2011	Human	C type
1746	NIE1118470	Human poliovirus 3	KJ170649	7379	2011	Human	C type
1747	NIE1118471	Human poliovirus 3	KJ170651	7379	2011	Human	C type
1748	NIE1118472	Human poliovirus 3	KJ170652	7379	2011	Human	C type
1749	NIE1118473	Human poliovirus 3	KJ170653	7379	2011	Human	C type
1750	NIE1118474	Human poliovirus 3	KJ170644	7379	2011	Human	C type
1751	NIE1118475	Human poliovirus 3	KJ170587	7379	2011	Human	C type
1752	NIE1118476	Human poliovirus 3	KJ170654	7379	2011	Human	C type
1753	NIE1118477	Human poliovirus 3	KJ170645	7379	2011	Human	C type
1754	NIE1118478	Human poliovirus 3	KJ170592	7379	2011	Human	C type
1755	NIE1118508	Human poliovirus 3	KJ170603	7379	2011	Human	C type
1756	NIE1118509	Human poliovirus 3	KJ170656	7379	2011	Human	C type
1757	NIE1118510	Human poliovirus 3	KJ170659	7379	2011	Human	C type
1758	NIE1118511	Human poliovirus 3	KJ170619	7379	2011	Human	C type
1759	NIE1118512	Human poliovirus 3	KJ170579	7379	2011	Human	C type
1760	NIE1118513	Human poliovirus 3	KJ170661	7379	2011	Human	C type

1761	NIE1118524	Human poliovirus 3	KJ170609	7379	2011	Human	C type
1762	NIE1118526	Human poliovirus 3	KJ170646	7379	2011	Human	C type
1763	NIE1118527	Human poliovirus 3	KJ170650	7379	2011	Human	C type
1764	NIE1118528	Human poliovirus 3	KJ170647	7379	2011	Human	C type
1765	NIE1118529	Human poliovirus 3	KJ170643	7379	2011	Human	C type
1766	NIE1118530	Human poliovirus 3	KJ170658	7379	2011	Human	C type
1767	NIE1118531	Human poliovirus 3	KJ170665	7379	2011	Human	C type
1768	NIE1118532	Human poliovirus 3	KJ170641	7379	2011	Human	C type
1769	NIE1118533	Human poliovirus 3	KJ170657	7379	2011	Human	C type
1770	NIE1118534	Human poliovirus 3	KJ170637	7379	2011	Human	C type
1771	NIE1118535	Human poliovirus 3	KJ170675	7379	2011	Human	C type
1772	NIE1218536	Human poliovirus 3	KJ170605	7379	2012	Human	C type
1773	NIE1218537	Human poliovirus 3	KJ170589	7379	2012	Human	C type
1774	NIE1218538	Human poliovirus 3	KJ170625	7379	2012	Human	C type
1775	NIE1218539	Human poliovirus 3	KJ170606	7379	2012	Human	C type
1776	NIE1218547	Human poliovirus 3	KJ170585	7379	2012	Human	C type
1777	NIE1218548	Human poliovirus 3	KJ170586	7379	2012	Human	C type
1778	NIE1218549	Human poliovirus 3	KJ170633	7379	2012	Human	C type
1779	NIE1218550	Human poliovirus 3	KJ170622	7379	2012	Human	C type
1780	NIE1218551	Human poliovirus 3	KJ170623	7379	2012	Human	C type
1781	NIE1218552	Human poliovirus 3	KJ170667	7379	2012	Human	C type

1782	NIE1219535	Human poliovirus 3	KY941933	7435	2012	Human	C type
1783	P3/Jinan/1/09	Human poliovirus 3	GU256222	7432	05/28/2009	Human	C type
1784	PAK1019536	Human poliovirus 3	KY941934	7435	2010	Human	C type
1785	PV3/NOR/01/8	Human poliovirus 3	AF541919	7419	-N/A-	Human	C type
1786	R1351/YN/CHN/2013	Human poliovirus 3	MK028134	7430	10/30/2013	Human	C type
1787	R46064	Human poliovirus 3	KR259358	7420	2008	Human	C type
1788	RF108/YN/CHN/2010	Human poliovirus 3	KT946714	7402	03/30/2010	Human	C type
1789	RF134/YN/CHN/2010	Human poliovirus 3	KT946715	7402	04/17/2010	Human	C type
1790	RF146/YN/CHN/2010	Human poliovirus 3	KT946716	7402	04/28/2010	Human	C type
1791	RF151/YN/CHN/2010	Human poliovirus 3	KT946717	7402	05/01/2010	Human	C type
1792	RUS-14039030003-1	Human poliovirus 3	MT645947	7430	07/11/2014	Human	C type
1793	RUS-14039030003-2	Human poliovirus 3	MT645948	7430	07/12/2014	Human	C type
1794	RUS-14039030003-K1	Human poliovirus 3	MT645949	7421	07/13/2014	Human	C type
1795	RUS-14039030003-K4	Human poliovirus 3	MT64595	7419	07/13/2	Human	C type

			0		014		
1796	RUS-14039030003-K5	Human poliovirus 3	MT645951	7430	07/13/2014	Human	C type
1797	S16PMR46022	Human poliovirus 3	MG212495	7427	-N/A-	Human	C type
1798	S20PMR46017	Human poliovirus 3	MG212494	7431	-N/A-	Human	C type
1799	Sabin	Human poliovirus 3	MF678293	6981	2005	Human	C type
1800	Sabin 3	Human poliovirus 3	AY184221	7432	-N/A-	Human	C type
1801	SWI10947	Human poliovirus 3	FJ914252	7442	1980	Human	C type
1802	4310900947_RD4	Enterovirus D	MN954540	7322	07/24/2009	Human	D type
1803	4311200821_RD3	Enterovirus D	MN954536	7351	10/08/2012	Human	D type
1804	4311201039_RD3	Enterovirus D	MN954539	7300	11/30/2012	Human	D type
1805	4311300117_RD4	Enterovirus D	MN954538	7343	01/15/2013	Human	D type
1806	4311400720_RD4	Enterovirus D	MN954537	7342	09/10/2014	Human	D type
1807	4311601013_RD4	Enterovirus D	MN954541	7343	09/16/2016	Human	D type
1808	Enterovirus 70	Enterovirus D	NC_001430 *	7390	-N/A-	Human	D type
1809	EV-D68/Homo sapiens/USA/SSENT01/2014	Enterovirus D	KX35182	7285	09/14/2	Human	D type

			7		014		
1810	EV-D68/Homo sapiens/USA/SSENT02/2014	Enterovirus D	KX35181 4	7285	09/12/2 014	Human	D type
1811	EV-D68/Homo sapiens/USA/SSENT03/2014	Enterovirus D	KX35179 8	7285	09/13/2 014	Human	D type
1812	EV-D68/Homo sapiens/USA/SSENT04/2014	Enterovirus D	KX35182 8	7285	09/14/2 014	Human	D type
1813	EV-D68/Homo sapiens/USA/SSENT05/2014	Enterovirus D	KX35182 4	7283	09/15/2 014	Human	D type
1814	EV-D68/Homo sapiens/USA/SSENT06/2014	Enterovirus D	KX35180 2	7282	09/16/2 014	Human	D type
1815	EV-D68/Homo sapiens/USA/SSENT07/2014	Enterovirus D	KX35182 0	7285	09/16/2 014	Human	D type
1816	EV-D68/Homo sapiens/USA/SSENT08/2014	Enterovirus D	KX351811	7273	09/17/2 014	Human	D type
1817	EV-D68/Homo sapiens/USA/SSENT09/2014	Enterovirus D	KX35181 6	7285	09/14/2 014	Human	D type
1818	EV-D68/Homo sapiens/USA/SSENT10/2014	Enterovirus D	KX35181 2	7285	09/19/2 014	Human	D type
1819	EV-D68/Homo sapiens/USA/SSENT11/2014	Enterovirus D	KX35179 7	7285	09/18/2 014	Human	D type
1820	EV-D68/Homo sapiens/USA/SSENT12/2014	Enterovirus D	KX35181 9	7283	09/17/2 014	Human	D type
1821	EV-D68/Homo sapiens/USA/SSENT14/2014	Enterovirus D	KX35182 9	7283	09/17/2 014	Human	D type
1822	EV-D68/Homo sapiens/USA/SSENT16/2014	Enterovirus D	KX35182	7285	09/19/2	Human	D type

			2		014		
1823	EV-D68/Homo sapiens/USA/SSENT17/2014	Enterovirus D	KX35181 3	7285	09/17/2 014	Human	D type
1824	EV-D68/Homo sapiens/USA/SSENT18/2014	Enterovirus D	KX35180 5	7283	09/18/2 014	Human	D type
1825	EV-D68/Homo sapiens/USA/SSENT19/2014	Enterovirus D	KX35181 0	7285	09/17/2 014	Human	D type
1826	EV-D68/Homo sapiens/USA/SSENT20/2014	Enterovirus D	KX35181 8	7282	09/17/2 014	Human	D type
1827	EV-D68/Homo sapiens/USA/SSENT21/2014	Enterovirus D	KX35179 6	7285	09/19/2 014	Human	D type
1828	EV-D68/Homo sapiens/USA/SSENT22/2014	Enterovirus D	KX35180 3	7275	09/19/2 014	Human	D type
1829	EV-D68/Homo sapiens/USA/SSENT23/2014	Enterovirus D	KX35182 3	7283	09/17/2 014	Human	D type
1830	EV-D68/Homo sapiens/USA/SSENT24/2014	Enterovirus D	KX35183 0	7285	09/18/2 014	Human	D type
1831	EV-D68/Homo sapiens/USA/SSENT25/2014	Enterovirus D	KX35180 8	7285	09/17/2 014	Human	D type
1832	EV-D68/Homo sapiens/USA/SSENT27/2014	Enterovirus D	KX35179 5	7283	09/20/2 014	Human	D type
1833	EV-D68/Homo sapiens/USA/SSENT28/2014	Enterovirus D	KX35182 5	7283	09/20/2 014	Human	D type
1834	EV-D68/Homo sapiens/USA/SSENT29/2014	Enterovirus D	KX35179 4	7285	09/21/2 014	Human	D type
1835	EV-D68/Homo sapiens/USA/SSENT30/2014	Enterovirus D	KX35179	7282	09/23/2	Human	D type

			9		014		
1836	EV-D68/Homo sapiens/USA/SSENT31/2014	Enterovirus D	KX35180<br>0	7285	09/22/2<br>014	Human	D type
1837	EV-D68/Homo sapiens/USA/SSENT32/2014	Enterovirus D	KX35180<br>7	7282	09/24/2<br>014	Human	D type
1838	EV-D68/Homo sapiens/USA/SSENT33/2014	Enterovirus D	KX35181<br>5	7285	09/22/2<br>014	Human	D type
1839	EV-D68/Homo sapiens/USA/SSENT35/2014	Enterovirus D	KX35181<br>7	7285	09/21/2<br>014	Human	D type
1840	EV-D68/Homo sapiens/USA/SSENT36/2014	Enterovirus D	KX35180<br>6	7285	09/28/2<br>014	Human	D type
1841	EV-D68/Homo sapiens/USA/SSENT37/2014	Enterovirus D	KX35180<br>1	7285	09/28/2<br>014	Human	D type
1842	EV-D68/Homo sapiens/USA/SSENT38/2014	Enterovirus D	KX35180<br>4	7283	10/03/2<br>014	Human	D type
1843	EV-D68/Homo sapiens/USA/SSENT39/2014	Enterovirus D	KX35182<br>1	7284	09/18/2<br>014	Human	D type
1844	2011-21186	Enterovirus D68	KT280503	7332	11/15/20<br>11	Human	D type
1845	2011-21282	Enterovirus D68	KT285320	7332	12/19/2<br>011	Human	D type
1846	2011-21286	Enterovirus D68	KT306743	7347	12/20/2<br>011	Human	D type
1847	2012-12225	Enterovirus D68	KT285319	7348	01/18/2<br>012	Human	D type
1848	2013-0720-6	Enterovirus D68	KT280504	7332	07/20/2	Human	D type

					013		
1849	2013-0825-6	Enterovirus D68	KT280502	7348	08/25/2013	Human	D type
1850	2013-1017-26	Enterovirus D68	KT280501	7348	10/17/2013	Human	D type
1851	2014-R0672	Enterovirus D68	KT280500	7332	09/20/2014	Human	D type
1852	2014-R1011	Enterovirus D68	KT280498	7332	10/20/2014	Human	D type
1853	2014-R1153	Enterovirus D68	KT280497	7332	10/20/2014	Human	D type
1854	2014-R1357	Enterovirus D68	KT280496	7332	11/19/2014	Human	D type
1855	2014-R970	Enterovirus D68	KT280499	7332	10/15/2014	Human	D type
1856	37-99	Enterovirus D68	EF107098	7333	-N/A-	Human	D type
1857	BCH895A	Enterovirus D68	KF726085	7348	10/06/2008	Human	D type
1858	Beijing-R0132	Enterovirus D68	KP240936	7332	2014	Human	D type
1859	BJ24	Enterovirus D68	KU242683	7326	12/30/2008	Human	D type
1860	CA/AFP/11-1767	Enterovirus D68	KM892501	7343	2013	Human	D type
1861	CA/RESP/10-786	Enterovirus D68	KM892500	7341	2013	Human	D type

1862	EV-D68/Haiti/1/2014	Enterovirus D68	KT266905	7332	12/01/2014	Human	D type
1863	EVD68/Homo sapiens/USA/MO1/2014	Enterovirus D68	KT347234	7285	2014	Human	D type
1864	EVD68/Homo sapiens/USA/MO10/2014	Enterovirus D68	KT347257	7285	2014	Human	D type
1865	EVD68/Homo sapiens/USA/MO11/2014	Enterovirus D68	KT347260	7285	2014	Human	D type
1866	EVD68/Homo sapiens/USA/MO12/2014	Enterovirus D68	KT347258	7285	2014	Human	D type
1867	EVD68/Homo sapiens/USA/MO13/2014	Enterovirus D68	KT347248	7285	2014	Human	D type
1868	EVD68/Homo sapiens/USA/MO14/2014	Enterovirus D68	KT347243	7285	2014	Human	D type
1869	EVD68/Homo sapiens/USA/MO15/2014	Enterovirus D68	KT347228	7285	2014	Human	D type
1870	EVD68/Homo sapiens/USA/MO16/2014	Enterovirus D68	KT347249	7285	2014	Human	D type
1871	EVD68/Homo sapiens/USA/MO17/2014	Enterovirus D68	KT347270	7285	2014	Human	D type
1872	EVD68/Homo sapiens/USA/MO18/2014	Enterovirus D68	KT347241	7285	2014	Human	D type
1873	EVD68/Homo sapiens/USA/MO19/2014	Enterovirus D68	KT347233	7285	2014	Human	D type
1874	EVD68/Homo sapiens/USA/MO2/2014	Enterovirus D68	KT347277	7285	2014	Human	D type
1875	EVD68/Homo sapiens/USA/MO20/2014	Enterovirus D68	KT347256	7285	2014	Human	D type
1876	EVD68/Homo sapiens/USA/MO21/2014	Enterovirus D68	KT347276	7285	2014	Human	D type
1877	EVD68/Homo sapiens/USA/MO22/2014	Enterovirus D68	KT347264	7285	2014	Human	D type
1878	EVD68/Homo sapiens/USA/MO23/2014	Enterovirus D68	KT347255	7285	2014	Human	D type
1879	EVD68/Homo sapiens/USA/MO24/2014	Enterovirus D68	KT347253	7285	2014	Human	D type
1880	EVD68/Homo sapiens/USA/MO25/2014	Enterovirus D68	KT347225	7285	2014	Human	D type
1881	EVD68/Homo sapiens/USA/MO26/2014	Enterovirus D68	KT347227	7285	2014	Human	D type

1882	EVD68/Homo sapiens/USA/MO27/2014	Enterovirus D68	KT347266	7285	2014	Human	D type
1883	EVD68/Homo sapiens/USA/MO28/2014	Enterovirus D68	KT347263	7285	2014	Human	D type
1884	EVD68/Homo sapiens/USA/MO3/2014	Enterovirus D68	KT347244	7285	2014	Human	D type
1885	EVD68/Homo sapiens/USA/MO30/2014	Enterovirus D68	KT347237	7285	2014	Human	D type
1886	EVD68/Homo sapiens/USA/MO49/2014	Enterovirus D68	KT347240	7285	2014	Human	D type
1887	EVD68/Homo sapiens/USA/MO5/2014	Enterovirus D68	KT347242	7285	2014	Human	D type
1888	EVD68/Homo sapiens/USA/MO50/2014	Enterovirus D68	KT347268	7285	2014	Human	D type
1889	EVD68/Homo sapiens/USA/MO51/2014	Enterovirus D68	KT347252	7285	2014	Human	D type
1890	EVD68/Homo sapiens/USA/MO53/2014	Enterovirus D68	KT347226	7285	2014	Human	D type
1891	EVD68/Homo sapiens/USA/MO54/2014	Enterovirus D68	KT347223	7285	2014	Human	D type
1892	EVD68/Homo sapiens/USA/MO56/2014	Enterovirus D68	KT347246	7285	2014	Human	D type
1893	EVD68/Homo sapiens/USA/MO57/2014	Enterovirus D68	KT347262	7285	2014	Human	D type
1894	EVD68/Homo sapiens/USA/MO58/2014	Enterovirus D68	KT347224	7285	2014	Human	D type
1895	EVD68/Homo sapiens/USA/MO59/2014	Enterovirus D68	KT347245	7285	2014	Human	D type
1896	EVD68/Homo sapiens/USA/MO6/2014	Enterovirus D68	KT347239	7285	2014	Human	D type
1897	EVD68/Homo sapiens/USA/MO60/2014	Enterovirus D68	KT347261	7285	2014	Human	D type
1898	EVD68/Homo sapiens/USA/MO7/2014	Enterovirus D68	KT347271	7285	2014	Human	D type
1899	EVD68/Homo sapiens/USA/MO8/2014	Enterovirus D68	KT347278	7285	2014	Human	D type
1900	EVD68/Homo sapiens/USA/MO9/2014	Enterovirus D68	KT347259	7285	2014	Human	D type
1901	EVD68/Homo sapiens/USA/N0051U5/2012	Enterovirus D68	KT347280	7293	11/07/2012	Human	D type

1902	EVD68/Homo sapiens/XXX/ATCC VR-1197/XXXX	Enterovirus D68	KT725431	7319	-N/A-	Human	D type
1903	Fermon	Enterovirus D68	AY426531	7367	-N/A-	Human	D type
1904	MEX/DF/2014-InDRE2351	Enterovirus D68	KT825142	7321	10/23/2014	Human	D type
1905	MEX/DGO/2014-InDRE2271	Enterovirus D68	KT803995	7321	10/16/2014	Human	D type
1906	NY120	Enterovirus D68	KP745751	7332	09/28/2014	Human	D type
1907	NY153	Enterovirus D68	KP745755	7332	10/02/2014	Human	D type
1908	NY210	Enterovirus D68	KP745757	7332	10/14/2014	Human	D type
1909	NY278	Enterovirus D68	KP745760	7332	09/19/2014	Human	D type
1910	NY316	Enterovirus D68	KP745764	7333	09/24/2014	Human	D type
1911	NY328	Enterovirus D68	KP745766	7333	09/26/2014	Human	D type
1912	NY329	Enterovirus D68	KP745767	7321	09/26/2014	Human	D type
1913	NYC403	Enterovirus D68	JX101846	7341	09/2009	Human	D type
1914	NZ-2010-541	Enterovirus D68	JX070222	7320	06/23/2010	Human	D type
1915	US/KY/14-18953	Enterovirus D68	KM851231	7348	08/2014	Human	D type

1916	ANG/2010-23293	Enterovirus D94	MT081370	7402	2010	Human	D type
1917	D94-NIG-14-534	Enterovirus D94	MW384880	7293	2014	Human	D type
1918	1-I8/TW/2008	Human enterovirus 68	KT318494	7330	2008	Human	D type
1919	14ISO0020050	Human enterovirus 68	MK105978	7304	10/17/2014	Human	D type
1920	14ISO0043	Human enterovirus 68	MK105976	7302	10/22/2014	Human	D type
1921	14ISO0120062	Human enterovirus 68	MK105979	7341	11/18/2014	Human	D type
1922	14ISO18022	Human enterovirus 68	MK105977	7339	12/02/2014	Human	D type
1923	16V0908R2	Human enterovirus 68	MK105980	7325	06/08/2016	Human	D type
1924	16V1106R2	Human enterovirus 68	MK105981	7326	06/10/2016	Human	D type
1925	2016-R3936	Human enterovirus 68	MH341711	7329	07/2016	Human	D type
1926	2016-R4010	Human enterovirus 68	MH341712	7329	07/2016	Human	D type
1927	2016-R4150	Human enterovirus 68	MH341726	7345	07/2016	Human	D type
1928	2016-R4428	Human enterovirus 68	MH341713	7329	08/2016	Human	D type
1929	2016-R4483	Human enterovirus 68	MH34171	7329	08/2016	Human	D type

			4				
1930	2016-R4523	Human enterovirus 68	MH34172 7	7345	08/2016	Human	D type
1931	2016-R4559	Human enterovirus 68	MH34172 8	7345	08/2016	Human	D type
1932	2016-R4607	Human enterovirus 68	MH34172 9	7345	08/2016	Human	D type
1933	2016-R4609	Human enterovirus 68	MH34171 5	7329	08/2016	Human	D type
1934	2016-R4839	Human enterovirus 68	MH34171 6	7329	08/2016	Human	D type
1935	2016-R4852	Human enterovirus 68	MH34171 7	7329	08/2016	Human	D type
1936	2016-R4855	Human enterovirus 68	MH34171 8	7329	08/2016	Human	D type
1937	2016-R4863	Human enterovirus 68	MH34171 9	7329	08/2016	Human	D type
1938	2016-R4934	Human enterovirus 68	MH34172 0	7329	08/2016	Human	D type
1939	2016-R5080	Human enterovirus 68	MH34173 0	7345	09/2016	Human	D type
1940	2016-R5147	Human enterovirus 68	MH34173 1	7345	09/2016	Human	D type
1941	2016-R5271	Human enterovirus 68	MH34172 1	7329	09/2016	Human	D type
1942	2016-R5733	Human enterovirus 68	MH04132	7334	10/2016	Human	D type

			3				
1943	2016-R5733	Human enterovirus 68	MH341722	7329	10/2016	Human	D type
1944	2016-R5734	Human enterovirus 68	MH041324	7334	10/2016	Human	D type
1945	2016-R5734	Human enterovirus 68	MH341723	7329	10/2016	Human	D type
1946	2016-R5928	Human enterovirus 68	MH341724	7329	10/2016	Human	D type
1947	2016-R6058	Human enterovirus 68	MH341732	7345	10/2016	Human	D type
1948	2016-R6091	Human enterovirus 68	MH341725	7329	10/2016	Human	D type
1949	2016-R6143	Human enterovirus 68	MH341733	7345	10/2016	Human	D type
1950	2017-R1150	Human enterovirus 68	MH341734	7345	02/2017	Human	D type
1951	2123800027	Human enterovirus 68	MK105982	7322	09/17/2012	Human	D type
1952	594-OsakaC-JPN-2015	Human enterovirus 68	LC107895	7279	08/15/2015	Human	D type
1953	639-OsakaC-JPN-2015	Human enterovirus 68	LC107896	7279	08/28/2015	Human	D type
1954	692-Osaka City-JPN-2013	Human enterovirus 68	LC068708	7341	09/24/2013	Human	D type
1955	705-Osaka City-JPN-2013	Human enterovirus 68	LC068709	7341	09/30/2	Human	D type

					013		
1956	726-Osaka City-JPN-2013	Human enterovirus 68	LC068710	7341	10/08/2013	Human	D type
1957	727-Osaka City-JPN-2013	Human enterovirus 68	LC068711	7293	10/04/2013	Human	D type
1958	A184-OsakaC-JPN-2015	Human enterovirus 68	LC107897	7279	07/27/2015	Human	D type
1959	A241-OsakaC-JPN-2015	Human enterovirus 68	LC107898	7196	08/07/2015	Human	D type
1960	A244-OsakaC-JPN-2015	Human enterovirus 68	LC107899	7279	09/11/2015	Human	D type
1961	A250-OsakaC-JPN-2015	Human enterovirus 68	LC107900	7112	09/17/2015	Human	D type
1962	A252-OsakaC-JPN-2015	Human enterovirus 68	LC107901	7110	09/18/2015	Human	D type
1963	BCH4235A	Human enterovirus 68	KT285485	7333	08/11/2014	Human	D type
1964	BCH824A	Human enterovirus 68	KT285484	7334	06/12/2008	Human	D type
1965	CF125132-FRA-2016	Human enterovirus 68	MT791934	7348	05/04/2016	Human	D type
1966	CF183054-FRA-2016	Human enterovirus 68	MT791933	7333	07/01/2016	Human	D type
1967	CF190038-FRA-2016	Human enterovirus 68	MT791932	7333	07/07/2016	Human	D type
1968	CF193002-FRA-2016	Human enterovirus 68	MT78973	7333	07/09/2	Human	D type

			4		016		
1969	CF193158-FRA-2016	Human enterovirus 68	MT78973 5	7332	07/11/20 16	Human	D type
1970	CF194006-FRA-2016	Human enterovirus 68	MT79193 0	7333	07/11/20 16	Human	D type
1971	CF195004-FRA-2016	Human enterovirus 68	MT78973 7	7333	07/12/2 016	Human	D type
1972	CF226028-FRA-2016	Human enterovirus 68	MT79192 9	7333	08/13/2 016	Human	D type
1973	CF241733-FRA-2016	Human enterovirus 68	MT79193 1	7334	10/12/2 016	Human	D type
1974	CF266116-FRA-2016	Human enterovirus 68	MT78973 6	7334	09/22/2 016	Human	D type
1975	CF270004-FRA-2016	Human enterovirus 68	MT79192 8	7333	09/25/2 016	Human	D type
1976	CF287798-FRA-2018	Human enterovirus 68	MT78975 1	7348	06/15/2 018	Human	D type
1977	CF307029-FRA-2014	Human enterovirus 68	MT79192 7	7332	11/02/20 14	Human	D type
1978	CF408376-FRA-2018	Human enterovirus 68	MT78975 2	7302	08/30/2 018	Human	D type
1979	CF408680-FRA-2018	Human enterovirus 68	MT78975 0	7348	08/30/2 018	Human	D type
1980	CF425016-FRA-2018	Human enterovirus 68	MT78974 9	7348	09/09/2 018	Human	D type
1981	CF425314-FRA-2018	Human enterovirus 68	MT78974	7333	09/10/2	Human	D type

			1		018		
1982	CF434664-FRA-2018	Human enterovirus 68	MT78974 8	7333	09/14/2 018	Human	D type
1983	CF473248-FRA-2018	Human enterovirus 68	MT78973 9	7333	10/08/2 018	Human	D type
1984	CF479745-FRA-2018	Human enterovirus 68	MT78974 3	7333	10/11/20 18	Human	D type
1985	CF482253-FRA-2018	Human enterovirus 68	MT78974 7	7333	10/12/2 018	Human	D type
1986	CF487798-FRA-2018	Human enterovirus 68	MT78974 0	7332	10/16/2 018	Human	D type
1987	CF551739-FRA-2018	Human enterovirus 68	MT78974 6	7333	11/23/20 18	Human	D type
1988	CF569939-FRA-2018	Human enterovirus 68	MT78973 8	7333	12/04/2 018	Human	D type
1989	CF572239-FRA-2018	Human enterovirus 68	MT78974 2	7332	12/05/2 018	Human	D type
1990	CF606093-FRA-2018	Human enterovirus 68	MT78974 4	7333	12/26/2 018	Human	D type
1991	CQ5313	Human enterovirus 68	KT764078	7349	08/22/2 013	Human	D type
1992	EV-D68/environment/Gainesville/1/2015	Human enterovirus 68	KU50999 7	7333	09/08/2 015	Human	D type
1993	EV-D68/Homo sapiens/CHN/DZH-ZJUC/2018	Human enterovirus 68	MK61408 7	7294	11/18/20 18	Human	D type
1994	EV-D68/Homo sapiens/USA/C2204/2003	Human enterovirus 68	KX25541	7285	09/17/2	Human	D type

			0		003		
1995	EV-D68/Homo sapiens/USA/C2206/2003	Human enterovirus 68	KX255372	7285	11/03/2003	Human	D type
1996	EV-D68/Homo sapiens/USA/C2386/2003	Human enterovirus 68	KX255388	7285	09/02/2003	Human	D type
1997	EV-D68/Homo sapiens/USA/C2590/2000	Human enterovirus 68	KX255405	7285	10/07/2000	Human	D type
1998	EV-D68/Homo sapiens/USA/C3256/2003	Human enterovirus 68	KX255400	7285	10/09/2003	Human	D type
1999	EV-D68/Homo sapiens/USA/C3486/2005	Human enterovirus 68	KX255393	7285	10/17/2005	Human	D type
2000	EV-D68/Homo sapiens/USA/C3578/2000	Human enterovirus 68	KX255357	7285	09/19/2000	Human	D type
2001	EV-D68/Homo sapiens/USA/C3963/2000	Human enterovirus 68	KX255353	7285	08/22/2000	Human	D type
2002	EV-D68/Homo sapiens/USA/C6261/2000	Human enterovirus 68	KX255391	7285	09/08/2000	Human	D type
2003	EV-D68/Homo sapiens/USA/C6633/2000	Human enterovirus 68	KX433166	7285	09/15/2000	Human	D type
2004	EV-D68/Homo sapiens/USA/C6832/2009	Human enterovirus 68	KX255402	7284	09/15/2009	Human	D type
2005	EV-D68/Homo sapiens/USA/C6840/2009	Human enterovirus 68	KX255408	7284	09/15/2009	Human	D type
2006	EV-D68/Homo sapiens/USA/C6944/2006	Human enterovirus 68	KX255363	7291	09/26/2006	Human	D type
2007	EV-D68/Homo sapiens/USA/C7245/2011	Human enterovirus 68	KX25537	7291	09/27/2	Human	D type

			6		011		
2008	EV-D68/Homo sapiens/USA/C7396/2012	Human enterovirus 68	KX25539<br>2	7291	08/19/2<br>012	Human	D type
2009	EV-D68/Homo sapiens/USA/C7413/2012	Human enterovirus 68	KX43316<br>4	7285	10/16/2<br>012	Human	D type
2010	EV-D68/Homo sapiens/USA/C7714/2014	Human enterovirus 68	KX25539<br>0	7285	07/20/2<br>014	Human	D type
2011	EV-D68/Homo sapiens/USA/C7715/2014	Human enterovirus 68	KX25536<br>2	7285	07/22/2<br>014	Human	D type
2012	EV-D68/Homo sapiens/USA/C7716/2014	Human enterovirus 68	KX25540<br>3	7285	07/26/2<br>014	Human	D type
2013	EV-D68/Homo sapiens/USA/C7720/2014	Human enterovirus 68	KX25535<br>2	7285	08/12/2<br>014	Human	D type
2014	EV-D68/Homo sapiens/USA/C7721/2014	Human enterovirus 68	KX25541<br>5	7285	08/14/2<br>014	Human	D type
2015	EV-D68/Homo sapiens/USA/C7722/2014	Human enterovirus 68	KX25536<br>4	7285	08/14/2<br>014	Human	D type
2016	EV-D68/Homo sapiens/USA/C7723/2014	Human enterovirus 68	KX25537<br>3	7285	08/16/2<br>014	Human	D type
2017	EV-D68/Homo sapiens/USA/C7729/2014	Human enterovirus 68	KX25537<br>8	7285	09/08/2<br>014	Human	D type
2018	EV-D68/Homo sapiens/USA/C7730/2014	Human enterovirus 68	KX255411	7285	09/11/20<br>14	Human	D type
2019	EV-D68/Homo sapiens/USA/C7731/2014	Human enterovirus 68	KX25535<br>4	7285	09/12/2<br>014	Human	D type
2020	EV-D68/Homo sapiens/USA/C7732/2014	Human enterovirus 68	KX25536	7285	09/12/2	Human	D type

			7		014		
2021	EV-D68/Homo sapiens/USA/C7733/2014	Human enterovirus 68	KX255385	7285	09/13/2014	Human	D type
2022	EV-D68/Homo sapiens/USA/C7743/2014	Human enterovirus 68	KX255407	7285	09/25/2014	Human	D type
2023	EV-D68/Homo sapiens/USA/C7745/2014	Human enterovirus 68	KX255389	7283	09/26/2014	Human	D type
2024	EV-D68/Homo sapiens/USA/C7787/2014	Human enterovirus 68	KX255382	7285	08/05/2014	Human	D type
2025	EV-D68/Homo sapiens/USA/C7788/2014	Human enterovirus 68	KX255413	7285	09/22/2014	Human	D type
2026	EV-D68/Homo sapiens/USA/C7789/2014	Human enterovirus 68	KX255395	7285	09/24/2014	Human	D type
2027	EV-D68/Homo sapiens/USA/C7791/2014	Human enterovirus 68	KX433161	7285	09/28/2014	Human	D type
2028	EV-D68/Homo sapiens/USA/C7793/2014	Human enterovirus 68	KX255379	7285	10/03/2014	Human	D type
2029	EV-D68/Homo sapiens/USA/MO61/2009	Human enterovirus 68	KX261820	7284	09/27/2009	Human	D type
2030	EV-D68/Homo sapiens/USA/MO62/2009	Human enterovirus 68	KX261807	7284	09/27/2009	Human	D type
2031	EV-D68/Homo sapiens/USA/MO63/2009	Human enterovirus 68	KX261794	7284	09/27/2009	Human	D type
2032	EV-D68/Homo sapiens/USA/MO64/2009	Human enterovirus 68	KX261808	7284	09/27/2009	Human	D type
2033	EV-D68/Homo sapiens/USA/MO65/2009	Human enterovirus 68	KX26179	7284	09/27/2	Human	D type

			9		009		
2034	EV-D68/Homo sapiens/USA/MO66/2009	Human enterovirus 68	KX261826	7284	09/27/2009	Human	D type
2035	EV-D68/Homo sapiens/USA/MO67/2009	Human enterovirus 68	KX261804	7293	10/05/2009	Human	D type
2036	EV-D68/Homo sapiens/USA/MO68/2009	Human enterovirus 68	KX261793	7284	10/05/2009	Human	D type
2037	EV-D68/Homo sapiens/USA/MO69/2009	Human enterovirus 68	KX261817	7284	10/05/2009	Human	D type
2038	EV-D68/Homo sapiens/USA/MO70/2009	Human enterovirus 68	KX261795	7284	10/05/2009	Human	D type
2039	EV-D68/Homo sapiens/USA/MO71/2009	Human enterovirus 68	KX261812	7284	10/05/2009	Human	D type
2040	EV-D68/Homo sapiens/USA/MO72/2009	Human enterovirus 68	KX261815	7284	10/05/2009	Human	D type
2041	EV-D68/Homo sapiens/USA/MO73/2009	Human enterovirus 68	KX261811	7284	10/17/2009	Human	D type
2042	EV-D68/Homo sapiens/USA/MO74/2009	Human enterovirus 68	KX261797	7284	10/17/2009	Human	D type
2043	EV-D68/Homo sapiens/USA/MO75/2009	Human enterovirus 68	KX261806	7284	08/22/2009	Human	D type
2044	EV-D68/Homo sapiens/USA/MO76/2009	Human enterovirus 68	KX261805	7284	08/22/2009	Human	D type
2045	EV-D68/Homo sapiens/USA/MO77/2009	Human enterovirus 68	KX261822	7284	09/01/2009	Human	D type
2046	EV-D68/Homo sapiens/USA/MO78/2009	Human enterovirus 68	KX26182	7282	09/05/2	Human	D type

			5		009		
2047	EV-D68/Homo sapiens/USA/MO79/2009	Human enterovirus 68	KX261798	7284	09/06/2009	Human	D type
2048	EV-D68/Homo sapiens/USA/MO80/2009	Human enterovirus 68	KX261802	7284	09/17/2009	Human	D type
2049	EV-D68/Homo sapiens/USA/MO81/2009	Human enterovirus 68	KX261810	7268	09/17/2009	Human	D type
2050	EV-D68/Homo sapiens/USA/MO82/2009	Human enterovirus 68	KX261801	7284	09/17/2009	Human	D type
2051	EV-D68/Homo sapiens/USA/MO83/2009	Human enterovirus 68	KX261809	7284	09/17/2009	Human	D type
2052	EV-D68/Homo sapiens/USA/MO84/2009	Human enterovirus 68	KX261819	7284	09/17/2009	Human	D type
2053	EV-D68/Homo sapiens/USA/MO85/2009	Human enterovirus 68	KX261803	7284	09/17/2009	Human	D type
2054	EV-D68/Homo sapiens/USA/MO86/2009	Human enterovirus 68	KX261818	7284	09/17/2009	Human	D type
2055	EV-D68/Homo sapiens/USA/MO87/2009	Human enterovirus 68	KX261823	7284	09/21/2009	Human	D type
2056	EV-D68/Homo sapiens/USA/MO88/2009	Human enterovirus 68	KX261816	7284	09/23/2009	Human	D type
2057	EV-D68/Homo sapiens/USA/MO89/2009	Human enterovirus 68	KX261796	7274	09/24/2009	Human	D type
2058	EV-D68/Homo sapiens/USA/MO90/2009	Human enterovirus 68	KX261800	7284	09/24/2009	Human	D type
2059	EV-D68/Homo sapiens/USA/MO91/2009	Human enterovirus 68	KX26182	7284	09/24/2	Human	D type

			1		009		
2060	EV-D68/Homo sapiens/USA/MO92/2009	Human enterovirus 68	KX261813	7284	09/23/2009	Human	D type
2061	EV-D68/Homo sapiens/USA/MO93/2009	Human enterovirus 68	KX261814	7284	09/23/2009	Human	D type
2062	EV-D68/Homo sapiens/USA/MO94/2009	Human enterovirus 68	KX261824	7293	09/23/2009	Human	D type
2063	EV-D68/Homo sapiens/USA/O1261a/2012	Human enterovirus 68	KX255361	7291	09/27/2012	Human	D type
2064	EV-D68/Homo sapiens/USA/O1312a/2012	Human enterovirus 68	KX255358	7291	10/10/2012	Human	D type
2065	EV-D68/Homo sapiens/USA/O270a/2012	Human enterovirus 68	KX255401	7285	08/27/2012	Human	D type
2066	EV-D68/Homo sapiens/USA/O418a/2012	Human enterovirus 68	KX255394	7282	09/14/2012	Human	D type
2067	EV-D68/Homo sapiens/USA/O433a/2012	Human enterovirus 68	KX255381	7291	09/04/2012	Human	D type
2068	EV-D68/Homo sapiens/USA/O4413/2013	Human enterovirus 68	KX255384	7285	10/01/2013	Human	D type
2069	EV-D68/Homo sapiens/USA/O4415/2013	Human enterovirus 68	KX255406	7285	09/25/2013	Human	D type
2070	EV-D68/Homo sapiens/USA/O4417/2013	Human enterovirus 68	KX255368	7285	09/11/2013	Human	D type
2071	EV-D68/Homo sapiens/USA/O4444/2013	Human enterovirus 68	KX433165	7283	10/15/2013	Human	D type
2072	EV-D68/Homo sapiens/USA/O4445/2013	Human enterovirus 68	KX25538	7285	10/07/2	Human	D type

			7		013		
2073	EV-D68/Homo sapiens/USA/O4446/2013	Human enterovirus 68	KX25539 8	7285	10/15/2 013	Human	D type
2074	EV-D68/Homo sapiens/USA/O466a/2012	Human enterovirus 68	KX25536 5	7285	09/05/2 012	Human	D type
2075	EV-D68/Homo sapiens/USA/O527a/2012	Human enterovirus 68	KX25538 0	7291	08/29/2 012	Human	D type
2076	EV-D68/Homo sapiens/USA/O541a/2012	Human enterovirus 68	KX25536 9	7291	08/21/2 012	Human	D type
2077	EV-D68/Homo sapiens/USA/O593a/2012	Human enterovirus 68	KX25538 6	7290	08/31/2 012	Human	D type
2078	EV-D68/Homo sapiens/USA/O622a/2012	Human enterovirus 68	KX25541 2	7285	09/26/2 012	Human	D type
2079	EV-D68/Homo sapiens/USA/O685a/2012	Human enterovirus 68	KX25540 4	7291	09/21/2 012	Human	D type
2080	EV-D68/Homo sapiens/USA/O774a/2012	Human enterovirus 68	KX25537 7	7285	09/03/2 012	Human	D type
2081	EV-D68/Homo sapiens/USA/O775a/2012	Human enterovirus 68	KX25541 4	7285	09/10/2 012	Human	D type
2082	EV-D68/Homo sapiens/USA/O810a/2012	Human enterovirus 68	KX25538 3	7285	09/04/2 012	Human	D type
2083	EV-D68/Homo sapiens/USA/O979a/2012	Human enterovirus 68	KX25535 9	7291	09/14/2 012	Human	D type
2084	EV-D68/Homo sapiens/USA/O980a/2012	Human enterovirus 68	KX25539 6	7291	09/21/2 012	Human	D type
2085	EV-D68/Homo sapiens/USA/U2215/2008	Human enterovirus 68	KX25536	7283	10/27/2	Human	D type

			6		008		
2086	EV-D68/Homo sapiens/USA/U2221/2008	Human enterovirus 68	KX255371	7284	10/14/2008	Human	D type
2087	EV-D68/Homo sapiens/USA/U2704/2009	Human enterovirus 68	KX255374	7291	08/26/2009	Human	D type
2088	EV-D68/Homo sapiens/USA/U2705/2009	Human enterovirus 68	KX255360	7291	08/26/2009	Human	D type
2089	EV-D68/Homo sapiens/USA/U2737/2009	Human enterovirus 68	KX255355	7291	09/11/2009	Human	D type
2090	EV-D68/Homo sapiens/USA/U2752/2009	Human enterovirus 68	KX255409	7291	09/04/2009	Human	D type
2091	EV-D68/Homo sapiens/USA/U4470/2012	Human enterovirus 68	KX255370	7285	08/09/2012	Human	D type
2092	EV-D68/Homo sapiens/USA/U4486/2012	Human enterovirus 68	KX255399	7285	08/02/2012	Human	D type
2093	EV-D68/Homo sapiens/USA/U4489/2012	Human enterovirus 68	KX255356	7285	08/15/2012	Human	D type
2094	EV-D68/Homo sapiens/USA/U5282/2014	Human enterovirus 68	KX255375	7285	09/26/2014	Human	D type
2095	EV-D68/Homo sapiens/USA/U797/2007	Human enterovirus 68	KX433167	7283	10/26/2007	Human	D type
2096	EV-D68/Homo sapiens/USA/U807/2007	Human enterovirus 68	KX255397	7285	10/22/2007	Human	D type
2097	EV-D68_STL_2016_19	Human enterovirus 68	KY292525	7332	2016	Human	D type
2098	EV-D68_STL_2016_20	Human enterovirus 68	KY29252	7332	2016	Human	D type

			6				
2099	EVD68/SZ01/CHN/2015	Human enterovirus 68	KU982558	7293	11/2015	Human	D type
2100	EVD68/SZ02/CHN/2015	Human enterovirus 68	KU982559	7293	11/2015	Human	D type
2101	EVD68/SZ03/CHN/2015	Human enterovirus 68	KU982560	7293	11/2015	Human	D type
2102	EVD68/SZ04/CHN/2015	Human enterovirus 68	KU982561	7293	12/2015	Human	D type
2103	Fermon	Human enterovirus 68	NC_038308 *	7367	-N/A-	Human	D type
2104	JPOC10-290	Human enterovirus 68	AB601882	7332	2010	Human	D type
2105	JPOC10-378	Human enterovirus 68	AB601883	7331	2010	Human	D type
2106	NIV1733258	Human enterovirus 68	MH330334	7324	09/14/2017	Human	D type
2107	NY10_16	Human enterovirus 68	KX957754	7321	07/10/2016	Human	D type
2108	NY126_16	Human enterovirus 68	KY385880	7290	08/10/2016	Human	D type
2109	NY135_16	Human enterovirus 68	KY385881	7332	08/13/2016	Human	D type
2110	NY141_16	Human enterovirus 68	KY385882	7334	08/16/2016	Human	D type
2111	NY149_16	Human enterovirus 68	KY38588	7320	08/20/2	Human	D type

			3		016		
2112	NY151_16	Human enterovirus 68	KY385884	7325	08/20/2016	Human	D type
2113	NY166_16	Human enterovirus 68	KY385885	7326	08/30/2016	Human	D type
2114	NY172_16	Human enterovirus 68	KY385886	7300	09/02/2016	Human	D type
2115	NY185_16	Human enterovirus 68	KY385887	7332	09/09/2016	Human	D type
2116	NY188_16	Human enterovirus 68	KY385888	7333	09/10/2016	Human	D type
2117	NY212_16	Human enterovirus 68	KY385889	7333	06/28/2016	Human	D type
2118	NY22_16	Human enterovirus 68	KX957755	7304	07/12/2016	Human	D type
2119	NY230_16	Human enterovirus 68	KY385890	7332	06/07/2016	Human	D type
2120	NY234_16	Human enterovirus 68	KY385891	7314	06/08/2016	Human	D type
2121	NY241_16	Human enterovirus 68	KY385892	7326	06/10/2016	Human	D type
2122	NY29_16	Human enterovirus 68	KX957756	7331	07/13/2016	Human	D type
2123	NY30_16	Human enterovirus 68	KX957757	7331	07/17/2016	Human	D type
2124	NY39_16	Human enterovirus 68	KX95775	7332	07/17/2	Human	D type

			8		016		
2125	NY43_16	Human enterovirus 68	KX957759	7331	07/18/2016	Human	D type
2126	NY44_16	Human enterovirus 68	KX957760	7310	07/23/2016	Human	D type
2127	Ny59_16	Human enterovirus 68	KX957761	7321	07/29/2016	Human	D type
2128	NY75_16	Human enterovirus 68	KX957762	7306	07/29/2016	Human	D type
2129	S0003LXT	Human enterovirus 68	MW567157	7347	-N/A-	Human	D type
2130	S0045ZHW	Human enterovirus 68	MW567158	7347	-N/A-	Human	D type
2131	S0092GHZ	Human enterovirus 68	MW567160	7347	-N/A-	Human	D type
2132	S0127XFS	Human enterovirus 68	MW567159	7347	-N/A-	Human	D type
2133	S0408QHG	Human enterovirus 68	MW567161	7347	-N/A-	Human	D type
2134	T106/Ft Jackson South Carolina USA/2002	Human enterovirus 68	KX384960	7201	11/30/2002	Human	D type
2135	take3	Human enterovirus 68	LC495298	7334	09/01/2018	Human	D type
2136	take4	Human enterovirus 68	LC495299	7334	10/18/2018	Human	D type
2137	TB5-16-Ph232	Human enterovirus 68	LC629445	7333	03/14/2	Human	D type

					016		
2138	TB5-16-Ph262	Human enterovirus 68	LC629446	7332	04/04/2<br>016	Human	D type
2139	TB5-17-Ph282	Human enterovirus 68	LC629447	7333	05/24/2<br>017	Human	D type
2140	TB5-18-Ph204	Human enterovirus 68	LC629448	7345	04/10/2<br>018	Human	D type
2141	TB5-18-Ph483	Human enterovirus 68	LC629449	7345	09/04/2<br>018	Human	D type
2142	TB5-18-Ph631	Human enterovirus 68	LC629450	7345	11/22/20<br>18	Human	D type
2143	TB6-15-Ph427	Human enterovirus 68	LC629443	7326	10/27/2<br>015	Human	D type
2144	TB8-15-Ph508	Human enterovirus 68	LC629444	7338	11/17/20<br>15	Human	D type
2145	TB9-15-Ph380	Human enterovirus 68	LC629442	7323	10/14/2<br>015	Human	D type
2146	TBp-13-Ph209	Human enterovirus 68	LC629441	7345	05/08/2<br>013	Human	D type
2147	TEv-13-Ph137	Human enterovirus 68	LC629439	7345	03/06/2<br>013	Human	D type
2148	TEv-13-Ph173	Human enterovirus 68	LC629440	7331	05/06/2<br>013	Human	D type
2149	TOp-12-Ph146	Human enterovirus 68	LC629438	7347	11/22/20<br>12	Human	D type
2150	TTa-08-Ph561	Human enterovirus 68	LC629436	7345	12/05/2	Human	D type

					008		
2151	TTa-11-Ph224	Human enterovirus 68	KX789259	7341	06/2011	Human	D type
2152	TTa-11-Ph272	Human enterovirus 68	LC629437	7326	06/25/2011	Human	D type
2153	TW-00785-2014	Human enterovirus 68	KT711083	7330	07/17/2014	Human	D type
2154	TW-00821-2014	Human enterovirus 68	KT711084	7330	08/01/2014	Human	D type
2155	TW-00880-2014	Human enterovirus 68	KT711078	7333	08/19/2014	Human	D type
2156	TW-00893-2014	Human enterovirus 68	KT711079	7330	08/22/2014	Human	D type
2157	TW-00898-2014	Human enterovirus 68	KT711082	7330	08/20/2014	Human	D type
2158	TW-00909-2014	Human enterovirus 68	KT711080	7330	09/02/2014	Human	D type
2159	TW-00928-2014	Human enterovirus 68	KT711085	7330	09/03/2014	Human	D type
2160	TW-00932-2014	Human enterovirus 68	KT711081	7330	09/11/2014	Human	D type
2161	TW-02512-2014	Human enterovirus 68	KT711086	7330	08/12/2014	Human	D type
2162	TW-02795-2014	Human enterovirus 68	KT711088	7330	09/10/2014	Human	D type
2163	TW-02809-2014	Human enterovirus 68	KT711087	7330	09/11/20	Human	D type

					14		
2164	US/MO/14-18949	Human enterovirus 68	MH708882	7311	2016	Human	D type
2165	USA/2018-23087	Human enterovirus 68	MK491180	7324	08/2018	Human	D type
2166	USA/2018-23088	Human enterovirus 68	MK491181	7324	08/2018	Human	D type
2167	USA/2018-23089	Human enterovirus 68	MK491182	7324	09/2018	Human	D type
2168	USA/2018-23201	Human enterovirus 68	MN389730	7333	2018	Human	D type
2169	USA/2018-23206	Human enterovirus 68	MN389731	7332	2018	Human	D type
2170	USA/2018-23209	Human enterovirus 68	MN389732	7332	2018	Human	D type
2171	USA/2018-23216	Human enterovirus 68	MN389733	7332	2018	Human	D type
2172	USA/2018-23252	Human enterovirus 68	MN389734	7332	2018	Human	D type
2173	USA/2018-23263	Human enterovirus 68	MN389735	7332	2018	Human	D type
2174	USA/2018-23272	Human enterovirus 68	MT081368	7332	2018	Human	D type
2175	USA/AK/2008-23112	Human enterovirus 68	MN240494	7341	2008	Human	D type
2176	USA/AL/2007-23110	Human enterovirus 68	MN24049	7341	2007	Human	D type

			2				
2177	USA/AL/2014-19169	Human enterovirus 68	KT995535	7292	08/29/2014	Human	D type
2178	USA/AL/2014-19241	Human enterovirus 68	KT995605	7292	09/15/2014	Human	D type
2179	USA/AL/2018-23212	Human enterovirus 68	MN246005	7324	2018	Human	D type
2180	USA/AR/2014-19227	Human enterovirus 68	KT995591	7292	09/14/2014	Human	D type
2181	USA/AZ/2018-23214	Human enterovirus 68	MN246007	7320	2018	Human	D type
2182	USA/CA/1962-23234	Human enterovirus 68	MN240508	7368	1962	Human	D type
2183	USA/CA/2002-23233	Human enterovirus 68	MN240507	7368	2002	Human	D type
2184	USA/CA/2003-23235	Human enterovirus 68	MN240509	7333	2003	Human	D type
2185	USA/CA/2014-RGDS-1024	Human enterovirus 68	MK681489	7342	09/2014	Human	D type
2186	USA/CA/2014-RGDS-1025	Human enterovirus 68	MK681490	7328	09/2014	Human	D type
2187	USA/CA/2014-RGDS-1026	Human enterovirus 68	MK681491	7330	09/2014	Human	D type
2188	USA/CA/2014-RGDS-1027	Human enterovirus 68	MK681492	7337	09/2014	Human	D type
2189	USA/CA/2014-RGDS-1028	Human enterovirus 68	MK68149	7334	10/2014	Human	D type

			3				
2190	USA/CA/2014-RGDS-1029	Human enterovirus 68	MK68149 4	7337	09/2014	Human	D type
2191	USA/CA/2014-RGDS-1030	Human enterovirus 68	MK68149 5	7337	10/2014	Human	D type
2192	USA/CA/2014-RGDS-1031	Human enterovirus 68	MK68149 6	7342	10/2014	Human	D type
2193	USA/CA/2014-RGDS-1032	Human enterovirus 68	MK68149 7	7330	09/2014	Human	D type
2194	USA/CA/2014-RGDS-1033	Human enterovirus 68	MK68149 8	7321	09/2014	Human	D type
2195	USA/CA/2014-RGDS-1034	Human enterovirus 68	MK68149 9	7330	10/2014	Human	D type
2196	USA/CA/2014-RGDS-1035	Human enterovirus 68	MK68150 0	7329	10/2014	Human	D type
2197	USA/CA/2014-RGDS-1036	Human enterovirus 68	MK68150 1	7328	10/2014	Human	D type
2198	USA/CA/2014-RGDS-1037	Human enterovirus 68	MK68150 2	7329	11/2014	Human	D type
2199	USA/CA/2014-RGDS-1038	Human enterovirus 68	MK68150 3	7338	10/2014	Human	D type
2200	USA/CA/2014-RGDS-1039	Human enterovirus 68	MK68150 4	7338	11/2014	Human	D type
2201	USA/CA/2014-RGDS-1040	Human enterovirus 68	MK68150 5	7338	12/2014	Human	D type
2202	USA/CO/2014-19175	Human enterovirus 68	KT995533	7292	08/27/2	Human	D type

					014		
2203	USA/CO/2014-19189	Human enterovirus 68	KT995540	7292	09/03/2014	Human	D type
2204	USA/CO/2014-19190	Human enterovirus 68	KT995541	7292	09/05/2014	Human	D type
2205	USA/CO/2014-19244	Human enterovirus 68	KT995608	7292	09/15/2014	Human	D type
2206	USA/CO/2014-19258	Human enterovirus 68	KT995623	7292	09/13/2014	Human	D type
2207	USA/CO/2016-23290	Human enterovirus 68	MN259119	7321	2016	Human	D type
2208	USA/CO/2018-23087	Human enterovirus 68	MN245981	7325	2018	Human	D type
2209	USA/CO/2018-23163	Human enterovirus 68	MN245985	7325	2018	Human	D type
2210	USA/CO/2018-23164	Human enterovirus 68	MN245986	7324	2018	Human	D type
2211	USA/CO/2018-23165	Human enterovirus 68	MN245987	7324	2018	Human	D type
2212	USA/CO/2018-23166	Human enterovirus 68	MN245988	7332	2018	Human	D type
2213	USA/CO/2018-23182	Human enterovirus 68	MN245989	7324	2018	Human	D type
2214	USA/CT/2014-19180	Human enterovirus 68	KT995543	7292	09/09/2014	Human	D type
2215	USA/CT/2014-19201	Human enterovirus 68	KT995567	7292	09/13/2	Human	D type

					014		
2216	USA/CT/2014-19202	Human enterovirus 68	KT995568	7292	09/14/2014	Human	D type
2217	USA/CT/2014-19208	Human enterovirus 68	KT995575	7292	09/12/2014	Human	D type
2218	USA/CT/2014-19230	Human enterovirus 68	KT995594	7292	09/16/2014	Human	D type
2219	USA/CT/2014-19233	Human enterovirus 68	KT995597	7291	09/15/2014	Human	D type
2220	USA/DC/2014-19264	Human enterovirus 68	KT995629	7292	09/09/2014	Human	D type
2221	USA/DE/2014-19197	Human enterovirus 68	KT995563	7292	09/09/2014	Human	D type
2222	USA/FL/2014-19070	Human enterovirus 68	KT995611	7292	2014	Human	D type
2223	USA/FL/2016-19504	Human enterovirus 68	KX675261	7333	03/31/2016	Human	D type
2224	USA/FL/2016-23278	Human enterovirus 68	MN259107	7314	2016	Human	D type
2225	USA/FL/2016-23279	Human enterovirus 68	MN259108	7317	2016	Human	D type
2226	USA/GA/2014-19200	Human enterovirus 68	KT995566	7292	09/09/2014	Human	D type
2227	USA/GA/2014-19246	Human enterovirus 68	KT995610	7292	09/24/2014	Human	D type
2228	USA/GA/2018-23218	Human enterovirus 68	MN246011	7324	2018	Human	D type

2229	USA/IA/2014-19171	Human enterovirus 68	KT995548	7292	09/10/2014	Human	D type
2230	USA/IA/2014-19225	Human enterovirus 68	KT995589	7292	2014	Human	D type
2231	USA/ID/2014-19068	Human enterovirus 68	KT995555	7293	09/08/2014	Human	D type
2232	USA/ID/2014-19224	Human enterovirus 68	KT995588	7293	2014	Human	D type
2233	USA/ID/2014-19256	Human enterovirus 68	KT995621	7292	09/25/2014	Human	D type
2234	USA/IL/2014-18956	Human enterovirus 68	MK268345	7292	08/2014	Human	D type
2235	USA/IL/2014-19179	Human enterovirus 68	KT995542	7292	09/06/2014	Human	D type
2236	USA/IL/2014-19199	Human enterovirus 68	KT995565	7290	08/22/2014	Human	D type
2237	USA/IL/2018-23252	Human enterovirus 68	MN246015	7332	2018	Human	D type
2238	USA/IN/2009-23228	Human enterovirus 68	MN240504	7332	2009	Human	D type
2239	USA/IN/2009-23240	Human enterovirus 68	MN240513	7332	2009	Human	D type
2240	USA/IN/2009-23241	Human enterovirus 68	MN240514	7332	2009	Human	D type
2241	USA/KS/2007-23111	Human enterovirus 68	MN240493	7341	2007	Human	D type
2242	USA/KS/2007-23239	Human enterovirus 68	MN240512	7341	2007	Human	D type

2243	USA/KS/2014-19234	Human enterovirus 68	KT995598	7292	09/15/2014	Human	D type
2244	USA/KY/2014-19167	Human enterovirus 68	KT995532	7292	08/22/2014	Human	D type
2245	USA/KY/2014-19182	Human enterovirus 68	KT995545	7292	09/07/2014	Human	D type
2246	USA/ME/2016-23286	Human enterovirus 68	MN259115	7321	2016	Human	D type
2247	USA/MI/2014-19198	Human enterovirus 68	KT995564	7292	09/08/2014	Human	D type
2248	USA/MI/2014-19221	Human enterovirus 68	KT995585	7292	09/12/2014	Human	D type
2249	USA/MI/2014-19238	Human enterovirus 68	KT995602	7292	09/06/2014	Human	D type
2250	USA/MI/2014-19251	Human enterovirus 68	KT995616	7292	09/15/2014	Human	D type
2251	USA/MN/1989-23220	Human enterovirus 68	MN240496	7345	1989	Human	D type
2252	USA/MN/2018-23204	Human enterovirus 68	MN245997	7324	2018	Human	D type
2253	USA/MN/2018-23205	Human enterovirus 68	MN245998	7324	2018	Human	D type
2254	USA/MN/2018-23263	Human enterovirus 68	MN246026	7324	2018	Human	D type
2255	USA/MO/2000-23221	Human enterovirus 68	MN240497	7333	2000	Human	D type

2256	USA/NY/2016-19505	Human enterovirus 68	KX67526 2	7319	06/24/2 016	Human	D type
2257	USA/NY/2018-23215	Human enterovirus 68	MN24600 8	7324	2018	Human	D type
2258	USA/NY/2018-23216	Human enterovirus 68	MN24600 9	7324	2018	Human	D type
2259	USA/NY/2018-23219	Human enterovirus 68	MN24601 2	7324	2018	Human	D type
2260	USA/OH/2014-19192	Human enterovirus 68	KT995557	7292	08/29/2 014	Human	D type
2261	USA/OH/2014-19223	Human enterovirus 68	KT995587	7292	2014	Human	D type
2262	USA/OH/2014-19240	Human enterovirus 68	KT995604	7292	09/17/2 014	Human	D type
2263	USA/OH/2016-23291	Human enterovirus 68	MN25912 0	7319	2016	Human	D type
2264	USA/OH/2018-23088	Human enterovirus 68	MN24598 2	7324	2018	Human	D type
2265	USA/OH/2018-23253	Human enterovirus 68	MN24601 6	7324	2018	Human	D type
2266	USA/OH/2018-23254	Human enterovirus 68	MN24601 7	7324	2018	Human	D type
2267	USA/OH/2018-23255	Human enterovirus 68	MN24601 8	7323	2018	Human	D type
2268	USA/OH/2018-23256	Human enterovirus 68	MN24601 9	7324	2018	Human	D type
2269	USA/OH/2018-23257	Human enterovirus 68	MN24602	7324	2018	Human	D type

			0				
2270	USA/OH/2018-23258	Human enterovirus 68	MN246021	7324	2018	Human	D type
2271	USA/OH/2018-23260	Human enterovirus 68	MN246023	7324	2018	Human	D type
2272	USA/OH/2018-23261	Human enterovirus 68	MN246024	7324	2018	Human	D type
2273	USA/OK/2014-19170	Human enterovirus 68	KT995536	7292	08/17/2014	Human	D type
2274	USA/OK/2014-19247	Human enterovirus 68	KT995612	7292	09/10/2014	Human	D type
2275	USA/OK/2018-23200	Human enterovirus 68	MN245993	7324	2018	Human	D type
2276	USA/OR/2014-19073	Human enterovirus 68	KT995595	7292	09/14/2014	Human	D type
2277	USA/PA/2014-19035	Human enterovirus 68	KT995537	7289	09/02/2014	Human	D type
2278	USA/PA/2014-19177	Human enterovirus 68	KT995538	7292	09/08/2014	Human	D type
2279	USA/PA/2014-19263	Human enterovirus 68	KT995628	7292	09/09/2014	Human	D type
2280	USA/PA/2016-23285	Human enterovirus 68	MN259114	7321	2016	Human	D type
2281	USA/PA/2016-23289	Human enterovirus 68	MN259118	7319	2016	Human	D type
2282	USA/PA/2018-23203	Human enterovirus 68	MN24599	7324	2018	Human	D type

			6				
2283	USA/RI/2014-19203	Human enterovirus 68	KT995569	7292	08/28/2014	Human	D type
2284	USA/RI/2014-19213	Human enterovirus 68	KT995580	7292	09/12/2014	Human	D type
2285	USA/RI/2014-19214	Human enterovirus 68	KT995581	7293	09/13/2014	Human	D type
2286	USA/SC/2014-19196	Human enterovirus 68	KT995562	7292	09/08/2014	Human	D type
2287	USA/SC/2014-19215	Human enterovirus 68	KT995582	7292	09/03/2014	Human	D type
2288	USA/SC/2014-19239	Human enterovirus 68	KT995603	7292	09/12/2014	Human	D type
2289	USA/SC/2016-23288	Human enterovirus 68	MN259117	7321	2016	Human	D type
2290	USA/SD/2014-19193	Human enterovirus 68	KT995559	7292	09/08/2014	Human	D type
2291	USA/SD/2014-19194	Human enterovirus 68	KT995560	7292	09/05/2014	Human	D type
2292	USA/SD/2014-19207	Human enterovirus 68	KT995573	7292	09/13/2014	Human	D type
2293	USA/SD/2014-19249	Human enterovirus 68	KT995614	7292	09/23/2014	Human	D type
2294	USA/TN/2014-19260	Human enterovirus 68	KT995625	7292	09/27/2014	Human	D type
2295	USA/TN/2018-23213	Human enterovirus 68	MN24600	7324	2018	Human	D type

			6				
2296	USA/TX/2002-23222	Human enterovirus 68	MN240498	7333	2002	Human	D type
2297	USA/TX/2003-23223	Human enterovirus 68	MN240499	7341	2003	Human	D type
2298	USA/TX/2003-23224	Human enterovirus 68	MN240500	7333	2003	Human	D type
2299	USA/TX/2014-19185	Human enterovirus 68	KT995552	7292	08/04/2014	Human	D type
2300	USA/TX/2014-19228	Human enterovirus 68	KT995592	7292	09/09/2014	Human	D type
2301	USA/TX/2014-19261	Human enterovirus 68	KT995626	7292	09/30/2014	Human	D type
2302	USA/TX/2016-19506	Human enterovirus 68	KX675263	7310	06/24/2016	Human	D type
2303	USA/TX/2016-23280	Human enterovirus 68	MN259109	7321	2016	Human	D type
2304	USA/TX/2018-23202	Human enterovirus 68	MN245995	7324	2018	Human	D type
2305	USA/TX/2018-23208	Human enterovirus 68	MN246001	7324	2018	Human	D type
2306	USA/UT/2014-19195	Human enterovirus 68	KT995561	7293	08/18/2014	Human	D type
2307	USA/VA/2014-19178	Human enterovirus 68	KT995539	7292	09/08/2014	Human	D type
2308	USA/VA/2014-19181	Human enterovirus 68	KT995544	7292	09/09/2	Human	D type

					014		
2309	USA/VA/2014-19187	Human enterovirus 68	KT995554	7292	09/09/2014	Human	D type
2310	USA/VA/2014-19218	Human enterovirus 68	KT995574	7292	09/12/2014	Human	D type
2311	USA/VA/2014-19255	Human enterovirus 68	KT995620	7293	09/18/2014	Human	D type
2312	USA/VT/2014-19245	Human enterovirus 68	KT995609	7292	09/18/2014	Human	D type
2313	USA/VT/2014-19259	Human enterovirus 68	KT995624	7292	10/01/2014	Human	D type
2314	USA/WA/2014-19060	Human enterovirus 68	KT995578	7292	09/14/2014	Human	D type
2315	USA/WA/2014-19184	Human enterovirus 68	KT995551	7292	09/10/2014	Human	D type
2316	USA/WA/2014-19210	Human enterovirus 68	KT995577	7292	09/09/2014	Human	D type
2317	USA/WA/2018-23201	Human enterovirus 68	MN245994	7324	2018	Human	D type
2318	USA/WI/2006-23109	Human enterovirus 68	MN240491	7341	2006	Human	D type
2319	USA/WI/2006-23227	Human enterovirus 68	MN240503	7341	2006	Human	D type
2320	USA/WI/2009-23230	Human enterovirus 68	MN240506	7341	2009	Human	D type
2321	USA/WI/2009-23245	Human enterovirus 68	MN24051	7333	2009	Human	D type

			7				
2322	USA/WI/2009-23247	Human enterovirus 68	MN240518	7341	2009	Human	D type
2323	USA/WI/2009-23248	Human enterovirus 68	MN240519	7341	2009	Human	D type
2324	USA/WI/2014-19217	Human enterovirus 68	KT995558	7292	09/09/2014	Human	D type
2325	USA/WI/2014-19222	Human enterovirus 68	KT995586	7254	09/09/2014	Human	D type
2326	USA/WI/2014-19226	Human enterovirus 68	KT995590	7292	09/11/2014	Human	D type
2327	USA/WV/2014-19242	Human enterovirus 68	KT995606	7292	09/16/2014	Human	D type
2328	USA/WY/2014-19051	Human enterovirus 68	KT995601	7293	09/11/2014	Human	D type
2329	USA/WY/2014-19253	Human enterovirus 68	KT995618	7292	09/24/2014	Human	D type
2330	VRES189236	Human enterovirus 68	MK105983	7281	08/18/2018	Human	D type
2331	VRES189536	Human enterovirus 68	MK105984	7326	09/09/2018	Human	D type
2332	VRES189539	Human enterovirus 68	MK105985	7326	09/10/2018	Human	D type
2333	VRES189550	Human enterovirus 68	MK105986	7304	09/10/2018	Human	D type
2334	VRES189560	Human enterovirus 68	MK10598	7341	09/10/2	Human	D type

			7		018		
2335	VRES189627	Human enterovirus 68	MK10598 8	7326	09/14/2 018	Human	D type
2336	VRES189781	Human enterovirus 68	MK10598 9	7341	09/21/2 018	Human	D type
2337	WZ17226	Human enterovirus 68	MZ82422 1	7335	10/12/2 018	Human	D type
2338	YC17106	Human enterovirus 68	MZ82422 2	7335	10/26/2 017	Human	D type
2339	YC18116	Human enterovirus 68	MZ82422 3	7335	09/04/2 018	Human	D type
2340	YC18137	Human enterovirus 68	MZ82422 4	7331	09/19/2 018	Human	D type
2341	J670/71	Human enterovirus 70	D00820	7390	-N/A-	Human	D type
2342	J670/71	Human enterovirus 70	DQ20117 7	7391	-N/A-	Human	D type
2343	JPN/1971-23330	Human enterovirus 70	MT08137 8	7391	1971	Human	D type
2344	JPN/1989-23292	Human enterovirus 70	MT08136 9	7390	1989	Human	D type
2345	44670	Human enterovirus 94	EF107097	7331	-N/A-	Human	D type
2346	E210	Human enterovirus 94	DQ91637 6	7364	-N/A-	Human	D type
2347	ANG/2010-23294	Human enterovirus D111	MT08137 1	7403	2010	Human	D type
2348	ANG/2010-23295	Human enterovirus D111	MT08137	7403	2010	Human	D type

			2				
2349	D111-NGR-KAT-1263	Human enterovirus D111	MW38488 1	7315	2017	Human	D type
